# Ethnic minorities' language attitudes toward heritage dialects and official standard languages: a narrative review across cognitive, affective, and behavioral dimensions

**DOI:** 10.3389/fpsyg.2026.1818757

**Published:** 2026-09-28

**Authors:** Wei Chen, Yok Fee Lee, Ratna Roshida Binti Ab. Razak, Arfah Binti Ab. Majid, Guopeng Lv

**Affiliations:** 1 Department of Government and Civilization Studies, Faculty of Human Ecology, Universiti Putra Malaysia, Serdang, Malaysia; 2 School of Marxism, Zhaotong University, Zhaotong, Yunnan, China

**Keywords:** language attitudes, heritage language, cognitive-affective-behavioral dimensions, structured ambivalence, narrative review, ethnic minorities

## Abstract

Language attitudes among ethnic minority communities have been examined extensively, yet most studies focus on single dimensions rather than on how cognitive, affective, and behavioral orientations interact to form coherent attitudinal configurations. This narrative review synthesizes 18 studies published between 2015 and 2026 to characterize the cognitive, affective, and behavioral dimensions of ethnic minorities' attitudes toward heritage dialects and official standard languages, and to identify the stable patterns emerging from dimensional interaction. Analysis reveals five recurrent themes within each dimension, all modulated by standardization strategy type and ethnic identity strength. The dimensions interact through four recursive pathways: cognition shapes affect, affect drives behavior, and behavior feeds back into both cognition and affect. These interactions produce three distinct stable configurations: assimilation, resistance, and structured ambivalence. Among them, structured ambivalence (including its domain-differentiated subtype) is the most frequently observed. Contrary to the assumption that intermediate states are merely transitional, domain-differentiated code choice acts as a psychological buffer, sustaining conflicting attitudes across time. Two intervention studies further demonstrate that critical language awareness and translanguaging pedagogy can redirect ambivalent configurations toward resistance. The review proposes an integrated “three dimensions–four pathways–three patterns” framework that consolidates fragmented research and identifies methodological gaps, particularly the underrepresentation of behavioral data and the scarcity of longitudinal evidence on pattern transition.

## 1 Introduction

Language standardization has become a defining feature of the contemporary nation-state. Across public education, administration, and the media, standard language ideologies typically install a single codified variety as the legitimate medium of formal communication, while positioning other varieties as informal, deficient, or merely local (Kroskrity, 1997; Milroy, 2002). Language, however, is never only an instrument of communication. For ethnolinguistic communities it is also a primary vehicle of group identity, intergenerational memory, and cultural continuity (May, 2005). Ethnic minorities therefore inhabit a structurally dual ecology. They are expected to acquire and display competence in the official standard language in order to access schooling, socioeconomic mobility, and civic participation, yet heritage dialects remain the intimate codes through which belonging, kinship, and cultural transmission are enacted. This dual positioning generates persistent tensions among language choice, identity negotiation, and cultural survival. Systematic inquiry into minorities' language attitudes is consequently of both scholarly and practical consequence. Attitudes have long been shown to shape language learning, use, and maintenance (Gardner, 1972), and they bear directly on the protection of linguistic diversity, equity in higher education, intergroup cohesion, and the design of cultural-rights policy (Hornberger and Korne, 2023; May, 2005). A substantial literature has addressed these issues. What remains insufficiently integrated is the internal architecture of such attitudes and the interactive mechanisms among their constituent dimensions.

For conceptual clarity, four terms are defined operationally and used consistently throughout this review. Heritage dialects denote the locally rooted, intergenerationally transmitted language varieties of ethnic minority communities. They function as core markers of communal identity and cultural belonging, encompass related notions such as the vernacular and the ethnolect, and combine everyday communicative utility with symbolic identity work (Leeman and Showstack, 2022). Official standard language refers to the codified variety that is institutionally established at the level of the state and invested with normative legitimacy in public and formal domains. It is the dominant language of education, administration, and the media, and it carries overt institutional prestige together with instrumental value (May, 2005; Milroy, 2002). Ethnic minorities are racial or ethnolinguistic groups that occupy a numerically and institutionally minoritized position within a country's mainstream language ecology. The category comprises Indigenous peoples, long-settled autochthonous minorities, and immigrant minorities, whose shared defining feature is that their heritage variety does not belong to the official standard-language system. Speakers of regional dialects within a majority nationality—for example, Cantonese or Hokkien speakers among the Han Chinese—are excluded from this definition. The three-dimensional attitudinal framework decomposes language attitudes into a cognitive dimension (beliefs and evaluations), an affective dimension (emotions and belonging), and a behavioral dimension (practices and choices), treating these components as interrelated rather than as isolated constructs (Ostrom, 1969).

Scholarship on language attitudes has evolved through three theoretically successive stages. In the first stage, research concentrated on unidimensional prestige stratification. Standard varieties were shown to attract overt institutional prestige, whereas non-standard dialects were routinely assigned low status in public evaluation (Kroskrity, 1997; Milroy, 2002). This unidimensional account, however, left a central puzzle unresolved: why stigmatized non-standard varieties continue to be used and valued in everyday life. The second stage responded with a dual cognitive–affective framework. Drawing on the notion of covert prestige, sociolinguistic research demonstrated that vernacular varieties carry identity-affirming value within their communities even when publicly devalued, producing a bifurcated pattern in which overt prestige accrues to the standard and covert prestige to the dialect (Labov, 1972). The affective and identity dimensions of language attitudes thereby entered the analytical repertoire alongside beliefs about status and vitality (Allard and Landry, 1986). Yet this dual framework remained largely descriptive: it rarely incorporated the behavioral dimension of actual language practice and choice, nor did it specify how its components interact. The third stage extended the framework into a three-dimensional model comprising cognitive, affective, and behavioral components (Ostrom, 1969; Santello, 2015), an approach that increasingly informs the measurement and modeling of attitudes in bilingual and minoritized settings (Tamburelli et al., 2025). Within this stage, cross-contextual empirical research has repeatedly documented a characteristic configuration that we term structured ambivalence: minority speakers cognitively endorse the formal legitimacy and instrumental value of the standard language, affectively remain attached to the identity value and communal belonging embodied in their heritage dialect, and behaviorally engage in domain-differentiated code choice that sustains both varieties in complementary spheres (Yin and Li, 2021; Zhu, 2015). This pattern is best understood as a stable attitude type observed in the literature rather than as a presupposed outcome of the present review.

It is equally important to acknowledge countervailing evidence. Language revitalization movements have in some settings reversed established prestige hierarchies and restored public legitimacy to minoritized varieties (Hornberger and Korne, 2023; May, 2003); some communities actively resist assimilation to the standard (Ward, 2025); and in particular polities heritage varieties have gained recognition in the public domain (Dorian, 2010; Screti, 2026). Taken together, these findings indicate that language attitudes are dynamic and context-bound rather than fixed, and that the interplay among the three dimensions must be examined in relation to the institutional and ideological settings in which speakers are situated.

Against this backdrop, three limitations in the existing literature motivate the present review. First, dimensional integration remains scarce. Although the tripartite structure of attitudes is well established, much empirical work attends to a single component—such as proficiency-linked attitudes toward a heritage language or status perceptions of a standard variety—without locating the findings within a unified framework (Alsahafi, 2026; Li and Wei, 2025). The overall architecture of minority language attitudes and the relations among their components are therefore difficult to assess. Second, contextual breadth is limited. Highly informative studies typically examine one country, one community, and one point in time—for example, heritage-language maintenance among the Hausa in Saudi Arabia (Alsahafi, 2026), citizenship-related language use in Switzerland (Masiero, 2026), or family language attitudes among the Sibe in north-western China (Yin and Li, 2021). While invaluable, such single-context designs constrain the capacity to distinguish community-specific dynamics from more general mechanisms. Third, mechanism-level explanation is underdeveloped. Structured ambivalence and related patterns have been identified and described across settings (Stites, 1999; Zhu, 2015), but the processes through which the cognitive, affective, and behavioral dimensions interact—and the contextual factors that condition these interactions—remain largely unspecified.

Furthermore, significant shifts in language ecologies and minority language policies over the past decade call for renewed synthesis, and the present review addresses these gaps by integrating empirical and theoretical research across multiple continents and sociolinguistic contexts. It maps the characteristics of minority language attitudes along the cognitive, affective, and behavioral dimensions, and it unpacks the interactive dynamics through which these dimensions coalesce into stable attitudinal patterns. Structured ambivalence is examined in this light as a documented configuration whose formation mechanisms the review seeks to explain. Accordingly, two research questions (RQs) guide this review:

RQ1: *What are the key characteristics of ethnic minorities' language attitudes toward heritage dialects and official standard languages across the cognitive, affective, and behavioral dimensions?*

RQ2: *How do these three dimensions interact with one another to form stable attitudinal patterns such as structured ambivalence?*

## 2 Methodology

This study uses a narrative review design to synthesize empirical and conceptual research. The screening procedures were adapted from the PRISMA-ScR (Preferred Reporting Items for Systematic Reviews and Meta-Analyses extension for Scoping Reviews) guidelines (Peters et al., 2022).

### 2.1 Sources and methods of data retrieval

Literature searches were conducted in three core academic databases: Web of Science Core Collection, Scopus, and Google Scholar. A Boolean search strategy was devised to retrieve relevant studies concerning ethnic minority groups, language-attitude constructs, and the three-dimensional analytical framework. The complete search string is presented below:

(“ethnic minority” OR “racial minority” OR “indigenous people” OR “immigrant ethnic group” OR “racialized minority”) AND (“language attitudes” OR “linguistic attitudes” OR “language evaluation” OR “language perception” OR “implicit attitudes” OR “explicit attitudes”) AND (“official standard language” OR “national standard language” OR “standard variety”) AND (“heritage dialect” OR “ethnic dialect” OR “minority language” OR “heritage language” OR “vernacular”) AND (“cognitive” OR “affective” OR “behavioral” OR “language behavior” OR “language revitalization” OR “language emancipation”)

The search was limited to studies published in English between January 1, 2015, and July 31, 2026. This 11-year timeframe was selected to ensure the timeliness of the evidence base, capture contemporary shifts in language ecologies and minority language policies, and respond to calls for focus on recent empirical developments in the field. No document type restrictions were applied at the initial retrieval stage; all records were carried forward for standardized screening in subsequent phases.

### 2.2 Eligibility criteria for selection

Two sequential sets of eligibility criteria were applied to screen the retrieved records. Records were first screened against universal publication standards. The full inclusion and exclusion criteria are outlined in Table 1.

**Table 1:** General publication inclusion and exclusion criteria.

Inclusion criteria	Exclusion criteria
1. Peer-reviewed original journal articles, including empirical studies, theoretical analyses, conceptual research, and scholarly opinion/perspective articles containing substantive conceptual argumentation 2. Full-text available in English 3. Published between January 1, 2015 and July 31, 2026	1. Non-peer-reviewed materials: book chapters, conference abstracts, policy briefs, gray literature, brief editorials, book reviews, and commentary articles without substantive conceptual or empirical content 2. Review articles/literature reviews (only primary studies included to avoid double synthesis bias) 3. Publications not available in full English 4. Publications published outside the 2015–2026 time range 5. Duplicate publications or studies with highly overlapping data and findings

Article eligibility was determined by substantive content rather than publisher article-type labels. Articles classified as “Opinion,” “Perspective,” or “Commentary” were included if they contained substantive conceptual analysis, theoretical argumentation, or empirical data and underwent formal peer review; they were excluded only if they constituted brief editorials, book reviews, or non-peer-reviewed commentary without substantive academic content.

Records were further assessed against a PCC (Population-Concept-Context) eligibility framework (Arksey and O'Malley, 2005). The framework was tailored to the specific scope of this review to filter studies based on study population, core conceptual focus, and research context. Table 2 presents the full PCC eligibility criteria.

**Table 2:** PCC Eligibility framework.

Criterion	Inclusion criteria	Exclusion criteria
Population	1. Studies explicitly focus on racial/ethnic minority groups, including indigenous peoples, immigrant ethnic groups, racialized minorities, and statutorily recognized ethnic minority groups within a sovereign state	1. Studies focus on intra-ethnic regional dialect speakers (e.g., Cantonese-speaking Han Chinese in Hong Kong, Mandarin/dialect speakers among Han Chinese in Singapore)—internal language variants within the same ethnic group, not ethnic/racial minorities
	2. Studies examine these groups' attitudes toward both their own heritage dialect/ethnic language and the national official standard language	2. Studies target the general population without separate subgroup analysis for ethnic minorities
		3. Studies group participants only by language proficiency, not by ethnic/racial identity
Concept	1. Core research topic is language attitudes, covering at least one of the three dimensions: cognitive, affective, and behavioral	1. Studies only examine language acquisition, phonetics, grammar, or pedagogical interventions, with no attitude/evaluation component
	2. Studies include bidirectional attitude comparison between heritage dialects and the official standard language	2. Studies only investigate one single language variety, with no dialect vs. standard comparison
		3. Studies only analyze language policy texts without measuring or analyzing group attitudes
Context	1. Studies are set in social/institutional contexts with an established official standard language (education, public administration, community, labor market, etc.) 2. No geographic restriction; all national/regional contexts are acceptable	1. Studies limited to purely private/family settings with no institutional or societal context 2. Studies set in fully equal multilingual contexts with no dominant official standard language and no prestige hierarchy

### 2.3 Screening implementation

As shown in Figure 1, all screening procedures were conducted independently by two researchers. Discrepancies were resolved through team discussion and consensus to minimize subjective bias and ensure inter-coder reliability. The screening process proceeded in three sequential phases, as detailed below.

**Figure 1:**
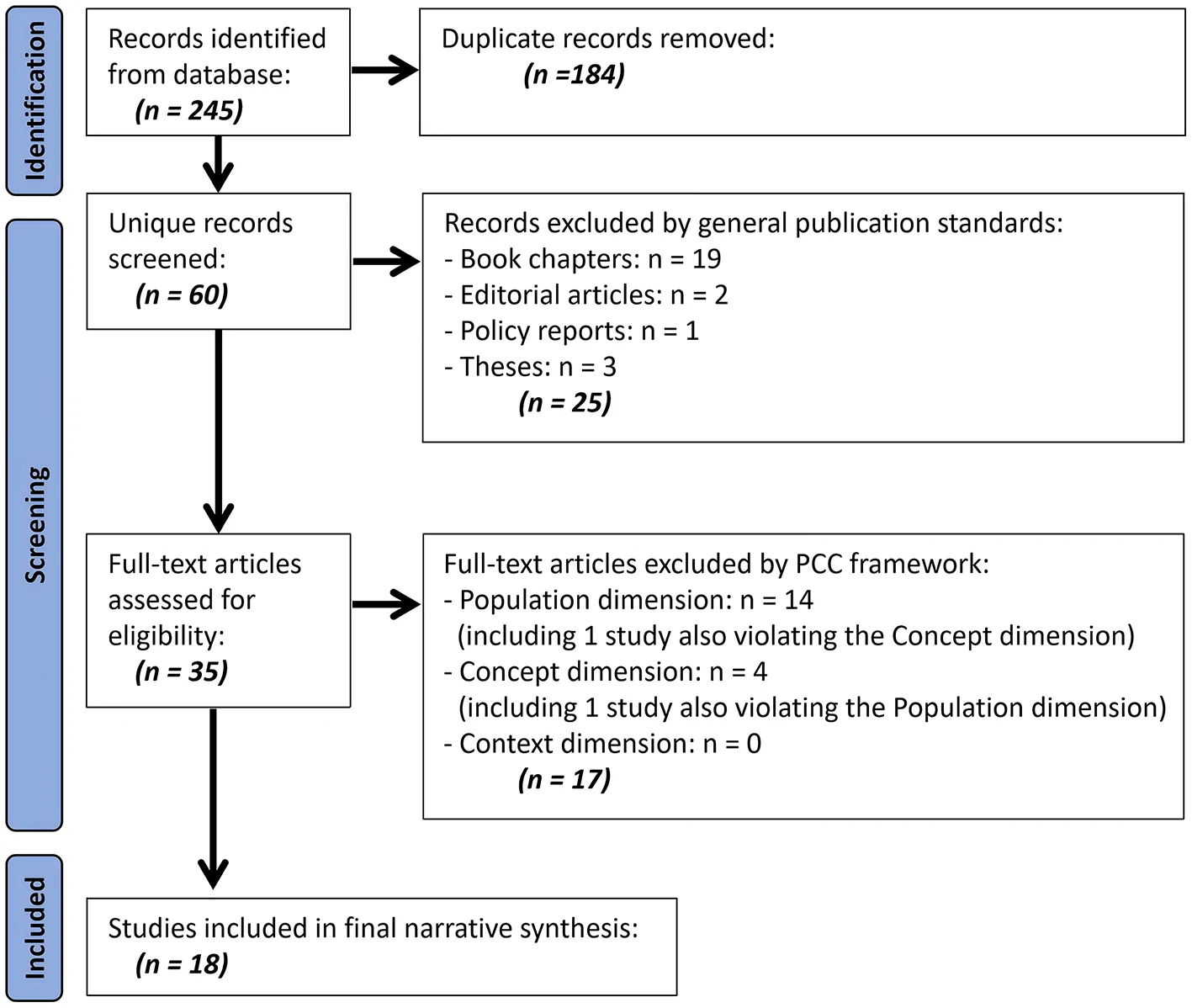
Literature screening and selection process.

**Phase 1: Initial Identification and Deduplication**

The initial Boolean search combined with date range restrictions yielded a total of 245 records across the three databases. After removing cross-database duplicate publications, 60 unique records remained and advanced to the next screening phase.

**Phase 2: General Publication Standards Screening**

The 60 unique records were screened by title and abstract against the general publication standards. A total of 25 records were excluded at this stage. The excluded materials comprised 19 book chapters, 2 editorial articles, 1 policy report, and 3 theses. Following this phase, 35 records remained and proceeded to full-text eligibility assessment.

**Phase 3: PCC Framework Full-Text Screening**

The 35 remaining articles were assessed in full text against the PCC eligibility framework. A total of 17 articles were excluded for failing to meet the PCC criteria. Specifically, 14 studies were excluded on the Population dimension (1 of which also violated the Concept dimension criteria), 4 studies were excluded on the Concept dimension (1 of which also violated the Population dimension criteria), and 0 studies were excluded on the Context dimension.

Following completion of all three screening phases, 18 original peer-reviewed journal articles were included in the final narrative synthesis.

### 2.4 Data extraction and analysis

Data extraction and thematic synthesis proceeded in four sequential stages.

**Stage 1: Evidence extraction**. For each included study, two reviewers independently extracted verbatim excerpts and key findings relevant to the cognitive, affective, and behavioral dimensions and their interactions. Materials were organized into a structured coding database; discrepancies were resolved through iterative discussion until consensus.

**Stage 2: Thematic synthesis (RQ1)**. Evidence within each dimension underwent iterative thematic analysis involving constant comparison and inductive coding. Themes were refined through aggregation and disaggregation until saturation, yielding five interrelated themes per dimension. The aggregated thematic assignment summary for all three dimensions is available in Supplementary Table 1.

**Stage 3: Pathway mapping (RQ2, 1st part)**. Evidence was further coded along three recursive pathways: cognition shaping affect, affect driving behavior, and behavior feeding back to both cognition and affect. Each pathway was analyzed to identify underlying mechanisms and contextual moderators. The aggregated pathway thematic summary is available in Supplementary Table 2.

**Stage 4: Pattern coding (RQ2, 2nd part)**. Analytical units—defined as distinguishable subgroups, generations, or national contexts exhibiting coherent attitudinal configurations—were identified within each study. Each unit was assigned to one of three stable patterns (assimilation, resistance, or structured ambivalence) based on the combination of evidence across all three dimensions and the direction of the feedback cycle. Units with evidence from fewer than three dimensions were marked as partial evidence. Two units documenting explicit pre/post intervention change were coded as transition processes. The full pattern coding table, including 34 analytical units with assigned patterns, verbatim evidence, and assignment rationale, is available in Supplementary Table 3.

**Quality assurance**. Following consensus coding by two primary reviewers, the final thematic framework, pathway mappings, and pattern assignments were reviewed and validated by all co-authors, with remaining ambiguities resolved through group discussion.

## 3 Results

### 3.1 Overview of included studies

Detailed characteristics of each study are provided in Table 3.

**Table 3:** Summary of selected literature.

ID	Author (year)/study country	Study design	Participants	Key findings—RQ1 related	Key findings—RQ2 related
1	Abedini (2025)/USA, Canada, UK	Qualitative study (semi-structured interview & narrative prompts)	25 1.5-generation Iranian immigrant adolescents	This study reveals domain-differentiated language values, affective bonding to Persian, and context-specific code-use patterns.	This study finds bidirectional interplay between identity affect and language behaviors, with host contexts moderating attitudinal patterns.
2	Alsahafi (2026)/Saudi Arabia	Analytical cross-sectional study (questionnaire survey)	179 second- and third-generation Hausa adult residents	This study reveals higher cognitive valuation and affective attachment to Hausa among G2, alongside shrinking intergenerational transmission and literacy.	This study finds positive associations between Hausa proficiency and attitudes, with generational and familial contexts shaping maintenance patterns.
3	Baranova (2026)/Russia (Kalmykia), Germany, Mongolia	Qualitative study (ethnography & linguistic biography interview)	45 Kalmyk speakers across generations (local and diaspora)	This study reveals heterogeneous community attitudes toward non-standard Kalmyk competence and diverse non-family maintenance practices.	This study finds identity attachment drives revitalization behaviors, with historical and diasporic contexts shaping attitudinal patterns.
4	Beaudrie et al. (2019)/USA	Analytical cross-sectional study (questionnaire development & pretest-posttest validation)	301 Spanish heritage language university students	This study reveals biased cognitive evaluations of non-standard Spanish, stigmatized views on code-switching, and varied maintenance intentions.	This study finds that critical language awareness instruction strengthens the link between cognitive re-evaluation and pro-maintenance behavioral intentions.
5	Callesano and Carter (2019)/USA (Miami-Dade County)	Analytical cross-sectional study (matched-guise perceptual experiment)	292 Latinx college students in Miami-Dade County	This study demonstrates that Peninsular Spanish is rated highest for cognitive competence, while warmth perceptions are comparable, and intergenerational transmission intentions vary by dialect.	This study finds that national-origin background labels moderate perceived language maintenance concerns and bilingual code choice patterns.
6	Fatima and Nadeem (2025)/Pakistan	Mixed methods study (semi-structured interview & questionnaire survey)	117 Pakistani individuals from diverse ethnolinguistic heritage groups	This mixed methods study reveals perceived low instrumental status of heritage languages, strong ethnic identity bonding, and generational gaps in use and code-switching.	This study finds strong interplays among identity attachment, family dynamics, and language practices, with societal institutional pressures shaping maintenance patterns.
7	Gao and Liu (2025)/China (Yunnan Province)	Conceptual analysis (mathematical modeling & empirical network validation)	177 Wa and Lahu ethnic minority residents in Ximeng County	This study demonstrates that minority identity strength drives heritage language maintenance behaviors, while language status shapes cognitive legitimacy judgments.	This study identifies three interaction outcomes (extinction, segregation, coexistence) moderated by identity, language status, and social network structures.
8	Gavriilidou and Mitits (2021)/USA (Chicago)	Mixed methods study (online questionnaire & corpus-based content analysis)	54 first-to-third-generation Greek heritage language speakers	This mixed methods study demonstrates domain-differentiated language status, positive affective attachment, and generational decline in heritage language use and literacy.	This study identifies a structured ambivalence pattern, with ethnic identity and family/community institutions moderating attitudinal dynamics.
9	Grgič and Guardiancich (2025)/Italy (Friuli Venezia Giulia region)	Qualitative study (semi-structured interview & documentary analysis)	70 members of the autochthonous Slovenian ethnic minority community	This study reveals perceived exclusive legitimacy of local Slovenian varieties, strong identity-linked linguistic pride, and insular intergenerational in-group maintenance with limited cross-group contact.	This study identifies that community socioeconomic policies and dense social networks moderate the interplay of language evaluations, identity affect, and behaviors to form an insular attitudinal pattern.
10	Inan and Harris (2025)/USA	Conceptual analysis (theoretical opinion paper)	Target population: immigrant heritage language families and ethnolinguistic minority communities	This conceptual analysis identifies cognitive valuation of heritage language value, affective identity bonding, and family-centered maintenance practices with structural constraints amid dominant societal ideologies.	This conceptual analysis argues that educational, policy, community, and digital contexts moderate the interplay of cognitive, affective, and behavioral dynamics to shape maintenance outcomes.
11	Kovacs-Rác and Halupka-Rešetar (2018)/Serbia (Vojvodina region)	Analytical cross-sectional study (questionnaire survey)	290 Hungarian ethnic minority 5th- and 8th-grade pupils	This study demonstrates perceived daily functional legitimacy of local dialects, mostly positive affect alongside reported stigmatization, and context-specific code choice.	This study finds that local identity strengthens dialect attachment and use behaviors, while subtractive minority schooling moderates attitudinal-behavioral patterns.
12	Li (2026)/Italy (Emilia-Romagna region)	Qualitative study (semi-structured interview with multi-perspective design)	18 participants: 6 Chinese migrant families and 6 primary school teachers	This study reveals higher perceived prestige and utility of Putonghua, variable affective attachment to fangyan, and intergenerational shift toward standard heritage use.	This study identifies that standard language ideology, family-school domains, and diasporic contexts moderate the interplay of evaluations, identity, and transmission behaviors.
13	Makarova et al. (2019)/Canada (Saskatchewan)	Analytical cross-sectional study (questionnaire survey & oral proficiency assessment)	30 Russian-speaking immigrant parents and 30 bi/multilingual children aged 5–7	This study demonstrates high perceived instrumental and identity value of Russian, strong positive parental affect, and home-centered heritage language use.	This study finds positive covariation among parental attitudes, language exposure, and child proficiency, with family and community contexts moderating these dynamics.
14	Prada (2019)/USA	Qualitative study (classroom ethnography & semi-structured interview)	17 Latinx Spanish heritage language undergraduate students	This study reveals perceived academic inappropriateness of mixed linguistic practices, pre-existing linguistic shame, and restricted translanguaging behaviors under monoglossic ideologies.	This study finds that translanguaging pedagogy mediates cognitive re-evaluation, affective destigmatization, and normalized flexible language use in educational settings.
15	Shabtaev et al. (2026)/Israel & United States	Analytical cross-sectional study (questionnaire survey)	284 Mountain Jewish heritage speakers across three generations	This study demonstrates higher perceived cognitive prestige and utility for Russian than Juhuri, stronger affective attachment to Juhuri, and variable intergenerational maintenance intentions across groups.	This study finds that ethnic identity strongly predicts tripartite heritage language attitudes, with effects differentially moderated by language, national context, and generation.
16	Siebenhütter (2021)/Thailand	Mixed methods study (questionnaire survey, semi-structured interview, ethnographic observation)	74 young Kui ethnic minority speakers aged 19–25 in northeastern Thailand	This mixed methods study demonstrates lower perceived social prestige of Kui than Thai, positive heritage attachment, and domain-specific code-switching across contexts.	This study finds that ethnic identity, gender dynamics, and national educational institutions moderate the interplay of language evaluations, affect, and use behaviors.
17	Vari and Tamburelli (2023)/Belgium & Luxembourg	Analytical cross-sectional study (auditory Implicit Association Test, questionnaire survey)	127 speakers of endangered Moselle Franconian vernaculars (64 in Belgium, 63 in Luxembourg)	This study demonstrates that closer linguistic distance between standard and vernacular yields more positive implicit evaluations of endangered minority vernacular varieties.	This study finds that standardization mode (endogenous vs. exogenous) moderates positive attitude transfer from standard to vernacular varieties.
18	Yiakoumetti (2022)/United Kingdom	Qualitative study (classroom video observation & semi-structured teacher interview)	4 Mandarin-speaking Chinese ethnic minority heritage language teachers	This study reveals perceived pedagogical utility of dominant English, strong affective heritage attachment to Mandarin, and systematic L1-mediated L2 teaching practices.	This study finds that student language proficiency, parental expectations, and community school contexts moderate teachers' attitudinal and behavioral dynamics.

**Geographic distribution**. The included studies span 14 countries across five continents, with the largest concentration in the United States (6 studies), followed by Italy (2 studies). Single-country studies were conducted in Canada, the United Kingdom, Saudi Arabia, Pakistan, China, Serbia, Thailand, Israel, Belgium, and Luxembourg; three studies adopted cross-national comparative designs (Baranova, 2026; Shabtaev et al., 2026; Vari and Tamburelli, 2023). The heritage languages examined include Iranian Persian, Hausa, Kalmyk, US Spanish, Punjabi/Siraiki, Wa/Lahu, Greek, Slovenian, Hungarian, Putonghua (in the Chinese diaspora), Russian, Juhuri, Kui, and Moselle Franconian.

**Study design**. The evidence base is methodologically balanced: eight studies employed qualitative approaches (semi-structured interviews, ethnography, linguistic biography, classroom observation), eight used quantitative designs (questionnaire surveys, cross-sectional surveys, implicit association tests, perceptual dialectology experiments), one combined mathematical modeling with empirical calibration (Gao and Liu, 2025), and one consisted of a theoretical/conceptual analysis. Sample sizes ranged from 4 (heritage-language teachers) to 301 (university students), with a median of 74 participants; the total number of participants across the 17 studies reporting explicit sample sizes was 2,104.

**Participant characteristics**. Studies recruited diverse participant groups across the lifespan: nine focused primarily on adults (parents, community members, teachers), seven on adolescents and school-age children, and two explicitly included three-generation samples. Generational status constituted a key sampling dimension within migrant and diaspora contexts, with studies targeting first-, second-, and third-generation (G1, G2, G3) heritage speakers, as well as 1.5-generation immigrants. Two studies specifically examined teacher populations (Li, 2026; Yiakoumetti, 2022), while one focused on university-level heritage-language students (Prada, 2019).

### 3.2 RQ1

This section synthesizes the characteristics of ethnic minorities' language attitudes across the three dimensions. The aggregated thematic assignment summary for all three dimensions, listing the themes identified per study, is available in Supplementary Table 1.

#### 3.2.1 Cognitive dimension

Five interrelated themes characterize the cognitive landscape of these attitudes.

First, hierarchized prestige perceptions and internalized standard language ideology structure baseline cognitive evaluations. Ethnic minority speakers widely internalize dominant standard language ideologies, constructing a hierarchical schema of linguistic prestige. Official standard varieties are consistently attributed higher perceived correctness, purity, and social status, whereas heritage dialects and vernacular varieties are categorized as non-standard and linguistically deficient. For example, US Spanish heritage learners position Peninsular Spanish as the authentic, pure norm and judge US Spanish varieties as linguistically incorrect (Beaudrie et al., 2019). Miami Latinx listeners similarly rate Peninsular Spanish speakers significantly higher on competence-related traits than speakers of Colombian and Cuban varieties, linking standard dialects to favorable socioeconomic and occupational outcomes (Callesano and Carter, 2019). Parallel patterns appear in Asian contexts: Punjabi is stereotyped as a non-professional language unfit for high-status work in Pakistan (Fatima and Nadeem, 2025). Code-mixed practices such as Spanglish are further devalued as improper and associated with low educational attainment stereotypes (Prada, 2019). Within heritage communities, judgments of linguistic authenticity further stratify cognitive evaluations, with proficient traditional Kalmyk speakers criticizing and devaluing less proficient speakers (Baranova, 2026).

Second, domain-differentiated functional categorization defines context-specific cognitive judgments of language appropriateness. Heritage dialects are cognitively bounded to informal, in-group contexts such as family and local community, and are primarily defined as oral, intergenerationally transmitted varieties. Greek heritage speakers in the host country, for instance, restrict Greek to home and family settings and prioritize the dominant language for social interaction with friends (Grgič and Guardiancich, 2025). Hungarian minority dialects are similarly categorized as informal spoken language distinct from the standard written variety used in formal education (Kovacs-Rác and Halupka-Rešetar, 2018). In Thailand, Kui is used frequently in homes and local communities but avoided in public settings, as standard Thai is perceived as more practical for cross-community communication (Siebenhütter, 2021). Mainstream educational institutions reinforce this divide by legitimizing standard languages as the normative code for formal learning (Inan and Harris, 2025).

Third, dual-axis value framing distinguishes instrumental utility from identity symbolism in cognitive evaluation. Official standard languages are prioritized for their functional utility in education, employment, and social mobility, whereas heritage languages are primarily valued as markers of ethnic identity and cultural continuity. For Persian heritage speakers in English-dominant contexts, English holds institutional prestige that diminishes the perceived practical value of Persian, yet Persian remains framed as a symbol of cultural authenticity and personal history (Abedini, 2025). Russian-speaking parents in Canada view English as more important for their children's host-country lives, but regard Russian as an essential component of ethnic identity and a potential career asset (Makarova et al., 2019). This dual framing is modulated by socioeconomic position: privileged groups tend to perceive heritage-language bilingualism as a professional asset, while economically disadvantaged groups prioritize dominant-language acquisition for immediate practical benefits (Inan and Harris, 2025). For Hausa speakers in Saudi Arabia, the language is deemed irrelevant to professional goals among those without strong heritage engagement (Alsahafi, 2026).

Fourth, contested categorization and legitimacy surround heritage language status. The classification of heritage varieties as “dialects” rather than independent languages undermines their perceived legitimacy; some Hausa community members, for example, regard Hausa as a dialect, reducing willingness to use it in family settings (Alsahafi, 2026). Social labeling of heritage languages also generates disagreement: in the UK Chinese community, some parents reject framing Chinese as a foreign language for their children, reflecting struggle over the linguistic classification of the heritage variety (Yiakoumetti, 2022). In the Italian Slovenian minority, local varieties are legitimized as the only acceptable codes across settings, while supra-regional standard Slovenian is dismissed as foreign and inadequate (Grgič and Guardiancich, 2025).

Fifth, standardization strategies and ethnic identity moderate cognitive evaluations. Endogenous, linguistically close standards transfer positive prestige to local vernaculars, whereas exogenous, distant standards reinforce standard dominance. In Luxembourg, speakers hold more positive implicit attitudes toward the local vernacular than toward the endogenous standard; in Belgium, by contrast, the exogenous Standard German is preferred over the local vernacular (Vari and Tamburelli, 2023). Ethnic identity also correlates with positive cognitive attitudes toward heritage languages, conditioned by national context and generational change. In Israel, ethnic identity correlates with cognitive attitudes toward both Juhuri and Russian, whereas in the US the correlation holds only for Juhuri and disappears for Russian among second-generation immigrants (Shabtaev et al., 2026). Strong minority identity further frames heritage language as an exclusive in-group code, producing linguistic segregation between groups with differing identity strength (Gao and Liu, 2025).

Taken together, the cognitive dimension of minority language attitudes is structured by hierarchical prestige beliefs, domain-specific functional assignments, and dual value framing, with variation shaped by standardization regimes, ethnic identity, and socioeconomic context. These five themes can be further understood through the lens of three interrelated cognitive belief types: normative beliefs about correctness and appropriateness (encompassing the hierarchized prestige and domain-differentiated functional categorization themes), ontological beliefs about linguistic classification and legitimacy (the contested categorization theme), and social-cognitive schemas about speakers of particular varieties (integrated within the hierarchized prestige theme, where stereotypes about speaker competence and social background co-occur with status evaluations). This three-way distinction helps clarify the distinct cognitive operations underlying the observed patterns while acknowledging that these belief types frequently intersect in empirical practice.

#### 3.2.2 Affective dimension

The affective dimension of language attitudes, encompassing pride, shame, stigma, sense of belonging, and identity attachment, represents a core facet of how ethnic minorities evaluate their heritage dialects against official standard languages. We outline five patterns in affective evaluations of heritage and standard linguistic varieties.

First, affective responses to heritage languages are persistently ambivalent, as in-group pride in linguistic heritage coexists with stigma and anxiety in broader societal contexts. For minority communities, heritage languages typically act as an affective anchor tied to cultural identity, family continuity, and community solidarity. Abedini (2025) notes that Persian fosters personal pride and connection to Iranian heritage among diaspora speakers, while Fatima and Nadeem (2025) report that heritage languages such as Siraiki and Punjabi are viewed as core markers of ethnic belonging in Pakistan. This positive valence is counterbalanced, however, by negative societal stereotypes framing heritage dialects as unprofessional, low-status, or linguistically incorrect. Beaudrie et al. (2019) show that Spanish heritage learners in the US internalize the view that US Spanish varieties are less legitimate than Peninsular Spanish and that Spanish overall carries lower prestige than other foreign languages, generating embedded linguistic insecurity. Similarly, over one third of Hungarian minority pupils in Kovacs-Rác and Halupka-Rešetar (2018) study have experienced mockery for their dialect, and some Kui speakers in Thailand conceal their linguistic background in professional settings for fear of negative consequences (Siebenhütter, 2021). This duality of in-group affirmation and out-group devaluation creates persistent affective tension for minority language speakers.

Second, affective attachment to heritage languages follows a clear generational gradient, though contextual factors may modify this trajectory. In most migrant and minority contexts, affective investment in the heritage language declines across successive generations. First-generation immigrants typically hold the strongest emotional bonds, marked by nostalgia, sense of belonging, and commitment to intergenerational transmission. Gavriilidou and Mitits (2021) find that first-generation Greek heritage speakers in the US express deep affection for Greek and strong identification with Greek culture, while third-generation individuals with limited family cultural investment exhibit affective distancing from the language. A similar pattern appears for Russian heritage speakers (Makarova et al., 2019) and Hausa communities (Alsahafi, 2026), where second-generation speakers retain stronger identity links than the third generation. Notably, this generational decline is not universal. Shabtaev et al. (2026) document that affective attitudes toward the traditional Juhuri language strengthen across generations in the US, as the language gains significance as a unique marker of Mountain Jewish cultural identity. This indicates that the functional distinctiveness of a heritage language within a given societal context can reverse typical generational affective erosion.

Third, a hierarchical affective evaluation typically distinguishes standard from heritage varieties, but this hierarchy is contingent on standardization approaches. Dominant standard language ideologies generally position standard varieties as pure, legitimate, and prestigious, while heritage dialects are framed as non-standard and deficient. Callesano and Carter (2019) demonstrate this hierarchy among Latinx listeners in Miami, who rate Peninsular Spanish higher on competence traits than Colombian and Cuban varieties. Yet this evaluative order is not fixed. When standardization draws on endogenous, locally rooted varieties rather than exogenous external standards, heritage dialects can acquire higher affective valence than the supra-regional standard. Grgič and Guardiancich (2025) find that Slovenian speakers in Italy view local heritage dialects as the only fully acceptable linguistic forms, while supra-regional standard Slovenian is perceived as foreign and undesirable. Vari and Tamburelli (2023) further show that Moselle Franconian speakers in Luxembourg, where an endogenous standard has been developed, hold an implicit positive preference for their vernacular over the standard, whereas Belgian speakers of the same dialect family, who reference exogenous Standard German, favor the standard over the vernacular. Thus, linguistic distance between standard and vernacular is a key predictor of affective valence toward minority dialects.

Fourth, the strength of ethnic identity is positively coupled with affective evaluations of heritage languages. Stronger ethnic commitment and exploration correspond to more positive feelings toward heritage varieties and greater sense of belonging through language. Gao and Liu (2025) show that individuals with strong minority identity exhibit exclusive affective attachment to their own heritage language and tend toward linguistic segregation from other groups, whereas more open identity orientations correspond to greater receptiveness. Shabtaev et al. (2026) add that the magnitude of this identity-affect link varies by language type: it is stronger for ethnically distinct heritage languages such as Juhuri than for more widespread shared languages such as Russian, and dissipates across generations for languages that lose their identity-marking function. Ethnic identity thus operates as a foundational driver of affective investment in heritage linguistic forms.

Finally, affective experiences of language use are highly context-dependent, varying sharply across private in-group and public out-group domains. In family and community settings, heritage languages are associated with intimacy, solidarity, and positive belonging. In public domains such as schools and workplaces, by contrast, heritage language use often triggers shame, embarrassment, or self-consciousness. Li (2026) illustrates this divergence among Chinese migrant children: some express enthusiasm when sharing their heritage language in multicultural classrooms, while others speak only quietly and hesitantly due to linguistic insecurity. Institutional contexts can mediate these effects: inclusive school curricula and community-based heritage language programs provide affirming spaces that strengthen positive affect and sense of belonging (Inan and Harris, 2025), while pedagogical approaches such as translanguaging can shift learners' affective orientation from shame about mixed language practices to pride in their bilingual repertoires (Prada, 2019). Even heritage language teachers exhibit affective discomfort, feeling guilt when they use students' dominant language for instruction, reflecting internalized normative expectations of exclusive target-language use (Yiakoumetti, 2022).

In sum, the affective dimension of minority language attitudes is defined by ambivalence, generational variation, context-dependent expression, and close ties to identity and standardization regimes. These patterns demonstrate that affective evaluations are not fixed traits but dynamic responses shaped by social, institutional, and intergenerational conditions.

For analytical clarity, the affective phenomena documented across these five themes can be further understood through three interrelated forms of affect: (1) discrete emotions—immediate, situation-triggered responses such as pride, shame, anxiety, and embarrassment, most prominently observed in the context-dependent expression theme (Theme 5); (2) evaluative affect—valence-laden judgments about languages and their speakers, such as feelings of preference, aversion, or linguistic insecurity, underlying the hierarchical affective evaluation theme (Theme 3) and the persistent ambivalence theme (Theme 1); and (3) identity-based affect—enduring emotional bonds tied to group membership, such as sense of belonging, attachment, and identification, central to the generational gradient theme (Theme 2) and the ethnic identity coupling theme (Theme 4). While these three forms frequently co-occur and interact in empirical settings, distinguishing them helps clarify the different mechanisms through which affect operates in minority language contexts.

#### 3.2.3 Behavioral dimension

Ethnic minority communities' language practices show consistent yet context-dependent dynamics between heritage dialect engagement and orientations toward dominant official standard languages. Five interrelated behavioral patterns characterize this dimension, spanning intergenerational transmission, contextual code selection, identity-linked practice, institutional influence, and community maintenance efforts.

First, there exists a pronounced generational gradient in language use across diverse geographic and cultural contexts, characterized by intergenerational linguistic divergence and disrupted heritage-language transmission. Older generations generally use the heritage language exclusively or predominantly in domestic and community spaces, and often disapprove of other languages being spoken at home. Younger generations, in contrast, gradually shift toward the official standard language: they frequently engage in code-switching or prefer using the standard language, which correlates with lower heritage-language competence and broken parent-to-child language transmission. This pattern has been observed in multi-ethnic Pakistani families (Fatima and Nadeem, 2025), Greek diaspora communities in the United States (Gavriilidou and Mitits, 2021), Mountain Jewish populations in Israel and the United States (Shabtaev et al., 2026), Kui minority communities in Thailand (Siebenhütter, 2021), Chinese migrant households in Italy (Li, 2026), and Hausa communities in Saudi Arabia (Alsahafi, 2026). Parental behavioral adjustments to standard-language schooling further accelerate this shift. Many caregivers switch to the standard language at home to prepare children for school linguistic requirements, especially when children attend formal education taught entirely in the majority language (Alsahafi, 2026; Siebenhütter, 2021).

Second, minority speakers systematically deploy context-dependent code selection, producing functional linguistic compartmentalization across social domains. Heritage dialects are reserved for private, in-group interactions including family, local community networks, cultural rituals and festivals, while the official standard language dominates public, cross-group, and institutional settings such as education, employment and commerce. Code-switching functions as a critical adaptive behavioral strategy for younger speakers navigating multi-linguistic environments. Domain-specific code partitioning is evident in Thai Kui communities (Siebenhütter, 2021), Chinese migrant families in Italy (Li, 2026), Greek heritage speakers in Chicago (Gavriilidou and Mitits, 2021), and Pakistani minority groups (Fatima and Nadeem, 2025). Notably, teachers report that some children maintain a rigid separation between languages, confining the heritage language to the home domain and avoiding its use in school settings, treating the two languages as contextually distinct (Li, 2026).

Third, the strength of ethnic identity strongly predicts language-choice behavior, while context-bound language ideologies reinforce strict norms favoring local heritage language varieties. Strong minority identity is associated with consistent, exclusive heritage-language use and rejection of outside linguistic forms; weak ethnic identity corresponds to greater acceptance of standard languages and other minority varieties. Agent-based modeling research on the Wa and Lahu minorities in China confirms that strong ethnic identity brings about exclusive heritage-language practice and inter-group linguistic segregation (Gao and Liu, 2025). For Slovenian minorities in Italy, two overlapping ideological systems strengthen this exclusivity. The first-language ideology creates a linguistic boundary between Slovenian minorities and the Italian majority. The language-pride ideology sets another divide between local Slovenian speakers and speakers from the Republic of Slovenia; both ideologies enforce exclusive use of the local regional variant (Grgič and Guardiancich, 2025). Stigma attached to minority linguistic backgrounds also gives rise to deliberate concealment behaviors. Some speakers refrain from using their heritage language in public or professional settings to avoid negative social judgement (Abedini, 2025; Kovacs-Rác and Halupka-Rešetar, 2018; Siebenhütter, 2021).

Fourth, formal institutional policies and practices — including educational standardization, school language policies, and community-run heritage-language education — systematically shape minority speakers' everyday linguistic behavior and implicit language preferences. Different standardization approaches produce contrasting attitudes toward heritage languages versus standard varieties. Exogenous standardization refers to the adoption of an external, geographically distant standard (for instance Standard German among Belgian Moselle Franconian speakers). It fosters an implicit preference for the standard over local vernaculars. Endogenous standardization, by contrast, develops a locally rooted, linguistically proximate standard such as Standard Luxembourgish, and yields implicit preference for the local vernacular (Vari and Tamburelli, 2023). In community-based heritage schools, teachers regularly draw on students' dominant standard language (English) as a teaching support for heritage-language instruction, employing it for teaching delivery, classroom management and socio-emotional purposes (Yiakoumetti, 2022). Empirical evidence shows formal bilingual education and community school programmes are linked to higher heritage-language proficiency and greater linguistic confidence among minority youth (Beaudrie et al., 2019; Inan and Harris, 2025).

Fifth, minority communities adopt multi-layered behavioral measures to preserve heritage dialects, covering individual, family, community and transnational diaspora levels. Family-centered strategies revolve around extended-family participation in daily heritage language interactions, with grandparents serving as especially vital agents for intergenerational language transmission (Fatima and Nadeem, 2025). Community-level initiatives include organized language courses, cultural events, and advocacy for formal heritage-language programmes within schools and universities (Baranova, 2026; Siebenhütter, 2021). Digital resources such as language-learning applications, social-media groups and video content, together with heritage-language radio and television broadcasts, provide accessible informal tools for language maintenance (Inan and Harris, 2025). Diaspora communities further establish transnational linguistic activities and dedicated youth language camps to sustain intergenerational language transmission outside the home country (Baranova, 2026).

Taken together, these behavioral patterns reflect the varied ways in which ethnic minority groups negotiate language use within standard-language-dominant societies, balancing practical integration pressures with efforts to sustain linguistic heritage. The findings highlight that language behaviors are not static or uniform, but shift across generations, contexts, and institutional settings.

### 3.3 RQ2

#### 3.3.1 Cross-dimensional interaction mechanisms

The evidence reveals three recursive pathways: cognition shaping affect, affect driving behavior, and behavior feeding back to both cognition and affect. Together, these pathways describe how evaluative judgments, emotional orientations, and linguistic practices mutually constitute one another. The aggregated pathway thematic summary, documenting the themes identified for each interaction pathway per study, is available in Supplementary Table 2.

##### 3.3.1.1 Cognition shaping affect

Cognitive evaluations of language status and legitimacy serve as core determinants of linguistic affective valence. Linguistic varieties perceived as standard or high-status elicit pride and positive judgements, whereas those classified as non-standard or low-status induce linguistic devaluation, speaker shame, and social marginalization. Local Slovenian speakers in Italy construct their vernacular as a core identity marker, cultivating strong linguistic pride, while dismissing supra-regional standard varieties as alien and foreign (Grgič and Guardiancich, 2025). Vari and Tamburelli (2023) further illustrate that linguistic distance between standard languages and their regional vernaculars modulates the transfer of positive linguistic affect: smaller linguistic distances between standard and local varieties correspond to stronger speaker preference for indigenous linguistic forms. Conversely, Punjabi speakers who perceive their heritage language as non-professional and inadequately supported in formal educational contexts exhibit diminished willingness for language learning and retention (Fatima and Nadeem, 2025).

Shabtaev et al. (2026) provide cross-contextual empirical evidence that a single heritage language can generate diametrically opposed affective responses, contingent on its socially perceived status within the local sociolinguistic ecology. In Israel, Juhuri exists in the sociopolitical shadow of Hebrew, which holds dominant status as a revitalized national lingua franca, resulting in a generational erosion of positive attitudes toward Juhuri. In the United States, by contrast, Juhuri carries unique cultural significance as the exclusive linguistic vehicle preserving Jewish heritage for immigrant communities with no Hebrew exposure. This cultural uniqueness fosters robust positive affective attitudes, particularly among younger 1.5- and second-generation immigrant cohorts. Such cross-contextual discrepancies confirm that linguistic affective valence is not an inherent attribute of a language itself but a context-dependent outcome of cognitive assessments of relative linguistic status.

Parallel attitudinal patterns stem from normative judgements of linguistic correctness. Hausa speakers who categorize their native language as merely “a dialect” demonstrate reluctance toward domestic vernacular use (Alsahafi, 2026). Similarly, heritage language learners who frame code-switching as linguistically inappropriate express explicit disapproval of such practices (Beaudrie et al., 2019). Some Latinx students reported hearing family narratives that portray language mixing as a trait of uneducated speakers, even though they themselves rejected such views (Prada, 2019). Meanwhile, certain parents resisted having their children classified as learners of Chinese-as-a-foreign-language, which reveals conflicts over how to linguistically categorize the heritage language variant (Yiakoumetti, 2022).

Cognitive expectations of linguistic competence and instrumental value further shape speakers' affective responses to language use. Hispanic students who internalize the ideal of “perfect Spanish” experience embarrassment when their linguistic proficiency fails to meet such stringent standards (Prada, 2019). Kalmyk language learners avoid public verbal practice out of anticipatory anxiety over mispronunciation and linguistic inaccuracy (Baranova, 2026). Likewise, Hungarian minority dialect speakers report stigmatization of the dialect in educational settings, with some indicating that anticipated criticism affects their public use of the vernacular (Kovacs-Rác and Halupka-Rešetar, 2018). Iranian migrants construct English as a marker of their “new social identity”, developing stronger emotional attachment to English than to Persian; meanwhile, the perceived social devaluation of Iranian heritage language triggers speaker shame and linguistic disengagement (Abedini, 2025).

From an instrumental perspective, perceived socioeconomic utility motivates adoption of dominant standard languages, while agent-based modeling indicates that strong minority identity corresponds to exclusive heritage-language use and inter-group linguistic segregation (Gao and Liu, 2025). Families that frame bilingualism as a socioeconomic and cultural asset maintain positive linguistic motivation, whereas employment and career concerns lead other households to deprioritize heritage language practice (Inan and Harris, 2025). By comparison, Russian heritage parents in Canada invest in their ancestral language as a symbol of ethnic identity, though limited community resources in some contexts constrain maintenance opportunities (Makarova et al., 2019).

At the individual experiential level, affective language attitudes can be conditioned by lived experiences. Fatima and Nadeem (2025) document a notable case in which Siraiki became a “language of hatred” for a speaker, due to its consistent contextual association with childhood punishment and traumatic experiences. This finding indicates that negative linguistic affect and aversion can be gradually cultivated through repeated pairing of a heritage language with adverse life events.

##### 3.3.1.2 Affect driving behavior

Affective orientations directly shaped linguistic-behavioral choices, with identity attachment and pride functioning as the most consistent drivers of maintenance behavior. Strong ethnocultural attachment motivated intergenerational transmission within families (Alsahafi, 2026; Gavriilidou and Mitits, 2021; Makarova et al., 2019), active participation in community cultural activities (Abedini, 2025; Gavriilidou and Mitits, 2021), advocacy for heritage-language education programs (Alsahafi, 2026; Baranova, 2026), and self-directed language learning among diaspora adults (Baranova, 2026). Grgič and Guardiancich (2025) documented an extreme form: the Slovenian minority in Italy constructed a closed social and economic network enforcing exclusive minority-language use to protect against assimilation. Fatima and Nadeem (2025) provided quantitative support, reporting a strong positive correlation between heritage-language practice and ethnic identity (*r* = 0.628), and Shabtaev et al. (2026) found that individuals with stronger ethnic identity exhibited higher behavioral intentions to sustain heritage-language use.

Negative affective orientations—anxiety, shame, and fear of evaluation—drove restrictive behaviors and public concealment. Concerns about linguistic evaluation and standard-language ideologies motivated some learners to suppress code-switching in formal settings (Beaudrie et al., 2019). Hungarian-minority dialect speakers reported stigmatization of the dialect in educational settings, with some indicating that they avoided public use due to anticipated criticism (Kovacs-Rác and Halupka-Rešetar, 2018). Some Latinx students who faced negative reactions from teachers and peers shifted to predominant English use in school settings (Prada, 2019), and a Kui speaker concealed his ethnic background at work due to fear of negative consequences (Siebenhütter, 2021). A notable finding was the attitude-behavior gap: Kovacs-Rác and Halupka-Rešetar (2018) explicitly showed that positive personal affect toward a dialect did not guarantee public use when fear of external judgment was present, indicating that internal affective dispositions and outward behavioral performance can diverge. This divergence constitutes the behavioral dimension of structured ambivalence: speakers simultaneously hold positive private regard for the heritage variety and anticipatory anxiety about public evaluation, producing a split between private endorsement and public avoidance rather than consistent attitude-behavior alignment.

Instrumental affective evaluations further shaped family-level resource allocation. Families framing bilingualism as a socioeconomic asset actively sought community and digital resources for heritage maintenance (as discussed in Inan and Harris, 2025), whereas employment concerns and perceived societal attitudes generated anxiety that led families to deprioritize heritage languages in domestic language policy (Inan and Harris, 2025; Li, 2026). This pattern indicates that affective orientations are not purely identity expressions but are deeply intertwined with instrumental rationality in shaping behavioral decisions.

At the micro-interactional level, interpersonal affiliative affects shaped code choice in educational settings. Yiakoumetti (2022) documented that teachers in UK Chinese community schools deliberately used students' dominant language (English) to build rapport and assist with personal matters, as students associated their first language with familiarity, informality, and comfort. This finding extends the affect–behavior link beyond macro-level maintenance or shift decisions, demonstrating that affective goals also structure moment-to-moment code selection in instructional interactions.

##### 3.3.1.3 Behavior feeding back to cognition and affect

Sustained language practices reinforced or reshaped cognition and affect through positive and negative feedback cycles. Routine heritage language use and community participation deepened identity cognition and belonging: regular Persian use strengthened intercultural identity (Abedini, 2025); higher Hausa proficiency correlated positively with perceived importance (*r* = 0.32) (Alsahafi, 2026); collective Kalmyk activities intensified feelings of ethnic-linguistic belonging (Baranova, 2026); Greek community engagement increased children's appreciation for the language (Gavriilidou and Mitits, 2021). These findings describe a positive loop in which greater use generates stronger identification and belonging, motivating further use.

Structured educational interventions could actively redirect this feedback. Critical language awareness instruction generated significant positive changes across linguistic prejudice, code-switching attitudes, and maintenance intentions (Beaudrie et al., 2019). Translanguaging pedagogy enabled Latinx students to view their languages differently, producing pride in Spanglish while challenging the assumption of a single ideal standard (Prada, 2019).

Conversely, diminished practice and eroding support undermined positive cognition and affect. Third-generation Greek children whose parents had not invested in cultural maintenance developed stronger American identification, greater distance from Greek traditions, and lower proficiency (Gavriilidou and Mitits, 2021). The collapse of the Slovenian minority's social economy in Italy coincided with declining communicative competence and growing concerns about language attrition (Grgič and Guardiancich, 2025). In Israel, declining intergenerational use of Juhuri and Russian corresponded with attitudinal decline across all three components (Shabtaev et al., 2026), and Kui home use fell from 100% among 58–68-year-olds to 40% among 15–25-year-olds, reinforcing perceptions of endangerment (Siebenhütter, 2021).

Practices of language standardization and institutionalization shape public perceptions and affective evaluations of dialect legitimacy, with language-planning measures exerting direct effects. Vari and Tamburelli (2023) identify standardization strategy type as the primary predictor of dialect attitudes, outweighing sociolinguistic variables including age and gender: endogenous standards with close linguistic distance exert protective effects and reinforce dialect legitimacy perceptions, whereas exogenous standards with large linguistic distance privilege the standard language and undermine dialects. Baranova (2026) further notes that commodification within the language-learning market (private tutoring, commercial courses) retroactively reshapes community-level language attitudes by boosting language visibility.

Contextualized multilingual practices foster flexible cognitive frameworks and open-minded emotional orientations. Li (2026) observes that stable, context-segregated use of Chinese (family/heritage) and Italian (school-communication) reinforces a functional division in which the heritage variety is tied to family and cultural continuity while the majority language serves school and cross-group communication. Siebenhütter (2021) reports that young Kui-speakers' situational code-switching between Kui and Thai cultivates open multilingual attitudes and flexible language orientations. Yiakoumetti (2022) illustrates that teachers' systematic incorporation of students' L1 into heritage-language teaching strengthens perceptions of L1 as an effective resource for heritage-language learning.

#### 3.3.2 Three stable attitudinal patterns

The interplay of cognitive, affective, and behavioral dimensions generates distinguishable attitudinal configurations among heritage language speakers. Across the 18 reviewed studies, 34 analytical units were identified, each assigned to one of three stable patterns—assimilation, resistance, and structured ambivalence—with two additional units documenting transition processes.

##### 3.3.2.1 Assimilation pattern

The assimilation pattern is defined by consistent cognitive devaluation of the heritage variety, affective distancing from it, and behavioral contraction of its use domains. Six analytical units exhibited this configuration.

In Abedini (2025), participants who framed English as the carrier of a “new personality” expressed zero interest in Iranian heritage and reported declining engagement with Persian traditions since migration. Alsahafi (2026) documented a subgroup that framed Hausa as non-competitive with English and Arabic, describing language shift as a “natural consequence” rather than a problem. Gavriilidou and Mitits (2021) found that third-generation Greek heritage speakers whose parents had not invested in cultural maintenance identified primarily as American, distanced themselves from Greek customs, and showed low Greek proficiency. Li (2026) reported that four of six Chinese migrant families in Italy adopted exclusive Putonghua use at home, dismissing fangyan as geographically limited, useless outside its region, and potentially interfering with standard pronunciation. Shabtaev et al. (2026) provided cross-contextual evidence: in Israel, attitudes toward both Juhuri and Russian declined across all three attitudinal components from the first to the second generation, overshadowed by Hebrew's dominant status; in the United States, Russian became decoupled from Mountain Jewish identity across generations, with no significant correlation between ethnic identity and Russian attitudes among second-generation participants.

The feedback mechanism in this pattern is uniformly negative: reduced use reinforces perceptions of low importance, which in turn drives further affective distancing and behavioral contraction.

##### 3.3.2.2 Resistance pattern

The resistance pattern is defined by consistent cognitive revaluation of the heritage language as identity-bearing, strong positive affective investment, and active behavioral maintenance or expansion. Eleven analytical units fell under this pattern.

Abedini (2025) documented participants framing Persian as an emotional anchor (“a thread that ties us together at home”) and deploying it across family interactions, social-media live streams, and friend conversations. Alsahafi (2026) found second-generation Hausa adults regarded Hausa as internationally functional—notably for Hajj and Umrah-related communication—and voiced concern about its decline. Baranova (2026) distinguished two resistant profiles: older new-speakers relearning Kalmyk through self-study (e.g., reading Kalmyk-language newspapers); and diaspora Kalmyks across Canada, the United States, Germany and Mongolia, whose emigration contexts fostered innovative maintenance strategies. Fatima and Nadeem (2025) observed older generations within Pakistani multigenerational households prioritized heritage over dominant languages through storytelling and ritual transmission. Gao and Liu (2025) reported exclusive Wa-language use among respondents with exceptionally high minority-identity scores (130/140). Gavriilidou and Mitits (2021) noted first-generation Greek speakers retained Greek as their dominant language and sustained engagement within Chicago's Greek community. Grgič and Guardiancich (2025) outlined how Slovenian minorities in Italy (1945–1991) established minority-language-exclusive settings to counter assimilation pressures. Through a conceptual analysis, Inan and Harris (2025) observed that families viewing bilingualism as a socioeconomic advantage leverage community-led and digital resources to preserve their heritage language. Makarova et al. (2019) found that Saskatchewan-based Russian-speaking parents demonstrated a strong commitment to heritage maintenance, with 100 % of respondents endorsing the importance of Russian for people of Russian-speaking ancestry. Shabtaev et al. (2026) demonstrated that Juhuri in the United States, seen as the unique vessel of Mountain Jewish heritage, exhibited no intergenerational attitudinal erosion, with affective orientations even intensifying among the second generation.

A positive feedback cycle underpins this pattern: greater language use bolsters group identification, consolidates affective belonging, and drives continued maintenance efforts.

##### 3.3.2.3 Structured ambivalence pattern

The structured ambivalence pattern involves contradictory cognitive evaluations or an attitude-behavior gap, with behavioral domain differentiation as a common manifestation. Ten analytical units exhibited this configuration, four of which were classified as the domain-differentiated subtype.

Abedini (2025) documented both a domain-differentiated subtype—participants with an intercultural identity who maintained Persian traditions while also celebrating Christmas and Thanksgiving—and a classic ambivalent case in which a participant experienced simultaneous pride and shame toward Iranian culture due to negative stereotypes. Alsahafi (2026) found that third-generation Hausa adults endorsed intergenerational transmission in principle yet rated Hausa's importance and international utility lower than the second generation, with some expressing preference for teaching dominant languages for practical advantages. Baranova (2026) reported that young Kalmyks attached high value to knowing Kalmyk yet had prioritized Russian during schooling, expressing regret and shame about their limited proficiency. Fatima and Nadeem (2025) found that younger generations recognized heritage languages as identity markers while simultaneously prioritizing English for professional advantages, with code-switching emerging as a significant intergenerational practice. Inan and Harris (2025), in a conceptual analysis, argued that families facing economic pressures may desire heritage preservation yet deprioritize it due to employment concerns. Kovacs-Rác and Halupka-Rešetar (2018) provided the canonical attitude-behavior gap: 77.24% of Hungarian minority pupils considered the dialect attractive, while 10.33% endorsed the statement “I like it but I do not use it often because I think that I might be judged,” indicating a divergence between positive private regard and public avoidance.

The domain-differentiated subtype was observed in Li (2026), where one family maintained fangyan at home for intergenerational continuity while valuing Putonghua for future opportunities; in Siebenhütter (2021), where young Kui speakers used Kui at home and in the community while shifting to Thai at school and with outsiders, maintaining Kui primarily in private and in-group domains; and in Yiakoumetti (2022), where teachers in UK Chinese community schools used Mandarin for formal instruction and English for explanation, rapport-building, and classroom management.

In all ambivalent configurations, the contradictory state is maintained rather than resolved, managed through domain separation or behavioral concealment rather than eliminated.

##### 3.3.2.4 Transition processes

Two studies documented deliberate interventions that produced measurable attitudinal change across dimensions, rather than stable patterns. Beaudrie et al. (2019) found that a semester-long critical language awareness course shifted Spanish heritage students from pre-intervention patterns of codeswitching suppression in formal settings (e.g., “I would not codeswitch in front of my teachers because they may think I am less intelligent”) toward significantly increased maintenance intentions and reduced linguistic prejudice across all three measured subscales. Prada (2019) documented that translanguaging pedagogy transformed Latinx heritage students from pre-intervention shame and predominant English use in academic settings toward post-intervention pride in Spanglish and endorsement of its classroom legitimacy. Both cases demonstrate that structured educational interventions can redirect the feedback cycle, shifting configurations from ambivalence toward resistance.

## 4 Discussion

### 4.1 Findings: tripartite structure, interaction pathways, and stable patterns

This narrative review synthesizes 18 studies to examine how ethnic minorities' language attitudes toward heritage dialects and official standard languages are structured across cognitive, affective, and behavioral dimensions, and how these dimensions interact to form stable attitudinal configurations. The findings can be organized along three interrelated levels: dimensional characteristics, interaction pathways, and stable patterns, as depicted in Figure 2.

**Figure 2:**
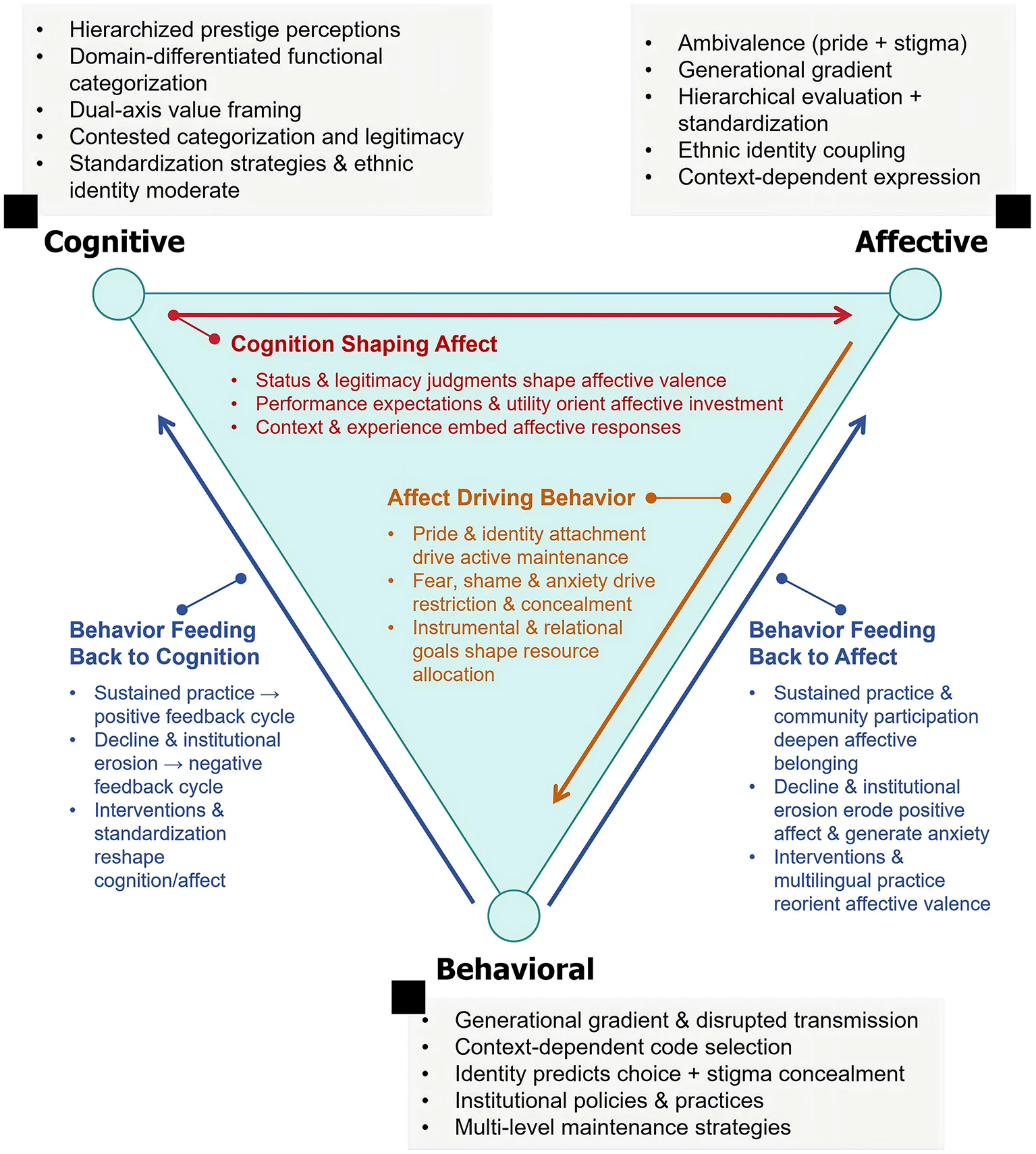
Cognitive–affective–behavioral dimensions and their interaction pathways.

At the dimensional level, all three dimensions exhibit systematic variation across contexts. The cognitive dimension is structured by hierarchized prestige perceptions, domain-differentiated functional categorization, and dual-axis value framing that distinguishes instrumental utility from identity symbolism. The affective dimension is defined by persistent ambivalence, a generational gradient, hierarchical evaluation contingent on standardization approach, identity-coupled valence, and context-dependent expression. The behavioral dimension is characterized by a generational gradient in use, context-dependent code selection, identity-predicted choice accompanied by stigma-driven concealment, institutionally shaped practices, and multi-level maintenance strategies. A cross-dimensional commonality is that all three are modulated by standardization strategy type and ethnic identity strength, suggesting these two factors operate as higher-order regulators of attitude formation.

At the pathway level, the three dimensions do not operate independently but interact through four recursive pathways. Cognition shapes affect: evaluations of legitimacy, status, and utility directly determine emotional orientation toward heritage varieties. Affect drives behavior: identity attachment and pride motivate maintenance, while shame and fear drive restriction and concealment. Behavior feeds back to cognition: sustained use reinforces perceived importance and legitimacy, while decline generates pessimistic assessments of language viability. Behavior also feeds back to affect: regular use deepens belonging and attachment, whereas attrition erodes positive emotional investment. Together, these pathways form a recursive cycle rather than a linear causal chain, with behavior operating as both an outcome and a determinant of cognitive and affective orientations. These dimensional interactions yield three recurring stable configurations, as summarized in Figure 3.

**Figure 3:**
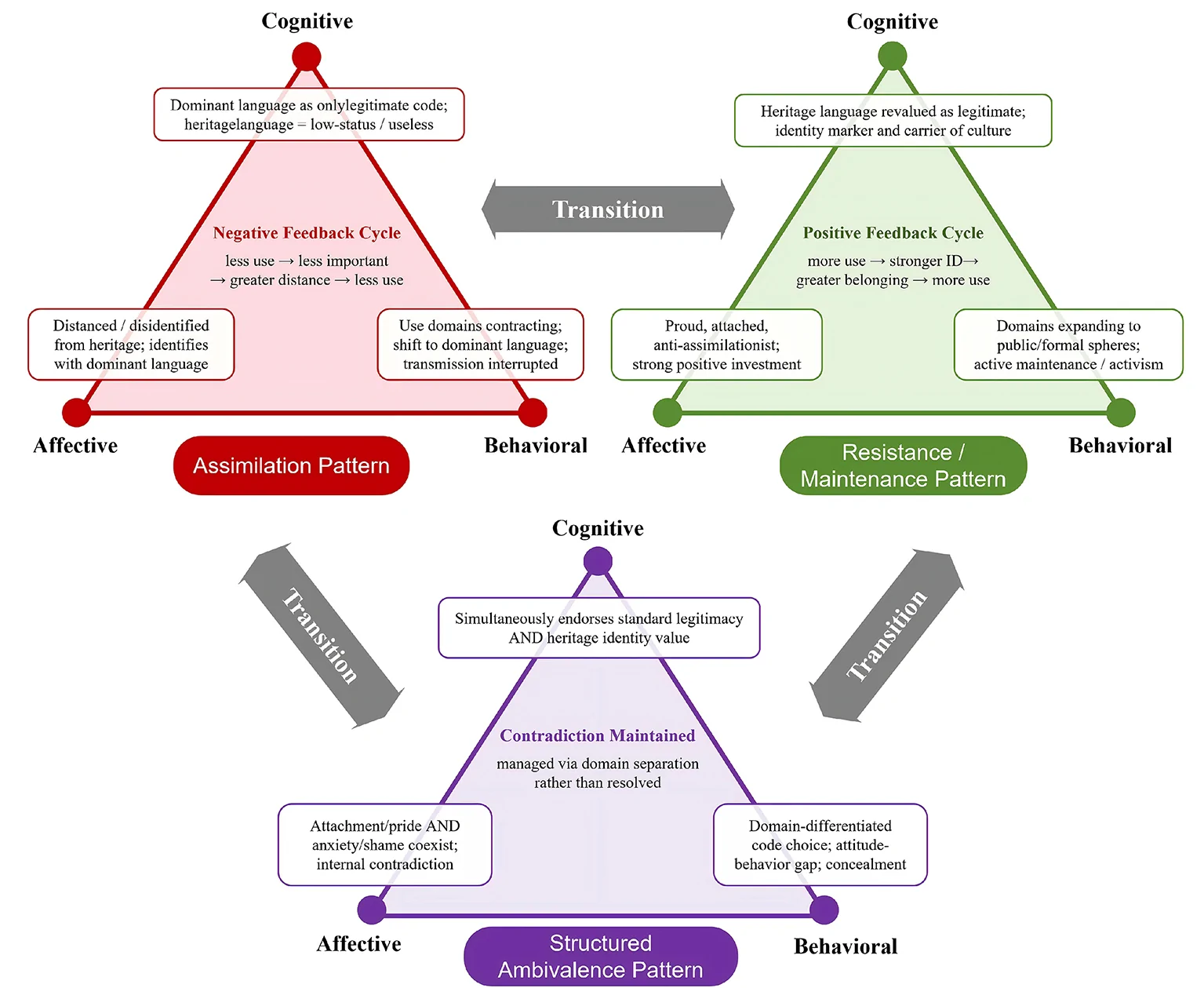
Three stable attitudinal patterns—assimilation, resistance, and structured ambivalence.

The assimilation pattern is driven by consistent cognitive devaluation, affective distancing, and behavioral contraction, producing a negative feedback cycle in which reduced use reinforces perceptions of low importance, which in turn drives further distancing. The resistance pattern is driven by consistent cognitive revaluation, strong affective attachment, and active behavioral maintenance or expansion, producing a positive feedback cycle in which increased use strengthens identification and motivates further use. The structured ambivalence pattern is the most frequently documented configuration, arising when contradictory evaluations or an attitude-behavior gap are managed through domain differentiation rather than resolved. In this pattern, heritage language use is maintained in private or in-group domains while the standard language dominates public or formal spheres, and the contradiction is stabilized rather than eliminated. Two intervention studies (Beaudrie et al., 2019; Prada, 2019) further demonstrate that these configurations are not immutable: critical language awareness instruction and translanguaging pedagogy can redirect ambivalent configurations toward resistance, indicating that educational interventions constitute an external mechanism for breaking existing feedback cycles.

Pattern distributions across studies indicate that contextual factors—such as the existence of a dominant co-ethnic language, the type of standardization strategy, and generational status—determine the resulting configuration, whereas the interplay among cognition, affect and behavior serves as the mechanism for maintaining or altering such stability.

### 4.2 Dialogue with existing literature

#### 4.2.1 Alignment with the tri-component model of attitudes

The three-dimensional framework adopted in this review is consistent with the classic tri-component model of attitudes, which holds that attitudes consist of cognitive, affective, and behavioral components (Ostrom, 1969). The findings verify that all three components exist in ethnic minorities' assessments of heritage and standard languages, with each component showing distinct features. Nevertheless, this review expands the traditional model in two ways. First, instead of viewing the three components as following a linear sequence (cognition → affect → behavior), the evidence points to recursive feedback loops, notably the bidirectional effects of behavior on both cognition and affect. Second, this review shows that the overall attitudinal pattern is determined by the specific configuration of the three components, rather than by any single component in isolation. Structured ambivalence arises as a stable configuration when these components conflict with one another instead of being congruent.

#### 4.2.2 Position within the evolution of language attitude research

Scholarship on language attitudes has progressed through three theoretically successive stages: unidimensional prestige measurement, multi-component attitude assessment, and, most recently, attempts at dimensional integration. This review directly responds to the limitation identified in the third stage, namely that dimensional integration remains scarce despite widespread recognition that attitudes are multi-faceted. By systematically mapping the interactions among cognitive, affective, and behavioral dimensions across 18 studies, this review provides an empirically grounded framework for understanding how the dimensions combine to form coherent attitudinal patterns. The finding that structured ambivalence is the most frequently documented configuration further suggests that previous research, which often assumed a bipolar continuum between maintenance and shift, may have oversimplified the attitudinal landscape by failing to capture stable intermediate states.

#### 4.2.3 Connection to language ideology and standardization research

The cognitive dimension's hierarchized prestige perceptions correspond closely to the concept of dominant language ideologies, whereby minority speakers internalize the legitimacy narrative of the standard language and devalue their own varieties (Kroskrity, 1997). This review extends this literature by identifying standardization strategy type as a critical moderator of ideological internalization. Specifically, endogenous, linguistically close standards transfer positive prestige to local vernaculars, whereas exogenous, distant standards reinforce standard dominance. This finding is exemplified by the contrast between Luxembourg, where speakers hold implicit positive attitudes toward the local vernacular, and Belgium, where the exogenous Standard German is preferred over the same dialect family (Vari and Tamburelli, 2023). The implication is that language ideology is not a monolithic force but is mediated by the structural relationship between the standard and the vernacular.

#### 4.2.4 Reconsidering language shift and maintenance theory

The assimilation and resistance patterns identified in this review correspond to the classical constructs of language shift and language maintenance, respectively. However, the structured ambivalence pattern challenges the assumption implicit in much shift-maintenance research that intermediate states are transitional and inherently unstable. The evidence shows that domain-differentiated code choice can sustain contradictory attitudes over extended periods, with the private-public divide functioning as a psychological buffer that prevents cognitive dissonance from motivating change. This finding suggests that domain differentiation can function as a de facto diglossic arrangement in migrant and minority contexts where the division is not institutionally mandated but emerges through everyday practice. The review also identifies contextual factors that determine whether a given group moves toward assimilation or resistance, including the presence of a competing co-ethnic language and the functional distinctiveness of the heritage variety within the national linguistic ecology.

#### 4.2.5 Cross-contextual comparisons and unexpected findings

Two cross-contextual findings warrant particular attention (Shabtaev et al., 2026). First, the generational decline in affective attachment observed in most migrant contexts is not universal: Juhuri speakers in the United States show increasing affective investment across generations, as the language acquires significance as the sole carrier of Mountain Jewish cultural identity. This reversal demonstrates that the functional distinctiveness of a heritage language can counteract typical generational attrition. Second, the same heritage language can receive diametrically opposed evaluations across national contexts: Juhuri is devalued in Israel, where Hebrew's dominant status overshadows it, but positively evaluated in the United States, where it serves as a unique identity marker. These comparisons underscore that language attitudes are not intrinsic properties of languages but are dynamically constructed within specific linguistic ecologies.

### 4.3 Theoretical explanation and research gaps

#### 4.3.1 Mechanisms underlying dimensional interaction

The observed interaction patterns can be accounted for by several interrelated mechanisms. Cognition shapes affect because language status evaluations map directly onto self-worth assessments: when a heritage language is judged as low-status, speakers who use it may experience devaluation of their own social identity, generating shame and anxiety. Conversely, when the heritage language is revalued as legitimate, speakers derive pride from its use. Affect drives behavior because emotional orientations function as motivational systems: attachment and belonging generate willingness to invest time and resources in maintenance, while fear and shame activate avoidance strategies designed to minimize social judgment.

Behavior feeds back to cognition because language use constitutes experiential evidence that either confirms or disconfirms prior beliefs about a language's importance and viability. Behavior feeds back to affect because regular use in meaningful contexts generates embodied emotional associations—intimacy, solidarity, belonging—whereas prolonged disuse weakens these associations.

The stability of structured ambivalence can be explained by the psychological function of domain differentiation (Ashforth et al., 2014; Gent et al., 2024). By restricting heritage language use to private or in-group contexts and the standard language to public or formal domains, speakers avoid situations that would force a choice between contradictory evaluations. This separation prevents the cognitive dissonance that might otherwise motivate a shift toward either assimilation or resistance, allowing the contradictory state to persist indefinitely. The implication is that structured ambivalence is not a transitional phase but a self-sustaining configuration maintained through behavioral compartmentalization.

#### 4.3.2 Research gaps

##### 4.3.2.1 RQ1: Dimensional characteristics

First, evidence across the three dimensions is uneven, with behavioral data consistently underrepresented. Several included studies measured only cognitive and affective orientations through questionnaires or implicit association tests, without collecting data on actual language use practices. This imbalance limits the completeness of dimensional characterization and prevents full pattern assignment in cases where behavioral evidence is absent.

Second, systematic comparison of the relative weight and interaction intensity of the three dimensions within the same population remains scarce. Most studies examined one dimension in depth or reported dimensions separately, without investigating how cognitive, affective, and behavioral orientations combine in specific individuals or groups, or which dimension exerts the strongest influence on overall attitude formation.

Third, the generalizability of dimensional characteristics beyond Western migrant contexts requires systematic examination. The majority of included studies were conducted in the United States and Europe, with fewer from Asian and African contexts. Whether the five characteristics identified for each dimension manifest identically in collectivist cultural settings, where group identity may carry different weight, remains an open question.

Fourth, the differential impact of standardization strategies on each dimension is not well understood. Existing research has established that endogenous vs. exogenous standards affect overall attitudes, but few studies have disaggregated these effects to determine whether standardization type primarily alters cognitive evaluations of legitimacy, affective valence, or behavioral patterns of use.

Fifth, the divergence between implicit and explicit attitudes remains underexplored in minority language contexts. While one included study employed an implicit association test to measure subconscious preferences for local vernaculars vs. standard varieties (Vari and Tamburelli, 2023), most included studies relied exclusively on explicit self-report measures such as questionnaires and interviews. The relationship between implicit and explicit attitudes—including their potential divergence, the conditions under which they align or conflict, and their differential predictive power for actual language use behavior—has not been systematically examined in heritage language settings. This gap is particularly consequential given the attitude–behavior gap observed in the structured ambivalence pattern, where speakers may hold positive implicit associations with their heritage language while simultaneously reporting explicit anxiety about public use, producing a split between private endorsement and public avoidance.

##### 4.3.2.2 RQ2: interaction pathways and patterns

First, the two feedback pathways from behavior to cognition and behavior to affect have not been adequately distinguished. Existing literature typically refers to “behavior influencing attitudes” as a single process, but the mechanisms, timelines, and intervention targets may differ substantially between behavioral effects on perceived importance (cognitive) and behavioral effects on sense of belonging (affective). Disentangling these pathways would provide a more precise understanding of attitude change mechanisms.

Second, longitudinal evidence of pattern transition is scarce. Of the 18 included studies, only two employed pre-test/post-test intervention designs that documented measurable change across dimensions. The dynamic processes through which configurations shift—including critical turning points, the sequence of dimensional change, and the long-term stability of intervention effects—remain largely undocumented.

Third, the long-term stability of structured ambivalence lacks longitudinal verification. The claim that domain differentiation maintains contradictory states over time is based primarily on cross-sectional data. Whether ambivalent configurations inevitably resolve toward either assimilation or resistance over generational time, or whether they can persist across multiple generations, has not been systematically tracked.

Fourth, the contextual determinants of pattern distribution lack systematic cross-contextual comparison. Factors such as the presence of a dominant co-ethnic language, standardization strategy type, generational position, and socioeconomic status have been identified in individual studies, but no study has systematically compared their relative power in predicting which attitudinal pattern emerges across diverse linguistic ecologies.

Fifth, methodological limitations prevent complete pattern assignment in a notable subset of cases. Five analytical units could not be assigned a full pattern due to missing dimensional evidence. Callesano and Carter (2019), a perceptual dialectology study, lacked participants' own behavioral data. Gao and Liu (2025) lacked direct cognitive and behavioral evidence for Lahu individuals with weak minority identity. Grgič and Guardiancich (2025) documented behavioral decline after the collapse of the social economy but found cognitive and affective attitudes still dominated by pride, preventing full pattern assignment. Vari and Tamburelli (2023), using an implicit association test, provided cognitive and affective evidence but lacked behavioral data, though the Luxembourg endogenous-standard configuration pointed toward resistance and the Belgian exogenous-standard configuration toward assimilation. These cases underscore that complete pattern assignment requires evidence across all three dimensions; attitudinal measurement alone is insufficient and reflects a systematic methodological gap in the field.

### 4.4 Theoretical and practical implications

#### 4.4.1 Theoretical implications

This review makes three primary theoretical contributions. First, it proposes an integrated “three dimensions–four pathways–three patterns” framework that consolidates fragmented research on minority language attitudes into a coherent analytical model. This framework addresses the identified gap in dimensional integration by specifying not only the characteristics of each dimension but also the mechanisms through which dimensions interact and the configurations that result from their combination.

Second, the review advances understanding of structured ambivalence as a stable attitudinal configuration rather than a transitional state. By identifying domain differentiation as the psychological mechanism that maintains contradictory evaluations, the review challenges the bipolar assumption implicit in much shift-maintenance research and expands the theoretical landscape of language attitude studies to include stable intermediate states.

Third, the distinction between two behavior-feedback pathways—to cognition and to affect—provides a more fine-grained mechanism for attitude change theory. This distinction suggests that interventions targeting behavioral change may operate through different routes depending on whether they aim to alter perceived importance or emotional belonging, with implications for the design and timing of attitude-change interventions.

#### 4.4.2 Practical implications

For language policymakers, the finding that standardization (Vari and Tamburelli, 2023) strategy type moderates attitudes toward vernaculars suggests that endogenous, linguistically close standards are more effective than exogenous standards for supporting dialect vitality. Policies that impose distant external standards may inadvertently reinforce standard dominance and accelerate heritage language decline, whereas policies that develop standards from local varieties can transfer prestige to the vernacular.

For educators, the evidence that (Beaudrie et al., 2019; Prada, 2019) critical language awareness and translanguaging pedagogy can redirect ambivalent attitudes toward resistance supports the integration of sociolinguistic awareness training into heritage language teacher education. Teachers who understand the multi-dimensional nature of language attitudes are better equipped to design classroom environments that validate students' linguistic repertoires rather than reinforcing shame about mixed or non-standard practices.

For community-based organizations engaged in heritage language maintenance, the identification of grandparents as key agents of intergenerational transmission, and of family language policy as a critical intervention point, suggests that maintenance efforts should target family-level practices rather than relying solely on school-based programs (Fatima and Nadeem, 2025; Makarova et al., 2019). Community-based activities that create meaningful contexts for heritage language use across generations may be particularly effective for strengthening the behavioral feedback loop that sustains positive attitudes.

For minority language speakers themselves, the recognition that structured ambivalence and attitude-behavior gaps are common, adaptive responses rather than signs of insufficient commitment may reduce self-blame among individuals who value their heritage language but use it primarily in private domains. Understanding the structural factors that shape these patterns can help speakers distinguish between personal choice and externally constrained practice.

### 4.5 Limitations and future directions

#### 4.5.1 Limitations

Several limitations of this review should be acknowledged. First, the literature search was conducted across three databases—Web of Science, Scopus, and Google Scholar—and may have omitted relevant studies indexed in discipline-specific databases such as ERIC or the Linguistic List. Second, only studies published in English were included, which may have underrepresented research from non-English-speaking contexts and potentially skewed the geographic distribution of included studies. Third, the review was restricted to studies published between 2015 and 2026, excluding earlier foundational work that was cited for theoretical context but not subjected to the same analytical framework. Fourth, as a narrative review, this study did not conduct formal risk-of-bias assessment or evidence-grading, and the conclusions may reflect variation in the methodological quality of included studies. Fifth, the included studies were geographically concentrated, with six of eighteen conducted in the United States and single studies in most other countries, limiting the statistical power of cross-contextual comparisons and the generalizability of the proposed framework.

#### 4.5.2 Future directions

The research gaps identified in Section 4.3.2 point to multiple avenues for future research.

Regarding dimensional characteristics (RQ1), future work ought to develop integrated measurement instruments that simultaneously cover cognitive, affective, and behavioral dimensions. Such tools should incorporate objective or logged behavioral data, instead of depending solely on self-reported attitudes. Comparative studies are needed to assess the relative importance of each dimension within the same sample, and verify whether these dimensional characteristics hold true for non-Western collectivist settings. Researchers should further decompose the impacts of standardization strategies, to clarify whether endogenous and exogenous standards mainly shape cognitive, affective or behavioral orientations. Additional research may systematically compare implicit and explicit attitudinal measures among the same population. By combining techniques like implicit association tests with questionnaire-derived explicit measurements, scholars can analyze their discrepancies and distinct predictive validity for behavioral outcomes. This is especially critical for unpacking the attitude-behavior gap under structured ambivalence, a scenario in which implicit preferences and explicit evaluations may pull in opposite directions.

With respect to interaction pathways and patterns (RQ2), longitudinal studies should track attitudinal change over periods of 1 to 3 years to document the dynamic processes of pattern transition, including critical turning points and the sequence of dimensional change. Experimental or intervention studies should separately manipulate the behavior-to-cognition and behavior-to-affect pathways to compare their mechanisms and relative effectiveness as intervention targets. Long-term tracking of ambivalent configurations is needed to determine whether domain-differentiated patterns persist across generations or inevitably resolve toward assimilation or resistance. Finally, systematic cross-contextual comparisons should identify which contextual factors—co-ethnic language presence, standardization type, generational position, socioeconomic status—most strongly predict the emergence of specific attitudinal patterns.

Methodologically, the field would benefit from establishing minimum evidence requirements for pattern assignment, ensuring that future studies collect data across all three dimensions rather than relying on attitudinal measurement alone. Such standards would improve the comparability of findings across studies and enable more robust testing of the three-pattern framework proposed in this review.

### 4.6 Conclusion

This review makes three key contributions to the study of minority language attitudes. First, it provides a systematic characterization of the cognitive, affective, and behavioral dimensions, identifying five recurrent themes within each across 18 studies spanning multiple continents and sociolinguistic contexts. Second, and most importantly, it identifies three stable attitudinal patterns—assimilation, resistance, and structured ambivalence—and demonstrates that structured ambivalence is not a transitional state but a self-sustaining configuration maintained through domain-differentiated code choice, thereby challenging the bipolar continuum assumed in much language shift and maintenance research. Third, it distinguishes two behavior-feedback pathways (to cognition and to affect), refining the mechanism of attitude change.

Taken together, we argue that minority language attitudes operate as a dynamic, recursive system rather than a static evaluation: beliefs, emotions, and practices continuously co-evolve through the four interaction pathways, and their specific configurations—rather than any single dimension in isolation—determine whether a community moves toward assimilation, resistance, or stable ambivalence.

Practically, endogenous standardization strategies, critical language awareness pedagogy, and family-level interventions targeting intergenerational transmission show particular promise for supporting heritage language vitality. Several limitations—the underrepresentation of behavioral data, the scarcity of longitudinal evidence, and the concentration of research in Western contexts—should guide future inquiry. Understanding minority language attitudes, therefore, requires moving beyond static, single-dimension evaluations toward a dynamic, multi-level model of how beliefs, emotions, and practices co-evolve under conditions of structural inequality and the ongoing struggle for linguistic recognition.
