# Supplementary material for: Ethnic minorities' language attitudes toward heritage dialects and official standard languages: a narrative review across cognitive, affective, and behavioral dimensions
Source: Front Psychol. 2026 Sep 28;17:1818757. doi: 10.3389/fpsyg.2026.1818757 (PMC13655717; doi:10.3389/fpsyg.2026.1818757)
Supplement: Supplementary file 1 [file Table_1.pdf]

Supplementary Table 1
RQ1 – Dimensional Thematic Assignment Summary

Purpose.

This table supports the narrative review titled *Ethnic Minorities' Language Attitudes toward Heritage Dialects and Official Standard Languages: A Narrative Review across Cognitive, Affective, and Behavioral Dimensions.* It presents the aggregated thematic assignments for Research Question 1 (RQ1): *What are the characteristics of ethnic minorities' language attitudes across the cognitive, affective, and behavioral dimensions?*

Structure.

This document contains three sub-tables, one per attitudinal dimension:

1) **Table A.** Cognitive Dimension (pp. 2-6)
2) **Table B.** Affective Dimension (pp. 7-15)
3) **Table C.** Behavioral Dimension (pp. 16-32)

How to read.

Within each sub-table, every included study is listed with the dimension-specific themes identified from its verbatim excerpts, accompanied by supporting quotations with page numbers. Themes were identified at the study level through inductive coding of extracted evidence; these study-level themes were then aggregated across all 18 studies through iterative constant comparison, yielding five overarching themes per dimension, as reported in Section 3.2 of the main text.

Table A. Cognitive Dimension

No.	Author (Year)	Original Excerpt – Cognitive Dimension	Thematic Summary – Cognitive Dimension
1	Abedini (2025)	1. English, as the language of education, media, and wider communication, holds institutional and social prestige that reinforces its dominance. This dominance often diminishes the perceived value of the HL, leading to reduced use and a gradual erosion of cultural connection.(p. 14); 2. This identity positioning enabled them to value Persian not just as a familial language but as a symbol of cultural authenticity and personal history.(p. 13)	A(1). English as the dominant official standard language is attributed higher institutional and social prestige, which reduces the perceived cultural value of Persian heritage language; B(2). Persian heritage language is cognitively framed as both a familial communication medium and a symbolic marker of cultural authenticity and personal historical continuity.
2	Alsahafi (2026)	1. Many Hausa speakers have forgotten the language, and some even regard it as a dialect. This has led to a reluctance to speak it within their families, even though it is one of the ten most important languages in the world. (p. 3163); 2. The Hausa language holds limited significance in Saudi Arabia. Learning it might be seen as a waste of time for those uninterested in their African heritage or who find it irrelevant to their professional goals. (p. 3165)	A(1). A portion of the Hausa community cognitively categorize Hausa as a dialect rather than an independent language, which undermines its perceived legitimacy and willingness of use in family settings; B(2). Hausa is perceived to have limited sociocultural and instrumental value in the Saudi context, deemed irrelevant to professional advancement for those without strong heritage engagement.
3	Baranova (2026)	1. Language attitudes in the community range from supportive to critical, often influenced by perceptions of linguistic authenticity. (p. 1); 2. That's the problem with the Kalmyk language – it's that the people who know it well, they criticize those who don't. Even though they had certain conditions, a certain environment that allowed them to learn it, they kind of look down on those who don't know it. (p. 11)	A(1). Cognitive evaluations of non-standard Kalmyk heritage language varieties span a supportive-to-critical continuum, primarily mediated by judgments of linguistic authenticity; B(2). Proficient traditional Kalmyk speakers hold negative cognitive stances toward less proficient speakers, criticizing their language use and devaluing non-standard competence as inferior.
4	Beaudrie et al. (2019)	1. "People from Spain speak the purest form of Spanish." (p. 12); 2. "I believe Spanish-speaking Hispanics in the United States don't speak correct Spanish." (p. 12); 3. "I don't like it when people codeswitch because it is not a proper way of speaking a language." (p. 13); 4. "I believe Spanglish should not be allowed in Spanish classes." (p. 13)	A(1-2). Learners internalize standard language ideologies that position Peninsular Spanish as the authentic, pure norm while deeming U.S. Spanish varieties linguistically incorrect; B(3-4). Learners hold negative cognitive judgments of Spanish-English code-switching, framing it as improper linguistic practice and inappropriate for formal instructional contexts.

No.	Author (Year)	Original Excerpt – Cognitive Dimension	Thematic Summary – Cognitive Dimension
5	Callesano & Carter (2019)	1. Results of the ANOVA of the ratings of the three dialects show that the peninsular Spanish voice was rated significantly more competent as compared to the Colombian and Cuban speakers (p = 0.000). (p. 91); 2. Spanish speakers, especially Cuban Americans, not only think of Peninsular Spanish as more correct in the abstract when asked to rate Spanish dialects, as in Alfaraz's studies, but also make predictive judgments that favor Peninsular speakers in the domains of income, profession, and personality traits related to competence. (p. 95)	A(1). Peninsular Spanish (the European standard variety) is cognitively attributed significantly higher competence-related traits (intelligence, trustworthiness, self-confidence) than Cuban and Colombian Spanish varieties by Miami Latinx listeners; B(2). Cognitive judgments of Spanish dialect legitimacy and correctness are intertwined with socioeconomic and occupational stereotypes, whereby standard Peninsular Spanish is systematically privileged over Latin American vernacular varieties.
6	Fatima & Nadeem (2025)	1. "I think Punjabi is neither considered a professional language at all. Nor it is associated with people who are very professional." (p. 10); 2. "Dominant languages, like English, are prioritized in educational and professional settings instead of heritage languages, leaving little to no room for support toward these heritage languages." (p. 10)	A(1). Heritage languages such as Punjabi are cognitively stereotyped as non-professional linguistic varieties, not associated with high-status professional identities in Pakistani society; B(2). Dominant languages (English, Urdu) are perceived as the legitimate and appropriate codes for educational and professional domains, while heritage languages are cognitively marginalized in formal institutional contexts.
7	Gao & Liu (2025)	1. Language status motivates individuals to adopt a dominant or more favourable language for social and economic reasons. In contrast, minority identity represents a desire to maintain one's heritage language as a marker of cultural or group identity. (p. 7); 2. According to the survey results, minority Wa individuals speak only minority Wa language, while minority Lahu individuals are open to the minority Wa language, thus forming the phenomenon of language segregation between minority Wa individuals with a strong minority identity and minority Lahu individuals with a sense of openness to the outside world. (p. 11)	A(1). Cognitive judgments of language dynamics are shaped by both perceived instrumental status of linguistic varieties and strength of minority identity, challenging the prior assumption that language status alone drives language outcomes; B(2). Strong minority identity cognitively frames heritage language as an exclusively in-group code, while weaker identity enables cognitive openness to other minority languages, resulting in patterns of linguistic segregation.
8	Gavriilidou & Mitits (2021)	1. The results showed (a) a decline in the use of Greek as HSs age and (b) an extended use of Greek only at home or with family. When asked which language(s) they spoke with their friends, the participants reported the host country language first (n=39, 72.2%). (p. 83); 2. The genres that most respondents find difficult to read are academic/ technical papers, poetry, novels, theatrical plays, non-fiction, and textbooks, while only giving a formal presentation was rated as challenging when speaking Greek. (p. 86)	A(1). Greek heritage language is cognitively categorized as contextually restricted to informal familial domains, while English as the dominant standard language is perceived as the legitimate and appropriate code for public, social, and broader communicative contexts; B(2). Learners cognitively recognize the limited functional utility of Greek in formal, academic, and professional registers, judging their own Greek competence as inadequate for advanced written and formal spoken tasks.

No.	Author (Year)	Original Excerpt – Cognitive Dimension	Thematic Summary – Cognitive Dimension
9	Grgič & Guardiancich (2025)	1. Slovenian speakers in Italy perceive supra-regional standard and non-standard varieties as something foreign, remote, even undesirable and non-adequate, while local varieties, from narrower dialectal to broader regional ones, are considered entirely and exclusively acceptable in all settings. (p. 31); 2. Inverse diglossia is observed: the Slovenian minority language plays a strong symbolic and ritualistic role in public intracommunity settings (celebrations, schools, media, formal meetings), while the majority Italian language gradually displaces the minority language in informal interpersonal communication. (p. 31)	A(1). An entrenched local-language ideology cognitively legitimizes local Slovenian varieties as the only acceptable codes across all contexts, while supra-regional standard Slovenian varieties are devalued as foreign, inadequate, and undesirable; B(2). An inverse diglossia cognitive framework structures domain appropriateness: the heritage minority language is deemed suitable for formal symbolic public domains within the community, whereas the dominant majority language is perceived as the default for informal interactional domains.
10	Inan & Harris (2025)	1. Many mainstream education systems in the United States and other monolingual-oriented countries fail to reflect the linguistic diversity of their student populations. This failure often leads to the marginalization of heritage languages and reinforces assimilationist pressures. (p. 02); 2. Some families view bilingualism as a socioeconomic asset that can enhance career prospects and global mobility, motivating them to seek out both community and digital resources; however, others—particularly those facing economic pressures—may prioritize dominant language acquisition for perceived practical advantages. (p. 02)	A(1). Dominant standard languages are cognitively legitimized as the normative code in mainstream educational and societal institutions, while heritage languages are framed as marginal and counter to assimilationist expectations; B(2). Cognitive judgments of heritage language utility vary by socioeconomic position: privileged groups frame heritage language/bilingualism as a professional and mobility asset, while economically disadvantaged groups prioritize dominant language acquisition for perceived immediate practical benefits.
11	Kovacs Rac & Halupka-Rešetar (2018)	1. Response (b) was the one that was most often opted for by the research participants (41.23%), i.e. that the dialect is the language nearly everybody speaks in the settlement, the language they learnt from their parents or grandparents. We do not read or write in this language, we only use it in speaking. (p. 131); 2. A total of 37.24% of the respondents reported having been stigmatized in some way because of their dialect or use of language. (p. 137)	A(1). Local Hungarian heritage dialects are cognitively categorized as informal spoken varieties acquired through intergenerational family transmission, functionally restricted to oral communication and distinct from the standard written language used in formal educational contexts; B(2). Heritage dialects are associated with negative social stereotypes, as over one-third of minority pupils have experienced mockery or criticism for their dialect use, reflecting cognitive judgments of dialects as linguistically non-standard and socially devalued.
12	Li (2026)	1. Between Putonghua and fangyan, I would choose Putonghua, because it can be spoken all over China, whereas fangyan is only used in one place and can't be used elsewhere. (p. 11); 2. If a child is exposed to fangyan as well, it may lead to some confusion, and Chinese pronunciation may become less standard. (p. 12)	A(1). Standard Putonghua is cognitively legitimized as the universally functional and nationally applicable linguistic variety, while local heritage fangyan are categorized as geographically restricted forms with limited practical utility; B(2). Parental beliefs are shaped by standard language ideology, which frames early exposure to heritage dialects as a threat to the correctness and phonetic standardization of children's Putonghua.

No.	Author (Year)	Original Excerpt – Cognitive Dimension	Thematic Summary – Cognitive Dimension
13	Makarova et al. (2019)	1. The ability to speak Russian is considered by parents to be an essential part of Russian identity; at the same time, the participants agree that their children learning English is more important than their learning Russian for their lives in Canada. (p. 466); 2. They agree that learning Russian is beneficial for their children's future life and career, and believe that learning two languages (Russian and English) can help their child to be smarter. (p. 466)	A(1). Russian heritage language is cognitively constructed as a core component of ethnic identity for Russian-speaking communities, while English as the official dominant language of Canada is perceived as having higher functional priority for children's life in the host country; B(2). Parents hold cognitive beliefs about the instrumental value of Russian for children's future life and career advancement, as well as the cognitive benefit of bilingualism that mastering both Russian and English can enhance children's intelligence.
14	Prada (2019)	1. Prior to attending that semester of Spanish for HSs, all participants considered that Spanglish did not belong in the classroom setting; many participants reported having heard that mixing languages or using Spanglish is typical of uneducated people or people who cannot speak either language properly. (p. 314–315); 2. Other Spanish professors or Spanish teachers wouldn't allow any use of Spanglish in our classes. They wanted an all-Spanish environment; "Standard Spanish" was framed as Castellano [Castilian Spanish], the normative variety expected in academic settings. (p. 314, 317)	A(1). Before the translanguaging-focused course, Spanish heritage speakers cognitively judge Spanglish and code-mixing practices as illegitimate and inappropriate for academic classroom contexts, and endorse the social stereotype that mixed language use signals low educational attainment and defective linguistic competence; B(2). Learners internalize monoglossic standard language ideologies that position Castilian Spanish as the only legitimate, correct, and educationally appropriate linguistic variety, while vernacular US Spanish varieties and translanguaging practices are categorized as substandard and improper for formal learning contexts.
15	Shabtaev et al. (2026)	1. Russian enjoys considerable recognition in Israel's public, governmental, commercial and educational institutions, and maintains a strong global heritage status grounded in cultural importance and political capital; by contrast, Juhuri is perceived to have limited practical value, with cognitive evaluations significantly lower than affective appraisals across middle and younger generations in Israel. (p. 632–633); 2. In Israel, ethnic identity significantly correlates with cognitive attitudes toward both Juhuri and Russian; in the United States, the correlation between ethnic identity and cognitive attitudes holds only for Juhuri, and dissipates entirely for Russian among second-generation immigrants. (p. 628, 633)	A(1). The former official standard language Russian is cognitively attributed high institutional prestige, practical utility and global status, while the traditional minority heritage language Juhuri is cognitively devalued as functionally limited, with its attitudinal significance primarily residing in affective rather than pragmatic domains；B(2). The cognitive association between ethnic identity and heritage language evaluation is moderated by national context and language type: it is robust for both languages in Israel, but in the U.S. it is restricted to Juhuri and breaks down for Russian across immigrant generations.

No.	Author (Year)	Original Excerpt – Cognitive Dimension	Thematic Summary – Cognitive Dimension
16	Siebenhütter (2021)	1. Kui, Lao (as spoken in Laos) and Lao NET were rated as low prestige languages (LPL) in terms of social status, while Thai and Chinese were ranked as high prestige languages (HPL); even though Lao NET is used by almost one-third of Thailand's population, its speakers are often stigmatised as uneducated and backward. (p. 20); 2. The Kui language is frequently used in speakers' homes and among friends/neighbors/children from their communities, but is used less in more public situations such as at the market and around outsiders; young speakers find Thai more practical for public and cross-community communication. (p. 9, 18)	A(1). A cognitive prestige hierarchy positions standard Thai as a high-status legitimate language, while Kui heritage dialect and regional Lao varieties are categorized as low-prestige linguistic forms, with Lao NET speakers associated with the negative social stereotype of being uneducated and backward; B(2). Speakers hold domain-specific cognitive judgments of language appropriateness: Kui heritage dialect is deemed suitable only for informal in-group contexts (home, local community), whereas standard Thai is perceived as the more practical and appropriate code for public, market, and cross-community communicative contexts.
17	Vari & Tamburelli (2023)	1. Participants in Luxembourg had higher D-scores (mean = 0.28; s.d. = 0.44), i.e. associating their vernacular more strongly with positive evaluations when compared to Belgian participants (mean = −0.31; s.d. = 0.44); Luxembourgish average D-scores were positive, showing a preference for the vernacular over the standard, whereas Belgian participant D-scores were negative, indicating a preference for the standard over the vernacular. (p. 458); 2. Speech community was the strongest of all predictor variables, suggesting that how endangered vernaculars are standardised influences attitudes towards them more than any other sociolinguistic factor such as age or gender; only the introduction of a close standard can lead to the intended protective effect on vernacular attitudes, while a distant standard benefits only the standard itself to the detriment of the endangered vernaculars. (p. 459)	A(1). Standardisation strategy shapes implicit cognitive evaluations of heritage dialects: the Luxembourg community with an endogenous, linguistically close standard shows more positive implicit attitudes toward the local vernacular than toward the standard, while the Belgian community using an exogenous, linguistically distant standard (Standard German) cognitively prefers the standard over the vernacular; B(2). Linguistic distance between the standard variety and the heritage dialect is the strongest predictor of cognitive attitudes: a close standard extends its positive prestige evaluation to the vernacular, whereas a distant standard fails to transfer positive evaluations, leaving the vernacular cognitively devalued relative to the standard.
18	Yiakoumetti (2022)	1. Teachers' language choices were driven by their beliefs that students associated the second language (Mandarin) with teaching and the first language (English) with more familiar, casual and comfortable settings. (p. 7); 2. Students very often do not see a reason for learning other languages because of the unprecedented power of English; some parents do not wish for their child to be labelled as a speaker of Chinese-as-a-foreign-language. (p. 8, 9)	A(1). Teachers hold domain-differentiated cognitive beliefs about language appropriateness: Mandarin as the heritage language is cognitively framed as a code for formal teaching contexts, while English as the dominant societal standard language is associated with informal, familiar and comfortable interpersonal settings, which shapes teachers' in-class language use；B(2). The high global status of English underpins cognitive judgments of heritage language utility: students perceive little practical value in learning Chinese, and some parents reject categorizing Chinese as a foreign language for their children, reflecting contested cognitive classification and legitimacy of the heritage variety in the host society.

Table B. Affective Dimension

No.	Author (Year)	Original Excerpt – Affective Dimension	Thematic Summary – Affective Dimension
1	Abedini (2025)	1. Persian strengthens intercultural identity by serving as an emotional anchor, fostering connections to Iranian heritage through family interactions, cultural practices, and personal pride. (p. 9); 2. participants' intercultural identities contributed to emotional distancing from Persian, driven by a preference for English due to its association with comfort and confidence in the host culture, coupled with discomfort with Persian stemming from social pressures and experiences of marginalization. (p. 11)	A(1). The Persian heritage language operates as an affective anchor that generates feelings of familial closeness, cultural belonging and personal pride, and this emotional investment mutually reinforces speakers' intercultural identity；B(2). Affective experiences toward heritage language are ambivalent: pride in cultural heritage coexists with shame induced by negative societal stereotypes, and preference for the dominant host language can produce emotional detachment from the heritage language.
2	Alsahafi (2026)	1. G2 participants exhibited relatively higher proficiency levels and more positive attitudes toward Hausa compared to G3 participants; the second generation sees Hausa as an important part of their ethnic identity, and both generations recognise its vital role in creating unity within the Hausa community. (p. 3154, 3162); 2. Many Hausa people experience deep pain, especially some from the second generation, because their children do not know the Hausa language, a frustration linked to the declining use of Hausa in family settings. (p. 3164)	A(1). Affective attachment to the Hausa heritage language shows generational variation: the second generation holds stronger emotional identification with Hausa as a core component of ethnic identity and a unifying affective bond for the community, while the third generation displays weaker affective investment in the language； B(2). Intergenerational erosion of heritage language competence triggers deep emotional pain and frustration among community members, particularly pronounced in the second generation, as reduced home use of Hausa disrupts transmission to younger cohorts.
3	Baranova (2026)	1. "Now I regret it, I mean, it was a mistake not to study it. It's a shame I don't know my own language." (p. 11); "They're afraid of pronouncing something incorrectly; they're embarrassed that the person next to them might hear and laugh. That's why we have this problem: people are afraid to speak because they think they'll say something wrong and end up in an awkward situation." (p. 10); 2. "the people who know it well, they criticize those who don't. Even though they had certain conditions, a certain environment that allowed them to learn it, they kind of look down on those who don't know it." (p. 11); "Don't speak, you have a terrible Russian accent!" (p. 12)	A(1). Deficient competence in the Kalmyk heritage language generates negative affective responses: young ethnic members experience shame and regret over not mastering their ancestral language, while learners in language courses feel social embarrassment and linguistic anxiety, avoiding spoken production for fear of mispronunciation and peer mockery； B(2). Proficient traditional Kalmyk speakers hold critical and dismissive stances toward speakers with non-standard or limited language ability; such in-group negative evaluation erodes learners' motivation and can lead to temporary disengagement from language learning.

No.	Author (Year)	Original Excerpt – Affective Dimension	Thematic Summary – Affective Dimension
4	Beaudrie et al. (2019)	1. "People from Spain speak the purest form of Spanish." (p. 12); "I feel speaking Spanish is less prestigious than speaking other foreign languages such as French." (p. 12); "I believe Spanish-speaking Hispanics in the United States don't speak correct Spanish." (p. 12); 2. "I don't like it when people codeswitch because it is not a proper way of speaking a language." (p. 12); "I would not codeswitch in front of my teachers because they may think I am less intelligent." (p. 13)	A(1). Spanish heritage learners internalize standard language ideologies that position Peninsular Spanish as the pure, legitimate norm while deeming U.S. Spanish varieties linguistically incorrect, and perceive Spanish as lower in prestige than other foreign languages, reflecting diminished affective valuation of their own heritage linguistic variety and embedded linguistic insecurity；B(2). Learners hold negative affective attitudes toward Spanish-English code-switching, framing it as an improper way of speaking, and experience social anxiety around language use, fearing that code-switching in front of teachers will result in being judged as less intelligent.
5	Callesano & Carter (2019)	1. "the peninsular Spanish voice was rated significantly more competent as compared to the Colombian and Cuban speakers. We found no significant difference for the aggregated warmth traits." (p. 91); "participants rated the Cuban voice significantly lower than the Colombian and Peninsular voices for physical attractiveness. This difference in perception is significant." (p. 92); 2. "participants rated the Colombian voice to be significantly more likely to learn English within the next year, significantly more likely to watch television mostly in English, and significantly less likely to teach Spanish to his children, as compared to the other voices." (p. 93)	A(1). Latinx listeners in Miami hold affectively hierarchical perceptions of Spanish varieties: Peninsular standard Spanish is associated with higher competence traits (intelligence, self-confidence, trustworthiness), while Cuban and Colombian heritage varieties receive lower competence ratings; warmth evaluations do not differ across varieties, yet the Cuban variety is judged lower in physical attractiveness, reflecting affective stereotypes tied to language origin；B(2). Affective expectations about language shift and maintenance vary by dialect: the Colombian variety is affectively linked to stronger English orientation and weaker intergenerational Spanish transmission, whereas Cuban and Peninsular varieties are perceived as more likely to pass Spanish to children; national-origin background labels further modulate affective judgments about language loss anxiety and language choice in bilingual settings.

No.	Author (Year)	Original Excerpt – Affective Dimension	Thematic Summary – Affective Dimension
6	Fatima & Nadeem (2025)	1. Expressing a sense of connection and pride in their linguistic heritage, participants viewed language as a huge part of preserving culture and ethnicity and fostering a certain sense of belonging to their community. (p. 9); when I was a child, when I was hit by my mother, the exchange was in Siraiki. And it continued and became a language of hatred for me. Whenever there was violence, it was always Siraiki. (p. 10); 2. I think Punjabi is neither considered a professional language at all. Nor it is associated with people who are very professional. So I think when it comes to societal pressure that was again a very big, contributing factors in me, not trying to learn Punjabi, or speak at it very commonly because it wasn't considered professional. (p. 10); older generations, particularly grandparents and great grandparents, often converse exclusively in their heritage languages and frown upon the use of other languages within the household. Younger generations, in contrast, were reported to either exhibit the use of a mix of primary and heritage languages-frequently code-switching-or favoring culturally dominant languages altogether. (p. 7)	A(1). Heritage languages serve as a core affective marker of ethnic identity that fosters cultural pride and a sense of community belonging; yet affective responses to heritage languages are ambivalent, as negative personal experiences tied to language use can generate emotional alienation and associate the heritage variety with shame, belittlement and hostility；B(2). Local heritage dialects carry negative social stereotypes of being unprofessional and low-status in mainstream societal contexts, creating stigma that discourages public use and fuels linguistic insecurity; generational gaps in affective attachment are pronounced, with older generations holding strong emotional loyalty to heritage languages while younger generations show weaker affective investment and prioritize dominant languages.
7	Gao & Liu (2025)	1. "In contrast, minority identity represents a desire to maintain one's heritage language as a marker of cultural or group identity." (p. 7); "According to the survey results, minority Wa individuals speak only minority Wa language, while minority Lahu individuals are open to the minority Wa language, thus forming the phenomenon of language segregation between minority Wa individuals with a strong minority identity and minority Lahu individuals with a sense of openness to the outside world." (p. 11); 2. "Under a certain transmission rate C, the density of individuals w in the group increases that increasing self-minority transmission rate, and the self-minority identity transmission rate has an obvious positive effect on individuals w, both in the ER and the BA networks, especially in the BA network." (p. 10)	A(1). Minority identity functions as an affective motivation for heritage language maintenance, framing the heritage language as a core marker of cultural and group belonging; strong minority identity correlates with exclusive affective attachment to one's own heritage language and linguistic segregation from other ethnic groups, while more open identity orientation corresponds to receptiveness toward other minority linguistic varieties；B(2). Self-transmission of minority identity strengthens affective identification and sense of belonging to one's heritage language, which in turn increases the proportion of individuals who exclusively use their minority language; this identity transmission effect positively promotes minority language diffusion across both homogeneous and heterogeneous social networks, with a stronger effect observed in heterogeneous scale-free networks.

No.	Author (Year)	Original Excerpt – Affective Dimension	Thematic Summary – Affective Dimension
8	Gavriilidou & Mitits (2021)	1. the overwhelming majority of participants reported having their family's support in maintaining Greek (98.1%). Similarly, all of them would want to teach their children Greek (100%). (p. 87); Greek is viewed as a symbol of GHSs' ethnolinguistic identity, culture, and history that has to be preserved and there is a subsequent strong desire, especially from the first and second generation, to maintain it. (p. 88); 2. First-generation HSs demonstrate high proficiency in Greek and claim they are Greek because they were born and raised in Greece. They relate to Greek more than to American culture... feel nostalgic about what they left behind when they immigrated, and have a sense of belonging to and love of Greece. (p. 89); in cases where second-generation parents did not invest in the preservation of Greek culture... third-generation children feel mainly American, distance themselves from Greek customs, traditions, and/or cuisine, and very often demonstrate a low proficiency in Greek. (p. 90)	A(1). The Greek heritage language is affectively constructed as a central symbol of ethnolinguistic identity, cultural heritage and historical continuity; community and family support for heritage maintenance is widespread, and there is a strong affective commitment across first and second generations to preserve and transmit Greek to future generations；B(2). Affective attachment and sense of belonging to the heritage language vary sharply by generation: first-generation immigrants hold deep emotional bonds, nostalgia and a strong sense of belonging to Greek culture, while third-generation speakers with limited familial cultural investment exhibit affective distancing from Greek heritage and primarily identify with the host society's culture.
9	Grgič & Guardiancich (2025)	1. Slovenian speakers in Italy perceive supra-regional standard and non-standard varieties as something foreign, remote, even undesirable and non-adequate, while local varieties, from narrower dialectal to broader regional ones, are considered entirely and exclusively acceptable in all settings. (p. 31); speakers' attitude towards their own language is dominated by a sense of pride: according to the speakers interviewed, this is also a characteristic feature of Slovenians in Italy and a strong identity-forming element, while Slovenians in Slovenia are "less proud of their language". (p. 41); 2. Two ideological cores can be observed among community members: the first-language ideology erecting a barrier between Slovenians and Italians and the language-pride ideology raising a barricade between the Slovenians in Italy and those in Slovenia; both serve the exclusivism of the regional standard and the preservation of communication practices and identification options within the local community. (p. 40)	A(1). Local Slovenian heritage dialects are infused with strong affective pride and identity-forming significance, and are judged as the only fully legitimate and acceptable linguistic forms; supra-regional standard Slovenian varieties, by contrast, are affectively constructed as foreign, remote and undesirable, embodying an inverse local-language ideology that upends the standard prestige hierarchy；B(2). Two emotionally charged language ideologies structure group boundaries: a first-language ideology creates affective and social barriers between the minority community and the Italian majority, while a language-pride ideology establishes emotional distance from Slovenian speakers in the homeland; both ideologies reinforce the exclusivity of the local linguistic norm and in-group identity cohesion.

No.	Author (Year)	Original Excerpt – Affective Dimension	Thematic Summary – Affective Dimension
10	Inan & Harris (2025)	1. Community organizations serve as safe and affirming spaces where bilingual families can build networks, reinforce their language practices, and strengthen their sense of belonging. (p. 2); Schools that adopt inclusive curricula can signal to students and families that their languages and cultures are valued. (p. 2); 2. Societal attitudes and employment concerns lead many parents to deprioritize heritage language use, particularly among families facing economic pressures. (p. 2); macro-level societal attitudes toward minority languages, alongside conflicting standard language ideologies in schools and communities, often create barriers that diminish the effectiveness of family heritage language maintenance efforts. (p. 1)	A(1). Institutional and community support generates positive affective outcomes for heritage language speakers: inclusive school curricula deliver affective validation of minority linguistic and cultural identities, while community-based heritage language initiatives provide safe, affirming social spaces that strengthen speakers' sense of group belonging and reinforce their language engagement；B(2). Negative societal attitudes and assimilationist standard language ideologies in public contexts constitute affective barriers to heritage language maintenance; socioeconomic pressure and employment-related concerns further erode affective commitment to heritage languages and drive prioritization of the dominant standard language among immigrant families.
11	Kovacs Rac & Halupka-Rešetar (2018)	1. Approximately half of the respondents claimed to like the dialect spoken in their place of residence, not to feel embarrassed by it and to use it often in public; 77.24% of the respondents consider the dialect spoken in their place of residence attractive. (p. 132, 136); a total of 37.24% of the respondents reported having been stigmatized in some way because of their dialect or use of language, and 10.33% liked their dialect but used it rarely due to fear of being judged, which conveys the covert prestige of the dialect. (p. 132, 137); 2. 22.33% of the responses suggested that speaking in their dialect was a sign of the respondents' love for the town or village where they live. (p. 134)	A(1). Hungarian minority pupils hold predominantly positive affective attitudes toward their local heritage dialect, with large majorities rating it attractive and reporting no embarrassment using it in public; however, social stigma around the dialect persists, as over one-third of students have experienced mockery or criticism for their speech, and a smaller group restricts dialect use due to fear of judgment, reflecting a pattern of overt positive regard and covert prestige coexisting with public stigmatization；B(2). Heritage dialect use is emotionally tied to place-based attachment: speaking the local dialect is framed by students as an expression of love for their hometown, indexing affective connection to their place of residence.

No.	Author (Year)	Original Excerpt – Affective Dimension	Thematic Summary – Affective Dimension
12	Li (2026)	1. "I still think fangyan is also very important, because after all there are still elderly people. My thinking is a bit traditional: Chinese people are Chinese, after all, and we will certainly return to our roots, whatever happens in the future. So, it's important to learn some of the hometown language as well as Putonghua-both matter." (p. 12); "Fangyan is not particularly useful for him anyway. Putonghua is already enough in China […] Between Putonghua and fangyan, I would choose Putonghua, because it can be spoken all over China, whereas fangyan is only used in one place and can't be used elsewhere." (p. 10); 2. "She likes speaking Chinese, so she often speaks it in class […] Even when we discuss the presence of many different ethnicities in the class and emphasise it as a resource, she becomes enthusiastic and starts saying words in Chinese" (p. 14); "She only speaks about Chinese when we encourage her to share a few words with the class… she does so in such a low voice that I can't hear her." (p. 15)	A(1). Affective valuation of heritage linguistic varieties is stratified among Chinese migrant families: local fangyan dialects carry divergent affective meaning, with a minority of parents framing them as emotionally significant markers of family continuity, ancestral roots and traditional ethnic identity, while the majority ascribe low affective and practical value to dialects and position standard Putonghua as the only legitimate, prestigious heritage language worthy of intergenerational transmission；B(2). Children's affective responses to heritage language use in public school contexts are heterogeneous: some children demonstrate enthusiasm and pride when given opportunities to display their heritage Chinese in multicultural classroom settings, while others exhibit linguistic insecurity and reticence, speaking the heritage language only when prompted and in quiet, hesitant ways.
13	Makarova et al. (2019)	1. "Overall, the data demonstrate that parents of bi/multilingual children have very strong positive attitudes toward Russian language and culture. They show no desire to be "assimilated," and want Russian language maintained in the family. The ability to speak Russian is considered by parents to be an essential part of Russian identity." (p. 466); 20 out of 30 participants strongly agreed that "learning Russian is essential for keeping contact with family in Russia/other Russian-speaking country". (p. 466); 2. "I feel sorry for Canadians with Russian-speaking ancestry who don't know Russian" received 10 agree and 5 strongly agree responses among 30 participants; the majority of respondents (17 of 30) reported dissatisfaction with the Russian-speaking milieu in Saskatchewan, and 15 of 25 parents stated concerns about their children's Russian language development. (p. 465, 466)	A(1). Russian-speaking immigrant parents hold strong affective identification with the Russian heritage language, framing it as an essential component of Russian ethnic identity; they reject linguistic assimilation into the host society and express commitment to maintaining Russian in family life, while also viewing the language as vital for sustaining transnational family ties；B(2). Affective experiences surrounding heritage language maintenance include sympathetic regret for co-ethnic community members who lose proficiency in Russian, dissatisfaction with the limited heritage language environment in the host region, and widespread parental concern about children's Russian language development trajectories.

No.	Author (Year)	Original Excerpt – Affective Dimension	Thematic Summary – Affective Dimension
14	Prada (2019)	1. "I was very embarrassed speaking to others because my Spanish was not perfect but was expected since I am Hispanic." (p. 314); "I have heard this plenty of times from all my family members and especially my parents" regarding the idea that mixing languages is typical of uneducated people. (p. 315); 2. "this class has definitely made me view my languages in a different way, it has given me a sort of pride or acceptance of my Spanglish. Before, I would feel ashamed of not knowing the language as well as I should but now it's shifted into feeling like 'well, I know how to speak two languages fairly well instead of just knowing how to speak only one'." (p. 317)	A(1). Spanish heritage language learners bring pre-existing affective distress to academic contexts: they experience embarrassment and linguistic insecurity over their perceived non-standard Spanish proficiency, and internalize familial and societal stigma that frames code-mixed practices such as Spanglish as a marker of low educational attainment and inadequate language competence；B(2). Translanguaging pedagogy triggers positive affective reorientation among heritage speakers: learners shift from shame and self-criticism about their mixed linguistic repertoires to pride and acceptance of their bilingual practices, reframing dual-language competence as a personal strength rather than a linguistic deficit.
15	Shabtaev et al. (2026)	1. "The strongest generalization that emerges from visual inspection of the graphs is that the Affective component is more salient than the other two attitude components. In Israel, attitudes toward Juhuri and Russian decline across generations for all three attitudinal components, yet the Affective component of Juhuri remains robust among both G1.5 and G2 respondents, reflecting the emotional value attached to this heritage language. In the United States, affective attitudes toward Juhuri rise across generations, indicating a strong emotional connection and heritage motivation." (p. 627, 632); 2. "In Israel, all correlations between ethnic identity and language attitudes were significant for both Juhuri and Russian. In the United States, correlations between ethnic identity and language attitudes were significant only for Juhuri, and the connection between Russian and Mountain Jewish identity was lost across generations to the point of no significant correlations among second-generation participants. The effect of ethnic identity commitment on attitudes is stronger for Juhuri than for Russian, especially for the Affective component." (p. 628, 633)	A(1). The affective dimension is the most salient component of heritage language attitudes among Mountain Jewish speakers; affective attachment to the traditional Juhuri heritage language remains resilient across generations in Israel despite overall attitudinal erosion, and strengthens across generations in the US as a unique vessel of Jewish cultural heritage, while affective regard for Russian exhibits generational decline in the Israeli context；B(2). Ethnic identity strength significantly shapes affective evaluations of heritage languages: ethnic commitment exerts a stronger effect on affective attitudes toward the ethnically distinct Juhuri than toward the more widespread Russian; this identity-affect linkage operates for both heritage languages in Israel, but in the US it is exclusive to Juhuri, with the affective bond between Russian and group identity completely dissipating among the second generation.

No.	Author (Year)	Original Excerpt – Affective Dimension	Thematic Summary – Affective Dimension
16	Siebenhütter (2021)	1. "In Thailand, stigmatisation of minority groups and their respective languages is still a significant problem... one speaker did not mention his Kui background at his workplace in Bangkok, fearing negative consequences." (p. 19); "The young speakers were not ashamed of their linguistic background, enjoyed being bi- or multilingual and avoided rating one language over another." (p. 20); 2. "there are several motivations for shifting to Thai: communicative needs... the feeling of belonging to a group (identification with a group) and shame to be a member of a minority group and the hope to gain a better reputation by using Thai (language prestige)." (p. 21); "young Kui speakers still have a deep connection to their cultural heritage and linguistic background... the prestige of Thai is not a strong enough reason for young Kui speakers to give up their minority language altogether." (p. 20)	A(1). Linguistic stigma persists in broader Thai society, prompting some Kui heritage speakers to conceal their ethnolinguistic background in public professional settings to avoid negative repercussions; nonetheless, young Kui speakers overall report no shame about their linguistic heritage, hold positive attitudes toward their bi/multilingual repertoires, and resist hierarchically ranking one language above another；B(2). Affective and identity-based factors shape language choice patterns: a deep sense of cultural connection and group belonging sustains Kui use in in-group contexts, while minority-status shame and the desire for higher social reputation associated with standard Thai drive code-switching to Thai in cross-group public settings; yet the prestige of the dominant standard language is insufficient to fully displace affective attachment to the heritage language.
17	Vari & Tamburelli (2023)	1. "Overall, participants in Luxembourg had higher D-scores (mean = 0.28; s.d. = 0.44), i.e. associating their vernacular more strongly with positive evaluations when compared to Belgian participants (mean = −0.31; s.d. = 0.44). Luxembourgish average D-scores were positive (mean = +0.28), showing a preference for the vernacular over the standard, whereas Belgian participant D-scores were negative (mean = −0.31), indicating a preference for the standard over the vernacular." (p. 458); 2. "speech community was the strongest of all predictor variables, suggesting that how endangered vernaculars are standardised influences attitudes towards them more than any other sociolinguistic factor such as age or gender... speakers have more positive attitudes towards their vernacular in speech communities with a linguistically closer standard variety." (p. 459)	A(1). Implicit affective evaluations of heritage vernaculars diverge sharply across standardisation contexts: Luxembourgish Moselle Franconian speakers hold positive implicit affective preference for their local vernacular over the endogenous standard variety, while Belgian speakers of the same dialect family exhibit negative implicit affective bias toward their vernacular and favour the exogenous Standard German；B(2). Linguistic distance between the standard and the vernacular is the strongest predictor of affective valence toward minority dialects: a linguistically close endogenous standard enables positive affective evaluation to transfer from the standard to the heritage vernacular, whereas a distant exogenous standard fails to confer affective prestige on the vernacular and instead reinforces the standard’s affective dominance.

No.	Author (Year)	Original Excerpt – Affective Dimension	Thematic Summary – Affective Dimension
18	Yiakoumetti (2022)	1. "they admitted to feelings of guilt for employing this type of L1 function" (p. 5); "a sense of surprise and even guilt and regret was conveyed through teachers' discourse as they believed that they should have used students' target language in certain occasions." (p. 7); 2. "students' parents and even students who do not speak their heritage language nevertheless have a special affinity to their ethnic language and the culture that accompanies it. This strong bond with the language and culture of their ancestors" (p. 9); "a parent did not wish for her child to be labelled as a speaker of Chinese-as-a-foreign-language." (p. 7)	A(1). Heritage language teachers experience affective discomfort around their classroom language practices: they feel guilt, surprise and regret when they use students' dominant English (L1) for instructional purposes, as they internalize the normative belief that instruction should be delivered exclusively in the target heritage language (Mandarin)；B(2). Affective bonds with the heritage language persist across community members even when language proficiency is limited: parents and students hold a strong sense of affective affinity and identity attachment to Chinese as an ethnic language, and parents resist framing their children as foreign language learners of Chinese, reflecting symbolic investment in the language as part of ethnic identity.

Table C. Behavioral Dimension

No.	Author (Year)	Original Excerpt – Behavioral Dimension	Thematic Summary – Behavioral Dimension
1	Abedini (2025)	1. "We still do all the Persian traditions in our household such as Nowruz, Shab-e Yalda, Sizdah Bedar, and Chaharshanbe Suri. We also celebrate Christmas, put up a Christmas tree, and observe Thanksgiving, so we combine both traditions together." (p. 10); 2. "At our community's cultural fair, I shared Persian stories about Chaharshanbe Suri with friends, switching between Persian and English so everyone could join in. I taught my classmate Liam to say 'sobh bekheyr' (good morning) for fun, and we laughed as he nailed the accent. Mixing languages feels natural and keeps Persian exciting for me." (p. 11); 3. "Honestly, I cared a lot about traditions in Iran, but since migrating, they've become less important to me. Recently, during Chaharshanbe Suri and Nowruz, I had zero interest... Holidays like Christmas, Halloween, and Easter feel more important because everyone celebrates them." (p. 11–12); 4. "English makes me feel more English and reflects my new personality here. I don't feel much interest in 'being Iranian,' so English feels better to me. Over the past year, I've started to enjoy my English accent, which is great because I used to struggle with and feel ashamed of it. I used to avoid speaking to hide my accent, but now I'm happy with it." (p. 11); 5. "Many of us still felt the need to hide our Iranian culture or claim a more accepted background. Because of this, we rarely taught others about our language or traditions, and often said I was Persian rather than Iranian, since many confused it with Parisian... our heritage, culture, and language were often kept private." (p. 12)	A(1-2). 1.5-generation Iranian immigrants practice integrated bilingual and cultural behaviors: they combine Persian heritage festivals and host-country holiday traditions within family settings, and engage in spontaneous Persian-English code-switching during community social interactions, actively sharing Persian linguistic and cultural content with peers to sustain heritage language use；B(3-4). Some immigrants exhibit assimilation-oriented language behavioral shifts: they reduce participation in Persian cultural traditions and prioritize host-cultural holidays and practices, corresponding to contracted contexts for Persian use; their cultural identification shifts toward the host society, with language preference tilting to English, which becomes tied to self-expression, confidence and social integration；C(5). Negative societal stereotypes trigger language concealment behaviors: affected immigrants choose to hide their ethnocultural background in public settings, rarely teach Persian or share cultural traditions with others, and restrict heritage language and cultural practices to private intimate domains.

No.	Author (Year)	Original Excerpt – Behavioral Dimension	Thematic Summary – Behavioral Dimension
2	Alsahafi (2026)	1. "In the G2 group, 54 individuals (52%) reported Hausa as their first language. Arabic was the first language for 46 individuals (45%)... In contrast, the G3 group predominantly reported Arabic as their first language, with 53 individuals (70%). Hausa was reported as the first language for 22 individuals (29%)." (p. 3161); 2. "Hausa is less frequently spoken in these contexts among G3. This shift is reflected in the higher ratings for the statement AS4, 'Hausa is not used in my home', for G3 (M=2.50, SD=0.92) compared to G2 (M=2.19, SD=0.95)." (p. 3162); 3. "his parents, like many others in the community, spoke Arabic instead of Hausa to their children. He explained that he mainly learned the Hausa language outside of the home." (p. 3163); 4. "Many Hausa speakers have forgotten the language, and some even regard it as a dialect. This has led to a reluctance to speak it within their families..." (p. 3163); 5. "Parents should teach their children the Hausa language, and children should prioritise learning their native tongue... It is crucial for parents and grandparents who speak Hausa to interact with their children and grandchildren in this language at home to prevent it from disappearing from our society." (p. 3164); 6. "There is a need to establish schools and university programmes to teach Hausa in the Kingdom of Saudi Arabia... I wish special schools would be created to teach Hausa and English together." (p. 3164); 7. "language shift is likely inevitable due to Hausa’s minority status and its inability to compete with more powerful and useful languages being learned by Hausa-speaking Saudi children, such as English and Arabic." (p. 3165); 8. "The Hausa language holds limited significance in Saudi Arabia. Learning it might be seen as a waste of time for those uninterested in their African heritage or who find it irrelevant to their professional goals." (p. 3165)	A(1-2). Intergenerational language shift behavior: The third generation (G3) of Hausa Saudis demonstrates a clear behavioral shift in first-language dominance from Hausa to Arabic relative to the second generation (G2), paired with a measurable decline in the frequency of Hausa use in home settings；B(3-4). Disrupted heritage language reproduction behavior in domestic contexts: Most Hausa parents habitually select Arabic rather than Hausa for parent-child communication, resulting in broken intergenerational language transmission; a portion of community members devalue Hausa as a mere dialect and exhibit reluctance to use it in family interactions；C(5-6). Heritage language maintenance advocacy behaviors: Community members propose two core behavioral strategies for Hausa preservation: encouraging active intergenerational Hausa use within families led by parents and grandparents, and lobbying for the establishment of dedicated formal educational institutions (schools and university programmes) for Hausa language instruction；D(7-8). Language shift acceptance behavior: A subset of participants perceive Hausa language shift as a natural and inevitable outcome of its minority status; they judge Hausa to have limited practical utility in the Saudi context and regard learning it as a waste of time for individuals unconcerned with ethnic heritage or professional relevance.

No.	Author (Year)	Original Excerpt – Behavioral Dimension	Thematic Summary – Behavioral Dimension
3	Baranova (2026)	1. "I wrote five words at a time - taught myself. I wrote down the words one by one - and then it just kind of starts coming by itself. Like full sentences, how to address people. I wrote them down and studied every day... So I decided to study it myself. And that's how I learned it." (Ajsa, female, 1935) (p. 9); 2. "Bajerta was born in 1942 and, from 1943, was deported to a settlement in the Tyumen region (Siberia), where there was no Kalmyk linguistic environment. After returning from deportation, she enrolled in the Kalmyk and Mongolian department at the university in Elista, learned Kalmyk, and became a Kalmyk-language teacher." (p. 9); 3. "When he became a grandfather, he decided that it was necessary to become a Kalmyk speaker. He began reading the newspaper in Kalmyk, Xal'məg Ünən, and his special interest is folklore texts or Buddhist rituals and terminology." (Pavel, male, 63) (p. 9–10); 4. "apart from students, most attendees [of free Kalmyk courses] are pensioners. He notes that these learners often have more free time and feel a sense of responsibility for preserving family traditions, including the ethnic language." (p. 9); 5. "When I was a child... I could even talk to adults, I could respond to elders in Kalmyk. But later, during school, in high school, the ideas of globalization somehow influenced me, and I stopped paying proper attention to the Kalmyk language... Now I regret it, I mean, it was a mistake not to study it. It's a shame I don't know my own language." (Batyr, male, 1993) (p. 11); 6. "I had a classmate who spoke it very well, and at one point she said something that pushed me away from learning Kalmyk for a while: "Don't speak, you have a terrible Russian accent!"... I didn't like that attitude, so I decided to study on my own." (Sanal, male, 1999) (p. 12); 7. "they're afraid of pronouncing something incorrectly; they're embarrassed that the person next to them might hear and laugh. That's why we have this problem: people are afraid to speak because they think they'll say something wrong and end up in an awkward situation." (Nikolay, course instructor) (p. 10); 8. "the people who know it well, they criticize those who don't. Even though they had certain conditions, a certain environment that allowed them to learn it, they kind of look down on those who don't know it." (Mergen, male, 1997) (p. 11); 9. "My oldest son, he's in Canada. And he says, like, we're getting together, the Kalmyk diaspora, and they even sing Kalmyk songs in restaurants, play the dombra, he took the dombra over there." (Galina, female, 1958) (p. 12); 10. "I want to organize a Kalmyk-language camp in Europe next year. Because kids want to socialize, and parents also want them to socialize with other Kalmyks" (Savr, male, 1990, Germany) (p. 13); 11. "When I got to know people there [in Moscow], Buryats and so on, many of my Buryat acquaintances knew the Buryat language and at quite a good level. Somehow, I had this idea, like, why am I any worse? And I started learning Kalmyk." (Badma, male, 2000) (p. 12)	A(1-2). Post-deportation adult language re-acquisition behavior: Kalmyks who experienced interrupted family language transmission during Siberian deportation engage in deliberate self-directed Kalmyk learning after returning to Kalmykia; some pursue formal higher education in the language and become language teachers；B(3-4). Elderly pensioners' active language improvement behavior: Retired Kalmyks undertake voluntary Kalmyk proficiency enhancement motivated by responsibility for intergenerational family tradition transmission and grandparenting roles; they form a major participant group in free community language courses；C(5-6). Youth language behavior patterns: Young Kalmyks show childhood attrition of Kalmyk proficiency due to prioritization of the dominant language and globalized education, followed by later regret and active re-learning efforts; negative peer criticism of non-standard pronunciation can trigger temporary language avoidance and a shift to independent self-study；D(7-8). Community reactive behavior toward non-standard competence: The Kalmyk community exhibits ambivalent behaviors: learners feel public shame and fear of mockery for imperfect Kalmyk, and proficient speakers often criticize and look down on those with low proficiency, creating barriers to active language use；E(9-10). Diaspora language activation and community organizing behavior: Kalmyk diaspora communities engage in active heritage language practice (e.g., collective singing, cultural gatherings) in host countries; diaspora activists organize dedicated language camps for children to facilitate intergenerational transmission and ethnic socialization；F(11). Mobility-triggered language learning behavior: Intra-national migration to multi-ethnic urban contexts can activate Kalmyk language learning, driven by social comparison with other ethnic groups that maintain their heritage languages.

No.	Author (Year)	Original Excerpt – Behavioral Dimension	Thematic Summary – Behavioral Dimension
4	Beaudrie et al. (2019)	1. "If I were to give a professional presentation, I would try to avoid working with a partner who has a strong foreign accent in English." (p. 12); 2. "I would not codeswitch in front of my teachers because they may think I am less intelligent." (p. 13); 3. "I would be willing to join a club who meets every week to discuss how to maintain Spanish within the family and community." (p. 13); 4. "I would support U.S. Spanish to be taught in Spanish classes in the United States." (p. 13); 5. "Paired-sample t-tests performed on the pretest and posttest scores showed significant post-instruction changes for factor 1 [$t(18)=-2.38$, $p<.05$], factor 2 [$t(18)=-2.18$, $p<.05$], and factor 3 [$t(18)=-3.25$, $p<.05$]." (p. 11); 6. "The final 19-item instrument had adequate psychometric properties and detected change in the CLA of students in a class where CLA was taught." (p. 1)	A(1-2). Norm-conforming linguistic avoidance behaviors: Spanish heritage language learners internalize dominant standard-language and monolingual ideologies, displaying behavioral tendencies to avoid interaction with partners with strong non-native accents in professional contexts and to suppress code-switching in front of authority figures to avoid perceived negative evaluation；B(3-4). Heritage language maintenance and advocacy behavioral intentions: Learners express proactive willingness to engage in heritage language preservation practices, such as participating in community language maintenance clubs and advocating for the inclusion of U.S. Spanish in formal educational curricula；C(5-6). Intervention-driven behavioral change: Critical Language Awareness (CLA) instruction produces significant positive improvements in learners' language-related behavioral orientations across three domains (linguistic prejudice, code-switching attitudes, and heritage language maintenance), as reliably measured by the validated 19-item CLA questionnaire.
5	Callesano & Carter (2019)	1. "participants perceived the Colombian speaker (5.03) to be more likely than the Cuban (4.44) or Peninsular (4.58) speakers to acquire English in this time. This difference is significant (p=0.000)" (p. 92); 2. "we found that participants rated the Colombian voice (3.96) as the least likely to speak Spanish to his child, below the Cuban voice (4.15) and the Peninsular voice (4.17). This difference is also significant (p=0.03)." (p. 93); 3. "For the Colombian voice, the presence of the Colombian background label increased perception that the speaker would choose to speak Spanish if given a choice." (p. 95); 4. "For the Cuban dialect cells, the presence of any parental background label significantly increased the probability that the speaker was perceived as being worried about losing his Spanish over time... For the Peninsular dialect cells, the labeled conditions significantly decrease the probability that the speaker is perceived as caring about losing Spanish" (p. 94–95)	A(1-2). Dialect-based stereotyping of language behavioral patterns: Latinx listeners systematically associate different Spanish dialects with distinct language behaviors: Colombian Spanish speakers are perceived as more English-oriented (higher likelihood of rapid English acquisition and English-dominant media consumption) and less likely to engage in intergenerational Spanish transmission, while Peninsular and Cuban Spanish speakers are judged more likely to pass Spanish on to their children；B(3-4). Moderating effect of national-origin labels on perceived language behaviors: Parental national-origin background information modulates judgments of speakers' language practices: a Colombian origin label increases the perceived likelihood that a Colombian-accented speaker will select Spanish in bilingual interaction settings; a Cuban origin label amplifies perceived anxiety about Spanish heritage loss for Cuban dialect speakers but reduces such perceived concern for Peninsular Spanish speakers.

No.	Author (Year)	Original Excerpt – Behavioral Dimension	Thematic Summary – Behavioral Dimension
6	Fatima & Nadeem (2025)	1. "Older generations, particularly grandparents and great grandparents, often converse exclusively in their heritage languages and frown upon the use of other languages within the household. Younger generations, in contrast, were reported to either exhibit the use of a mix of primary and heritage languages-frequently code-switching-or favoring culturally dominant languages (e.g., English or Urdu depending on the environment) altogether." (p. 8); 2. "Self-reported primary languages indicated relatively high proficiency (M=3.97, SD=0.983) while heritage language competence was lower (M=2.99, SD=1.144). Communication across generations relied more on dominant/primary languages as indicated by a mean score of 1.84 (SD=0.687)." (p. 11); 3. "Code-switching emerged as a notable practice amongst the younger generations-participants described how the younger generations often alternated between their heritage languages and the culturally dominant language, partly because of limited expertise in heritage languages and partly to navigate social and professional spaces." (p. 8); 4. "family traditions such as festivals, religious ceremonies and even culinary practices required the use of their heritage languages more so than any other dominant language- ensuring that in some ways, the heritage languages remained relevant." (p. 8); 5. "Respondents stipulated extended families to be one of the main contributors toward learning and maintaining their heritage languages, particularly grandparents, aunts, uncles and even cousins. Grandparents were reported to be the most valued when it comes to language maintenance." (p. 8); 6. "participants that had a primarily positive emotional experience with their family when it came to the heritage language would prefer to associate with it more than the participants that had a negative experience." (p. 8); 7. "Dominant languages, like English, are prioritized in educational and professional settings instead of heritage languages... families have now been compelled to focus on teaching English to the younger generations... This has caused the younger generation to also exhibit disinterest and/or resistance toward learning their heritage languages" (p. 10); 8. "A strong positive correlation exists between Language Practices and Ethnic Identity (r=0.628, p<0.01)... A strong positive correlation also exists between Language Practices and Family Dynamics (r=0.610, p<0.01)." (p. 12)	A(1-2). Intergenerational behavioral divergence in language use: Older generations practice exclusive heritage language use in household settings and disapprove of alternative language use; younger generations exhibit a behavioral shift toward dominant languages (Urdu, English), with frequent code-switching or preferential use of dominant languages, resulting in lower heritage language proficiency and dominant-language-dependent intergenerational communication；B(3-4). Context-specific code choice and adaptive code-switching: Heritage languages are functionally restricted to dedicated cultural contexts including festivals, religious ceremonies and traditional practices; younger generations deploy heritage-dominant code-switching as an adaptive strategy for social and professional contexts, partially driven by limited heritage language proficiency；C(5-6). Family-facilitated heritage language maintenance behaviors: Extended family members (notably grandparents) act as core agents of intergenerational heritage language transmission through daily interaction and cultural socialization; individual willingness to use heritage language is directly shaped by the valence of family-associated emotional experiences with the language；D(7-8). Structural pressure-driven heritage language avoidance: Institutional prioritization of dominant languages in education and employment compels families to prioritize dominant language acquisition for youth, generating disinterest and resistance toward heritage language learning; heritage language practice behaviors show significant positive correlations with both ethnic identity strength and family dynamic support.

No.	Author (Year)	Original Excerpt – Behavioral Dimension	Thematic Summary – Behavioral Dimension
7	Gao & Liu (2025)	1. "when minority identity is strong, individuals tend to choose and persist in using their own minority language; conversely, when identity is weak, individuals are more likely to choose other languages." (p. 3); 2. "minority Wa individuals speak only minority Wa language, while minority Lahu individuals are open to the minority Wa language, thus forming the phenomenon of language segregation between minority Wa individuals with a strong minority identity and minority Lahu individuals with a sense of openness to the outside world." (p. 11); 3. "self-minority identity transmission rate has an obvious positive effect on individuals w [who exclusively use their own minority language], both in the ER and the BA networks, especially in the BA network." (p. 10); 4. "increasing the average degree of network, whether they are heterogeneous or homogeneous, can promote minority language to some extent, thereby promoting minority language diffusion." (p. 10); 5. "depending on the degree of minority identity and the status of the language, we obtain different forms of minority languages, namely, language complete extinction, language segregation and language coexistence." (p. 12); 6. "Language coexistence, which indicates the presence of bilingualism in the system, greatly protects language diversity and allows for language coexistence." (p. 12)	A(1-2). Identity-stratified language choice behaviors: Strong minority identity induces exclusive and persistent use of one's own heritage language and rejection of other minority languages, whereas weak minority identity corresponds to behavioral openness to other minority languages; this divergence produces language segregation between co-resident ethnic groups with differing identity strengths；B(3-4). Network-facilitated heritage language diffusion behaviors: Self-minority identity transmission within social networks significantly increases the proportion of individuals who exclusively use their heritage language; heterogeneous scale-free networks are more effective at promoting minority language diffusion than homogeneous random networks, and higher average network connectivity further amplifies heritage language spread；C(5-6). Status-identity interaction yielding three behavioral equilibria: The combination of language status and minority identity strength generates three distinct population-level language behavior patterns: complete language extinction (shift to the higher-status language), language segregation (mutual exclusive heritage language use across groups), and language coexistence (bilingual accommodation sustaining linguistic diversity).

No.	Author (Year)	Original Excerpt – Behavioral Dimension	Thematic Summary – Behavioral Dimension
8	Gavriilidou & Mitits (2021)	1. "While 74.1% (n=40) of respondents said they used Greek predominantly before the age of 5, only 3.7% (n=2) aged 18+ reported the same." (p. 83); 2. "70.3% of the participants reported using both English and Greek, and 16.7% only Greek when speaking to their family members as opposed to merely 13% who use English at home. When asked which language(s) they spoke with their friends, the participants reported the host country language first (n=39, 72.2%), the combination of dominant and HL second (n=13,24.1%), and Greek (N=2,3.7%) third." (p. 83); 3. "the overwhelming majority of participants reported having their family's support in maintaining Greek (98.1%). Similarly, all of them would want to teach their children Greek (100%)." (p. 87); 4. "First-generation HSs... are active members of the Greek community of Chicago, and go to local Greek-orthodox churches every week... actively participate in events organized by local Greek communities and follow all Greek customs and traditions... third-generation children feel mainly American, distance themselves from Greek customs, traditions, and/or cuisine, and very often demonstrate a low proficiency in Greek." (p. 89–90)	A(1-2). Intergenerational language shift and context-dependent code choice: Greek heritage language speakers exhibit a dramatic age-related decline in Greek use, shifting from Greek-dominant use in early childhood to English-dominant use in adulthood; they demonstrate functional code choice partitioning, with Greek and bilingual use prevailing in family interactions while English dominates peer social contexts；B(3). Family-supported heritage language transmission intention: The vast majority of participants receive familial support for Greek language maintenance, and all respondents express a strong intention to transmit Greek to their children；C(4). Identity-coupled cultural-linguistic practice behaviors: First-generation immigrants sustain Greek language use and ethnic identity through active participation in Greek community events, religious observances, and traditional cultural practices; third-generation speakers display reduced Greek proficiency and disengagement from Greek cultural traditions aligned with a predominantly American identity.

No.	Author (Year)	Original Excerpt – Behavioral Dimension	Thematic Summary – Behavioral Dimension
9	Grgič & Guardiancich (2025)	1. "the community's leadership established a minority-language-only environment with a social and economic model focused, among other purposes, on preserving the language and protecting it from assimilation. This strategy narrowed or restricted the network of speakers that became distinctly local, tight and impermeable." (p. 28); 2. "Of course, they [the children in the past decades] all went to Slovenian schools and spoke Slovenian: they knew that they could get a job at TKB [the bank Tržaška kreditna banka], at SAFTI [the financial holding Società anonima finanziaria per i traffici internazionali], in [Slovenian] organisations… Now that's gone and I'm wondering how much longer we will speak Slovenian." (p. 37); 3. "Slovenian speakers in Italy perceive supra-regional standard and non-standard varieties as something foreign, remote, even undesirable and non-adequate, while local varieties, from narrower dialectal to broader regional ones, are considered entirely and exclusively acceptable in all settings" (p. 31); 4. "a regional colloquial variant became widely established, eventually evolving into the only variety in which the speakers were fluent and confident, and which was perceived by them as prestigious and socially acceptable. Contacts with other non-local (supra-regional) standard and non-standard varieties were in fact almost non-existent" (p. 39–40); 5. "the majority language is slowly displacing the minority language in informal communication even among members of the Slovenian community, from family settings to interactions among friends and colleagues" (p. 31); 6. "two ideological cores in particular can be observed among community members: the first-language ideology and the language-pride ideology, both of which serve the exclusivism of the regional standard and the preservation of communication practices or identification options within the local community. The first-language ideology erecting a barrier between Slovenians and Italians and the language-pride ideology raising a barricade between the Slovenians in Italy and those in Slovenia." (p. 41)	A(1-2). Socioeconomically institutionalized exclusive minority language practice: Post-WWII community leaders built a closed, cohesive social-economic system (družbeno gospodarstvo) that enforced a Slovenian-only linguistic environment; community members uniformly used Slovenian across education, employment and daily life, as ethnically bounded job opportunities incentivized heritage language use and created a self-sufficient linguistic community insulated from assimilation；B(3-4). Local-variant exclusive code choice under regional language ideology: Speakers reject supra-regional standard Slovenian varieties as foreign and inadequate, and exclusively adopt and valorize local regional Slovenian variants as the sole acceptable, prestigious form in all settings; linguistic contact with standard Slovenian from the Republic of Slovenia is extremely limited, resulting in stagnated local linguistic innovation；C(5). Informal-domain heritage language shift: The majority Italian language is gradually displacing Slovenian in informal communicative contexts, even in intra-community interactions including family, friends and colleagues；D(6). Dual-boundary identity-based linguistic exclusivism: Community members uphold two interlocking language ideologies: a first-language ideology that constructs a linguistic boundary between the Slovenian minority and Italian majority, and a language-pride ideology that erects a linguistic barrier between local Slovenian speakers and those from the Republic of Slovenia, both reinforcing exclusive use of the local minority standard.

No.	Author (Year)	Original Excerpt – Behavioral Dimension	Thematic Summary – Behavioral Dimension
10	Inan & Harris (2025)	1. "maintaining consistent language policies at home remains challenging, with research showing that only a third of families have explicit rules about language use, and even fewer enforce them regularly" (p. 1); 2. "Participation in bilingual programs has been shown to foster students' cultural identity and linguistic confidence while creating a more equitable learning environment" (p. 2); 3. "children who participate in community-based heritage language schools often demonstrate higher proficiency and stronger cultural identity" (p. 2); 4. "Digital resources such as language learning apps, bilingual storybooks, social media groups, and YouTube channels provide interactive and accessible ways for families to practice their heritage languages outside of formal contexts" (p. 2)	A(1). Constrained household language policy enforcement behavior: Only one-third of immigrant families establish explicit rules governing heritage language use in the home, and an even smaller proportion consistently enforce such policies, limiting the efficacy of family-level language maintenance；B(2). Educational institutional support behaviors for heritage language: Enrollment in bilingual immersion programs and inclusive school curricula promotes students' linguistic confidence and cultural identity, and builds more equitable learning contexts that sustain heritage language use；C(3). Community-based heritage language maintenance behaviors: Community-run heritage language schools and cultural enrichment programs are associated with higher heritage language proficiency and stronger cultural identity among participating children；D(4). Digital platform-enabled heritage language practice behaviors: Families deploy digital tools including language learning apps, bilingual reading materials, social media groups and video channels to carry out heritage language practice in informal, non-institutional settings.

No.	Author (Year)	Original Excerpt – Behavioral Dimension	Thematic Summary – Behavioral Dimension
11	Kovacs Rac & Halupka-Rešetar (2018)	1. "Response (b) was the one that was most often opted for by the research participants (41.23%), i.e. that the dialect is the language nearly everybody speaks in the settlement, the language they learnt from their parents or grandparents." (p. 131); 2. "the next most frequently occurring response was (c), i.e. that the participants knew and liked their dialect most because that is the variety they are used to and that they use most frequently." (p. 131); 3. "approximately half of the respondents claimed to like the dialect spoken in their place of residence, not to feel embarrassed by it and to use it often in public." (p. 132); 4. "Only 10.33% of the pupils (also) stated that they liked their dialect but used it rarely due to fear of being judged." (p. 132); 5. "Approximately two thirds of the respondents (66.67% of the responses...) claimed to use their dialect because that is the language variety they are used to." (p. 134); 6. "Another 22.33% of the responses suggested that speaking in their dialect was a sign of the respondents' love for the town or village where they live." (p. 134); 7. "A total of 37.24% of the respondents reported having been stigmatized in some way because of their dialect or use of language." (p. 137); 8. "as many as 77.24% of the respondents consider the dialect spoken in their place of residence attractive, whereas 22.07% of them claimed that they found it unattractive." (p. 136)	A(1-2). Intergenerational dialect transmission and routine daily use: The local Hungarian dialect functions as the dominant everyday language variety in minority communities, transmitted intergenerationally through parents and grandparents; pupils identify it as the most familiar, preferred and frequently used language form in daily life；B(3-4). Context-stratified public dialect use behaviors: Half of the pupils report frequent, unembarrassed dialect use in public settings, while a smaller subset exhibits restricted public dialect use motivated by fear of social judgment；C(5-6). Habit- and identity-based dialect use motivations: Dialect use behavior is primarily driven by ingrained linguistic habit; for a notable proportion of pupils, dialect speaking serves as a behavioral expression of local identity and affective attachment to their place of residence；D(7-8). Stigma experience and resilient dialect valuation: Over one-third of pupils have encountered mockery or criticism for their dialect use; despite this exposure, a large majority evaluate their local dialect as attractive, indicating sustained positive behavioral orientations toward the heritage variety.

No.	Author (Year)	Original Excerpt – Behavioral Dimension	Thematic Summary – Behavioral Dimension
12	Li (2026)	1. "At home, we basically speak Putonghua and not fangyan […] We can speak fangyan, but our child can't, and we don't want to teach him, because it feels like he's already learning quite a few languages and doesn't need to learn any more. Fangyan is not particularly useful for him anyway. Putonghua is already enough in China […] Between Putonghua and fangyan, I would choose Putonghua, because it can be spoken all over China, whereas fangyan is only used in one place and can't be used elsewhere." (p. 11); 2. "At home we only speak Putonghua, because she no longer understands Wenzhouhua [Wenzhou's fangyan]. Besides, children in China nowadays don't speak Wenzhouhua anymore. Probably only the older generation still does. It will eventually disappear […] Wenzhouhua is very hard for many people to understand. If you're the only one who speaks it, others would find you strange! So, I feel it's unnecessary. Because even in China nobody is learning fangyan anymore, why would you learn it in Italy?" (p. 11–12); 3. "With her, sometimes I speak the local language, sometimes I don't […] She understands it. She can say some things, but not others. Before, when she was little, she was very good at it, speaking Qingtianhua with us all the time! But now, here, she only speaks Putonghua with me." (p. 12); 4. "At home we generally speak our own fangyan with him […] He understands everything, but he can't speak it. He speaks Putonghua with me […] I insist on speaking my hometown language with him: it's rarely used outside, so it's hard to learn. Putonghua can easily be heard outside, can be watched on the phone, so there's no worry about not learning it […] But I still think fangyan is also very important, because after all there are still elderly people. My thinking is a bit traditional: Chinese people are Chinese, after all, and we will certainly return to our roots, whatever happens in the future. So, it's important to learn some of the hometown language as well as Putonghua-both matter." (p. 12–13); 5. "She likes speaking Chinese, so she often speaks it in class […] Even when we discuss the presence of many different ethnicities in the class and emphasise it as a resource, she becomes enthusiastic and starts saying words in Chinese" (p. 13); 6. "She only speaks about Chinese when we encourage her to share a few words with the class. She isn't a child who talks much in general. Even when she does speak, she does so in such a low voice that I can't hear her. When I told her mother about this, she was surprised because she says she speaks very loudly at home! But the other Chinese student does the same." (p. 14); 7. "at school he doesn't speak it at all—it's completely off the table. He doesn't use it even with the other Chinese students he could use it with: either they don't speak to each other, or they speak Italian. It's as if they're in separate compartments: there's a time to speak Chinese and a time to speak Italian" (p. 14)	A(1-2). Standard-language prioritization in family intergenerational transmission: The majority of migrant Chinese parents adopt an exclusive Putonghua-only language policy for parent-child interaction and actively choose not to transmit fangyan; this behavioral choice is justified by perceptions of the limited cross-regional utility of local dialects, their declining vitality in the home country, and concerns about negative interference with children's standard pronunciation；B(3-4). Asymmetric dialect retention and identity-motivated home maintenance: Where parents continue to use fangyan in the household, children develop only passive receptive competence in the local variety while actively producing only Putonghua; one family maintains fangyan as the primary home language driven by traditional identity values, prioritizing intergenerational continuity and connection to ancestral roots as core motivations for dialect transmission；C(5-6). Heterogeneous in-school heritage language behaviors: Children display variable patterns of Chinese language use in school settings: some actively and enthusiastically use Chinese in class when multicultural diversity is framed as a resource, while others only speak Chinese quietly when prompted, or show overall low verbal engagement with the heritage language at school；D(7). Domain-based code compartmentalization: Children exhibit a strict functional separation of linguistic repertoires across social contexts, restricting Chinese (Putonghua) to the home domain and using Italian for school and peer communication; they even avoid using Chinese with co-ethnic peers at school, treating the two languages as mutually exclusive across settings.

No.	Author (Year)	Original Excerpt – Behavioral Dimension	Thematic Summary – Behavioral Dimension
13	Makarova et al. (2019)	1. "with two exceptions, Russian-speaking parents report the use of "only Russian" or "mostly Russian" at home with their children." (p. 468); 2. "children tend to speak the minority language with their siblings at home." (p. 469); 3. "although parents use "Russian only" less frequently with children outside the home than in the home environment, even outside the home, the majority of parents use "mostly" Russian while speaking with their children." (p. 468); 4. "Most parents (17 entries = 57%) indicated that the 'majority' of their friends were Russian-speaking... 24 respondents (80%) answered 'always' when asked how often they communicate in Russian with their friends." (p. 470–471); 5. "most children tend to speak Russian with their friends regularly, and that they also sometimes use some English with them as well." (p. 471); 6. "when they speak in Russian, their children also respond to them in Russian "always" in 14 cases (46.7%); "often" in nine cases (30%)" (p. 470); 7. "less than half of the participants (12 out of 29) answered positively [attending activities in Russian language-related organizations]; nine children attended Russian heritage language school (75%)" (p. 471); 8. "watching Russian TV is the most popular activity for the subject group, followed by listening to Russian songs and listening to parents reading Russian books." (p. 472); 9. "Out of 30 parents' responses to the question whether their child has been to a Russian-speaking country, 25 were positive. Thirteen children visited a Russian-speaking country more than once." (p. 472)	A(1-2). In-home intergenerational heritage language transmission behaviors: Russian-speaking parents adopt a predominantly Russian-only or Russian-dominant code choice for parent-child interaction at home; children also primarily use Russian for sibling communication within the household, sustaining intra-familial heritage language use；B(3-4). Out-of-home language selection and ethnic peer networking behaviors: Parents decrease exclusive Russian use in public contexts but still mostly use Russian when interacting with children outside the home; the majority of parents maintain friendship networks dominated by fellow Russian speakers and consistently use Russian for communication within these networks；C(5-6). Peer interaction and responsive language behaviors: Most children regularly use Russian in communication with Russian-speaking peers, with occasional English mixing; children predominantly reply in Russian when parents address them in the heritage language, with nearly half always responding in Russian；D(7-8). Formal community participation and informal media engagement behaviors: Less than half of the children take part in Russian language-related community activities, with heritage Saturday schools as the primary form; children engage in daily informal Russian language exposure via media channels, among which television viewing is the most prevalent；E(9). Transnational heritage contact behavior: Most children have visited Russian-speaking countries at least once, maintaining direct transnational exposure to the heritage language environment.

No.	Author (Year)	Original Excerpt – Behavioral Dimension	Thematic Summary – Behavioral Dimension
14	Prada (2019)	1. "Growing up in a school with lots of Hispanics I did use Spanglish but I was told by the teachers and other students that couldn't understand Spanish to only use English, so in most of high school I only used English to communicate with my peers." (p. 314); 2. "Before this class, other Spanish professors or Spanish teachers wouldn't allow any use of Spanglish in our classes. They wanted an all-Spanish environment." (p. 314); 3. "my own mother has told me to 'pick one and stick with it,' and to not to switch between" (p. 315); 4. "I hear these types of statements all the time especially from Hispanics; nosotros somos los peores (we are the worst ones). When we hear someone speaks with an accent or use words that are not like ours, we tend to think of ourselves as superior." (p. 315); 5. "this class has definitely made me view my languages in a different way, it has given me a sort of pride or acceptance of my Spanglish. Before, I would feel ashamed of not knowing the language as well as I should but now it's shifted into feeling like 'well, I know how to speak two languages fairly well instead of just knowing how to speak only one'" (p. 317); 6. "Spanglish belongs in the classroom because people think in different ways… this linguistic freedom lets me to work with all of myself instead of sections of myself only." (p. 318); 7. "I do believe that Spanglish belongs in each classroom setting, because there would be more people participating in discussions and answering questions rather than the classmates who still spoke great Spanish." (p. 318); 8. "what is the point of speaking perfect Spanish if you don't let others talk or you're not willing to accept you're wrong?" (p. 319)	A(1-2). Pre-instructional monolingual code compliance behaviors: Spanish heritage speakers previously abided by strict monolingual language policies in academic settings, suppressing Spanglish and code-mixing practices; they restricted their language use to either English in majority-language classrooms or standard Spanish in Spanish-only classrooms in response to institutional norms and teacher/peer pressure；B(3-4). Familial and co-ethnic language policing behaviors: Heritage speakers experience prescriptive language regulation from family members and intra-community peers, who enforce monolingual language norms, criticize mixed-language practices, and express hierarchical judgments of speakers with non-standard or accent-divergent linguistic repertoires；C(5-6). Translanguaging-primed identity-affirming language behaviors: Following exposure to translanguaging pedagogy, students demonstrate shifted behavioral orientations: they embrace Spanglish as a legitimate linguistic practice, report reduced shame around mixed language use, and express increased pride in their full bilingual repertoires；D(7-8). Inclusive academic participation behaviors: Students reconceptualize appropriate academic discourse behaviors, moving from prioritizing standard Spanish purity to prioritizing inclusive participation and mutual communicative engagement; they endorse translanguaging as a mechanism to increase classroom participation among linguistically diverse students.

No.	Author (Year)	Original Excerpt – Behavioral Dimension	Thematic Summary – Behavioral Dimension
15	Shabtaev et al. (2026)	1. "senior generations are the main if not sole consumers of Juhuri poetry and art. MJ youth do not use Juhuri and hardly comprehend it unless previously exposed to it through the presence of grandparents in the home." (p. 623); 2. "Juhuri was preserved only among the first-generation (G1) mothers (often in code-switching with Russian); Russian was spoken among G1 participants and among those migrating as children (G1.5), while younger, second-generation (G2) speakers were basically Hebrew monolinguals, having lost both HLs." (p. 623); 3. "The status of Russian in Israel is polarized between a segment of highly educated immigrants who insist on passing it on to their children and those who prefer to assimilate completely in the country." (p. 623); 4. "Older members of the community do not attribute importance to its maintenance among their children; younger MJs show an interest in the language, but its impracticality in everyday life hinders their enthusiasm about learning it." (p. 623–624); 5. "in Israel, all correlations between EI and language attitudes were significant, ranging from 0.20–0.47 for both languages. In the United States, correlations between EI and language attitudes were significant only for Juhuri, ranging from 0.31–0.44, but not for Russian... the significance of the correlations declines across generations, to the point of no significant correlations between EI and attitudes among G2 participants." (p. 628); 6. "Stronger identity and more positive attitudes are likely to lead to maintenance of HL cultural heritage." (p. 634)	A(1-2). Intergenerational heritage language contraction and shift behaviors in Israel: Juhuri use is restricted to senior generations who consume its cultural products, while Mountain Jewish youth exhibit minimal active use and comprehension of Juhuri without grandparental household exposure. First-generation mothers maintain Juhuri often via code-switching with Russian, G1 and G1.5 speakers use Russian actively, and the second generation shifts to Hebrew monolingualism with loss of both heritage languages；B(3-4). Context-differentiated heritage language maintenance behaviors: Russian maintenance practices in Israel are polarized, with highly educated immigrants enforcing intergenerational Russian transmission while another segment pursues full linguistic assimilation. In the US community, intergenerational Juhuri transmission is de-prioritized by older generations, and younger people’s learning interest is constrained by the language’s limited daily practical utility；C(5-6). Identity-linked behavioral language tendency variations: Ethnic identity significantly correlates with behavioral attitudes toward both heritage languages in Israel, but only with Juhuri in the US, where the Russian-identity correlation vanishes by the second generation. Stronger ethnic identity and more positive language attitudes predict greater practical efforts to maintain heritage language and cultural heritage.

No.	Author (Year)	Original Excerpt – Behavioral Dimension	Thematic Summary – Behavioral Dimension
16	Siebenhütter (2021)	1. "the Kui language is frequently used in speakers' homes and among friends/neighbors/children from their communities. However, the Kui language is used less in more public situations, such as at the market and around outsiders." (p. 9); 2. "Many speakers still use Kui at home and outside of school (e.g. at the market or within their respective communities) and only shift to Thai or other languages when they need to communicate with people from different backgrounds." (p. 18); 3. "100% of the 58–68 age group use Kui at home. However, multilingualism prevails among the younger age groups... Kui is the first language used by children and its use decreases as they age, this is a significant indication that the language of instruction in schools is not Kui" (p. 9–10); 4. "children under the age of 15 no longer speak/understand Kui, and many parents only use Thai with their children to prepare them for school." (p. 16); 5. "50% of the males aged 19–25 described themselves as fluent in the Kui language, followed by the females at 47%... for Thai (29%) females described themselves more often as 'fluent in all situations' than males (25% Thai)... No males in the 19–25 age group described themselves as fluent in all situations [in Lao NET], whereas approximately 22% of the females in the group stated that they were fluent in all situations." (p. 11–12); 6. "one speaker did not mention his Kui background at his workplace in Bangkok, fearing negative consequences... when in public or around people from different linguistic backgrounds, they generally left their 'Kui group' and joined another group. Some hid their ethnic background due to fear of stigmatisation." (p. 18–19); 7. "the majority planned to return to their birthplace in Northeastern Thailand... many speakers still planned to return to their respective villages and use Kui on a daily basis." (p. 20–21); 8. "They agreed that there should be (more) radio/television programmes and books/publications in Kui to help preserve the language and maintain its uniqueness." (p. 17); 9. "approximately 61% of the speakers agreed [that Kui should be written]. One speaker in the 19–25 age group stated that it could be one way to preserve the 'unique language' for future generations." (p. 19)	A(1-2). Domain-specific code choice and situational language shift: Young Kui speakers deploy functional language partitioning, with frequent Kui use in private in-group settings (home, community, peers) and shifts to the dominant Thai language in public cross-group contexts (markets, interactions with outsiders) to meet communicative requirements；B(3-4). Intergenerational Kui attrition and school-mediated parental language adaptation: Home use of Kui declines sharply across generations, from universal practice among older cohorts to reduced use among youth; child Kui proficiency falls with age as Thai-medium schooling expands, and many parents switch to Thai in the home to prepare children for formal education；C(5). Gendered linguistic behavioral orientations: Young female speakers report higher self-rated fluency in Thai and regional Lao across all situational contexts relative to male peers, while males rate their basic Kui fluency marginally higher; these gendered patterns correspond to broader societal gender role structures in Thailand；D(6-7). Stigma management and geographically bounded identity maintenance: Some Kui speakers suppress their ethnic linguistic identity in urban majority-language workplaces to avoid stigma; in public cross-linguistic settings, they disengage from in-group linguistic norms and adopt majority language practices. Most young migrant speakers intend to return to their home villages and resume daily Kui use, tying heritage language practice to their native territorial space；E(8-9). Heritage language revitalization advocacy behaviors: Young Kui speakers support the expansion of Kui-language mass media (radio, television) and printed resources to sustain the language; a majority endorses developing a written standard for Kui as a mechanism to preserve the unique minority language for subsequent generations.

No.	Author (Year)	Original Excerpt – Behavioral Dimension	Thematic Summary – Behavioral Dimension
17	Vari & Tamburelli (2023)	1. "the speech community of Luxembourg created an 'own' linguistically close standard (Standard Luxembourgish), the Belgian speech community relies on a more linguistically distant standard, namely Standard German." (p. 447); 2. "Standard German is the medium of instruction and also a subject in the schools of our Belgian speech community." (p. 460); 3. "Standard Luxembourgish plays only a minor role in the education system... Standard Luxembourgish is not the officially medium of instruction... teachers are advised by the ministry of education to be wary to not discriminate Luxembourgish vernaculars" (p. 460); 4. "Luxembourgish average D-scores were positive (mean = + 0.28), showing a preference for the vernacular over the standard, whereas Belgian participant D-scores were negative (mean = -0.31), indicating a preference for the standard over the vernacular" (p. 458)	A(1). Divergent heritage language standardization behaviors: The two Moselle Franconian speech communities adopt distinct standardization strategies for endangered variety maintenance: Luxembourg implements endogenous standardization by developing a local Standard Luxembourgish linguistically close to vernaculars, while the Belgian German-speaking community adopts exogenous standardization anchored in the more linguistically distant Standard German；B(2-3). Institutional educational language governance behaviors: The Belgian community establishes Standard German as both the official medium of instruction and a formal school subject. In Luxembourg, Standard Luxembourgish has limited institutional status in education, is not an official instructional medium, and educational policies mandate non-discrimination against vernacular varieties in classroom settings；C(4). Standardization-modulated implicit language preference behaviors: The linguistic distance of the standard systematically shapes speakers' implicit behavioral preferences: speakers with a close endogenous standard (Luxembourg) exhibit an implicit preference for the vernacular over the standard, while speakers with a distant exogenous standard (Belgium) show an implicit preference for the standard over the vernacular.

No.	Author (Year)	Original Excerpt – Behavioral Dimension	Thematic Summary – Behavioral Dimension
18	Yiakoumetti (2022)	1. "analysis of teachers' language use revealed that they primarily used students' dominant L1 (English) during lessons. L1 use was employed purposefully and had a number of functions." (p. 5); 2. "All teachers systematically reverted to students' dominant language, English, to introduce and explain new vocabulary and grammatical structures. This L1 function of explanation was the most common function." (p. 5); 3. "teachers utilised students' L1 for all three overarching purposes: instructional (e.g., grammatical and lexical explanations), managerial (e.g., classroom management and feedback provision) and affective (e.g., rapport and care)." (p. 6); 4. "All teachers referred to drawing attention to salient characteristics of Mandarin that differ from English. They explained that this strategy of juxtaposing students' L1 and L2 is beneficial because it provides the metalinguistic awareness that so often facilitates language acquisition and production." (p. 6); 5. "when shown recordings of their language use, they all reported in a number of instances that they were unaware of the fact that they used English (L1)... a sense of surprise and even guilt and regret was conveyed through teachers' discourse as they believed that they should have used students' target language in certain occasions." (p. 7); 6. "they found it difficult to explain new linguistic information in Mandarin due to students' low proficiency in target-language tasks... they struggled similarly to convey meaning in students' dominant language, English, because English was not native for them." (p. 7); 7. "teachers consider the role of family to be at the forefront of successfully maintaining and developing heritage-language competence... grandparents, older relatives such as aunts and uncles, and siblings all help to provide an environment conducive to ethnic-language use" (p. 8); 8. "community-based heritage-language education serves as one of the limited opportunities available for ethnically Chinese children to learn their familial language" (p. 9); 9. "Teachers expressed a desire for training in issues relevant to ethnic-language education... they lacked confidence and felt unprepared for this unique educational context." (p. 8); 10. "sociolinguistically-informed teacher-training programmes that celebrate language diversity, focus on the detection of salient features through crosslinguistic instruction and utilise the benefits to be had from awareness of L1-L2 similarities and differences" (p. 9)	A(1-2). Systematic dominant-language prioritization in heritage language classrooms: Teachers in UK Chinese community schools primarily and purposefully use students' dominant L1 (English) during Mandarin heritage lessons; the most frequent L1 function is introducing and explaining new vocabulary and grammatical structures；B(3-4). Multifunctional cross-linguistic pedagogical behaviors: Teachers deploy students' L1 across three core functional categories—instructional explanation, classroom management, and affective rapport-building; they also implement a contrastive L1-L2 metalinguistic strategy, highlighting structural differences between Mandarin and English to foster metalinguistic awareness and support language acquisition；C(5-6). Unconscious language use and normative conflict behaviors: Teachers often use English in the classroom without conscious awareness, and experience guilt and regret upon noticing such use, as they internalize the norm that target-language-only instruction is expected. Teachers face bidirectional linguistic constraints: limited ability to explain new content in Mandarin due to low student proficiency, and limited ability to elaborate in English due to their own non-native English status；D(7-8). Family-community joint heritage language maintenance behaviors: Family is positioned as the core agent of heritage language competence maintenance, with extended family members (grandparents, aunts, uncles, siblings) creating home environments conducive to ethnic language use; community complementary schools function as a critical institutional channel for ethnic Chinese children to access and learn their familial heritage language；E(9-10). Teacher professional development advocacy behaviors: Heritage language teachers explicitly express demand for specialized training in ethnic language education, reporting low confidence and perceived unpreparedness for the unique educational context; they call for sociolinguistically informed teacher training programmes centred on linguistic diversity, cross-linguistic instruction, and strategic use of L1-L2 similarities and differences.
