# Supplementary material for: Ethnic minorities' language attitudes toward heritage dialects and official standard languages: a narrative review across cognitive, affective, and behavioral dimensions
Source: Front Psychol. 2026 Sep 28;17:1818757. doi: 10.3389/fpsyg.2026.1818757 (PMC13655717; doi:10.3389/fpsyg.2026.1818757)
Supplement: Supplementary file 2 [file Table_2.pdf]

Supplementary Table 2
RQ2 (Part 1) – Interaction Pathway Thematic Summary

Purpose.

This table supports the narrative review titled *Ethnic Minorities' Language Attitudes toward Heritage Dialects and Official Standard Languages: A Narrative Review across Cognitive, Affective, and Behavioral Dimensions.* It presents the aggregated thematic assignments for the first part of Research Question 2 (RQ2): *How do the cognitive, affective, and behavioral dimensions interact with one another?*

Structure.

This document contains three sub-tables, one per attitudinal dimension:

1) **Table D.** Pathway 1: Cognition Shaping Affect (pp. 2-6)
2) **Table E.** Pathway 2: Affect Driving Behavior (pp. 7-11)
3) **Table F.** Pathway 3: Behavior Feeding Back to Cognition and Affect (pp. 12-16)

How to read.

Within each sub-table, every included study that contributed evidence to that pathway is listed with the pathway-specific themes identified from its verbatim excerpts, accompanied by supporting quotations with page numbers. Themes were identified at the study level through inductive coding of extracted evidence; these study-level themes were then aggregated across studies through iterative constant comparison to yield the overarching pathway themes reported in Section 3.3.1 of the main text. The three pathways are recursive: cognitive evaluations shape affective orientations, affective orientations drive behavioral choices, and behavioral practices feed back to reshape both cognition and affect. A study may contribute evidence to one, two, or all three pathways.

Table D. Pathway 1: Cognition Shaping Affect

No.	Author (Year)	Original Excerpt – Cognition Shaping Affect	Thematic Summary – Cognition Shaping Affect
1	Abedini (2025)	1. "English makes me feel more English and reflects my new personality here. I don't feel much interest in 'being Iranian,' so English feels better to me." (p. 11); 2. "Many of us still felt the need to hide our Iranian culture or claim a more accepted background. Because of this, we rarely taught others about our language or traditions, and often said I was Persian rather than Iranian, since many confused it with Parisian... our heritage, culture, and language were often kept private." (p. 12)	A(1). Perceived identity alignment of the dominant language generates positive affect: the cognitive judgment that English corresponds to one's new host-country personality and social context produces stronger positive emotional resonance with English than with Persian；B(2). Perceived social devaluation of heritage culture elicits negative affect: the cognitive assessment that Iranian heritage lacks mainstream social acceptance triggers feelings of shame and affective disengagement from the heritage language.
2	Alsahafi (2026)	1. "Many Hausa speakers have forgotten the language, and some even regard it as a dialect. This has led to a reluctance to speak it within their families..." (p. 3163); 2. "The Hausa language holds limited significance in Saudi Arabia. Learning it might be seen as a waste of time for those uninterested in their African heritage or who find it irrelevant to their professional goals." (p. 3165)	A(1). Cognitive categorization of Hausa as a non-standard dialect reduces affective willingness to use it: the perception that Hausa is merely a dialect rather than a full legitimate language generates reluctance to speak the language in family contexts；B(2). Cognitive judgment of limited practical utility produces affective devaluation: the view that Hausa has minimal societal and professional relevance in the Saudi context leads to the affective assessment that learning the language is a waste of time for individuals without heritage-oriented interest.
3	Baranova (2026)	1. "they're afraid of pronouncing something incorrectly; they're embarrassed that the person next to them might hear and laugh. That's why we have this problem: people are afraid to speak because they think they'll say something wrong and end up in an awkward situation." (p. 10); 2. "I had a classmate who spoke it very well, and at one point she said something that pushed me away from learning Kalmyk for a while: 'Don't speak, you have a terrible Russian accent!'" (p. 12)	A(1). Perceived linguistic inaccuracy generates fear and embarrassment: the cognitive expectation of mispronunciation and error produces affective states of anxiety and shame, inhibiting public speech of Kalmyk among learners；B(2). Critical evaluation of non-standard speech elicits affective disengagement: negative cognitive judgments of accented Kalmyk by fluent speakers trigger feelings of discomfort and withdrawal from language learning among less proficient speakers.

No.	Author (Year)	Original Excerpt – Cognition Shaping Affect	Thematic Summary – Cognition Shaping Affect
4	Beaudrie et al. (2019)	1. "I feel speaking Spanish is less prestigious than speaking other foreign languages such as French." (p. 12); 2. "I don't like it when people codeswitch because it is not a proper way of speaking a language." (p. 13)	A(1). Perceived lower status of the heritage language generates affective devaluation: the cognitive judgment that Spanish carries less social prestige than other foreign languages produces feelings of reduced linguistic worth among heritage learners；B(2). Cognitive framing of code-switching as linguistically invalid elicits negative affect: the belief that mixed-language practices are not a legitimate form of speech generates affective disapproval of code-switching behavior.
5	Callesano & Carter (2019)	1. "Results of the ANOVA of the ratings of the three dialects show that the peninsular Spanish voice was rated significantly more competent as compared to the Colombian and Cuban speakers (p=0.000)." (p. 91); 2. "When asked how physically attractive the speaker was, participants rated the Cuban voice significantly lower than the Colombian and Peninsular voices. This difference in perception is significant (p=0.000)." (p. 92)	A(1). Perceived higher sociolinguistic status of Peninsular Spanish generates positive competence-related affect: listeners' cognitive judgment that European Spanish constitutes a more legitimate standard variety predicts significantly higher ratings of speaker competence traits；B(2). Cognitive framing of Cuban Spanish as lower-status vernacular elicits negative attractiveness affect: the belief that Cuban Spanish is a less prestigious non-standard variety corresponds to significantly lower evaluations of speaker physical attractiveness.
6	Fatima & Nadeem (2025)	1. "I think Punjabi is neither considered a professional language at all. Nor it is associated with people who are very professional. So I think when it comes to societal pressure that was again a very big, contributing factors in me, not trying to learn Punjabi, or speak at it, like, speak it very commonly because it wasn't considered professional or it wasn't being taught in our system as such." (p. 10); 2. "when I was a child, when I was hit by my mother, the exchange was in Siraiki. And it continued and became a language of hatred for me. After that, there have remained stages in my life where whenever I was belittled or discouraged, it was always in Siraiki. Whenever there was violence, it was always Siraiki. So benefits, I don't think there are many." (p. 10)	A(1). Perceived low occupational and institutional status of heritage languages reduces affective motivation for engagement: the cognitive judgment that heritage varieties lack professional legitimacy and educational support generates diminished willingness to learn and use heritage languages；B(2). Cognitive association of heritage language with negative lived events elicits affective aversion: linking the heritage language to experiences of punishment, belittlement, and violence produces feelings of hatred and rejection toward the language.
7	Gao & Liu (2025)	1. "Language status motivates individuals to adopt a dominant or more favourable language for social and economic reasons." (p. 7); 2. "minority identity represents a desire to maintain one's heritage language as a marker of cultural or group identity." (p. 7)	A(1). Perceived language status generates motivational orientation toward adoption: cognitive evaluation of a language's socioeconomic status produces affective motivation to adopt the dominant or more prestigious linguistic variety；B(2). Cognitive recognition of minority group status elicits heritage maintenance desire: awareness of one's ethnic minority position generates affective inclination to preserve the heritage language as a marker of cultural group belonging.

No.	Author (Year)	Original Excerpt – Cognition Shaping Affect	Thematic Summary – Cognition Shaping Affect
8	Gavriilidou & Mitits (2021)	1. "Greek is viewed as a symbol of GHSs' ethnolinguistic identity, culture, and history that has to be preserved and there is a subsequent strong desire, especially from the first and second generation, to maintain it." (p. 89); 2. "since language proficiency in the HL is low for third-generation HSs, the Greek orthodox religion seems to be the defining element of Greek identity for this generation." (p. 90)	A(1). Perceived symbolic role of heritage language generates preservation-oriented affect: the cognitive framing of Greek as a core marker of ethnolinguistic identity, culture, and history produces a strong affective desire for language maintenance among first and second generation speakers；B(2). Perceived insufficient heritage language proficiency reshapes affective identity anchors: the cognitive judgment that heritage language competence is too low to serve as an identity marker among third-generation speakers leads to affective and categorical reorientation toward religious identity as the defining element of Greek belonging.
9	Grgič & Guardiancich (2025)	1. "Slovenian speakers in Italy perceive supra-regional standard and non-standard varieties as something foreign, remote, even undesirable and non-adequate, while local varieties, from narrower dialectal to broader regional ones, are considered entirely and exclusively acceptable in all settings." (p. 31); 2. "speakers' attitude toward their own language is dominated by a sense of pride: according to the speakers interviewed, this is also a characteristic feature of Slovenians in Italy and a strong identity-forming element" (p. 41)	A(1). Cognitive devaluation of non-local linguistic varieties generates affective rejection: the judgment that supra-regional standard and non-standard Slovenian varieties are foreign and inadequate produces feelings of disapproval and rejection toward external language forms；B(2). Cognitive framing of local language as identity core elicits affective pride: the perception that the local Slovenian variety constitutes a central identity marker generates a strong sense of linguistic pride among community members.
10	Inan & Harris (2025)	1. "many mainstream education systems in the United States and other monolingual-oriented countries fail to reflect the linguistic diversity of their student populations... This failure often leads to the marginalization of heritage languages and reinforces assimilationist pressures, which are not healthy for integration of immigrant families." (p. 2); 2. "Schools that adopt inclusive curricula can signal to students and families that their languages and cultures are valued." (p. 2)	A(1). Perceived institutional devaluation of heritage languages generates affective marginalization and assimilation stress: the cognitive judgment that mainstream educational systems do not recognize minority linguistic diversity produces feelings of cultural marginalization and reinforces affective pressures toward assimilation；B(2). Cognitive validation of linguistic diversity in education elicits affective recognition: inclusive curricular policies communicate institutional acceptance of heritage languages, generating feelings of cultural worth and validation among minority families.
11	Kovacs Rac & Halupka-Rešetar (2018)	1. "I like it but I do not use it often because I think that I might be judged." (p. 132); 2. "in this way we express our love for the town or village where we live." (p. 133)	A(1). Anticipated negative social judgment of dialect use elicits affective anxiety: the cognitive expectation that public dialect use will trigger criticism generates feelings of self-consciousness and apprehension；B(2). Cognitive framing of dialect as a local identity marker generates affective attachment: the perception that dialect use signals connection to one's hometown produces feelings of place-based affection and identity bonding.

No.	Author (Year)	Original Excerpt – Cognition Shaping Affect	Thematic Summary – Cognition Shaping Affect
12	Li (2026)	1. "Putonghua can be spoken all over China, whereas fangyan is only used in one place and can't be used elsewhere. Fangyan is not particularly useful for him anyway." (p. 11); 2. "if a child is exposed to it as well, it may lead to some confusion, and Chinese pronunciation may become less standard." (p. 12)	A(1). Perceived limited functional scope of dialects generates affective devaluation: the cognitive judgment that fangyan has narrow geographic utility and little practical value produces feelings of dismissiveness and reduces motivation for dialect transmission；B(2). Perceived interference with standard pronunciation elicits affective concern: the belief that early dialect exposure compromises standard language accuracy generates anxiety about children's linguistic development and discourages dialect use.
13	Makarova et al. (2019)	1. "For a person with Russian-speaking ancestry, it is important to be able to speak Russian" (p. 466); 2. "There is no use in my child's learning Russian in Canada" (p. 467)	A(1). Cognitive linkage of language to ethnic ancestry generates affective identity investment: the judgment that Russian competence is integral to Russian-speaking ancestry produces strong affective commitment to heritage language maintenance；B(2). Perceived lack of instrumental value elicits affective devaluation: the belief that Russian serves no practical purpose in the Canadian context generates dismissive attitudes toward heritage language learning.
14	Prada (2019)	1. "I was very embarrassed speaking to others because my Spanish was not perfect but was expected since I am Hispanic." (p. 314); 2. "the mixing of languages is more for uneducated people" (p. 315)	A(1). Perceived ethnolinguistic expectation elicits embarrassment: the cognitive assumption that Hispanic speakers should command perfect Spanish generates feelings of discomfort when speakers judge their own Spanish as imperfect；B(2). Cognitive linkage of code-mixing to low educational status produces affective devaluation: the belief that language mixing is typical of uneducated populations triggers negative evaluative feelings toward mixed-language linguistic practices.
15	Shabtaev et al. (2026)	1. "in Israel Juhuri is spoken in the shadow of Hebrew's monumental past as a revitalized language and indisputable status as the lingua franca, leading to a gradual decline in positive attitudes across generations" (p. 632); 2. "in the United States, Juhuri holds particular significance as the sole language preserving Jewish heritage for immigrants who have never spoken Hebrew. This uniqueness fosters more positive attitudes, especially in the Affective component among the younger G1.5 and G2 cohorts" (p. 632)	A(1). Perceived dominant status of Hebrew reduces affective valuation of Juhuri in Israel: cognitive recognition of Hebrew's unchallenged position as national lingua franca generates a gradual generational decline in positive affective attitudes toward the heritage language；B(2). Cognitive framing of Juhuri as exclusive heritage carrier strengthens affective attachment in the US: the judgment that Juhuri functions as the unique preserver of Mountain Jewish cultural and religious heritage fosters stronger positive affective attitudes toward the language.

No.	Author (Year)	Original Excerpt – Cognition Shaping Affect	Thematic Summary – Cognition Shaping Affect
16	Siebenhütter (2021)	1. "belonging ‘to the lower end of the hierarchy means stigma and discrimination that the ethnic minority community has to face, socially, ideologically and linguistically’" (p. 19); "one speaker did not mention his Kui background at his workplace in Bangkok, fearing negative consequences." (p. 19); 2. "the older the speakers, the more they believe that the language might be replaced by the national language in all situations" (p. 20); "still more than 30% of [the 19–25 group] believe that such an elimination will occur in the future and that such extinction will be bad." (p. 20)	A(1). Perceived low sociolinguistic status elicits affective stigma and concealment: the cognitive judgment that Kui occupies a low position in the linguistic hierarchy generates fear of negative social consequences, leading speakers to hide their linguistic background in public settings；B(2). Cognitive expectation of language displacement produces affective concern: the belief that Kui may eventually be fully replaced by the national language generates negative affective evaluations of language extinction prospects.
17	Vari & Tamburelli (2023)	1. "Results show that linguistic distance between the standard and its vernaculars can impact on speakers’ attitudes." (p. 448); "the D representing a preference for the vernacular over the standard, were significantly higher in Luxembourg (close standard) than in Belgium (distant standard)." (p. 459); 2. "Standard German, a language whose standardisation is very high and whose status as a language is not contested. Consequently, attitudes towards native speakers of Standard German are shown to be overwhelming positive in contrast to its vernaculars" (p. 460); "Standard Luxembourgish plays only a minor role in the education system... As a result, Standard Luxembourgish lacks prestige" (p. 460)	A(1). Perceived linguistic proximity between standard and vernacular shapes affective valence: cognitive judgments of linguistic distance between a standard variety and its vernaculars determine the degree to which positive evaluations of the standard transfer to the vernacular form；B(2). Cognitive recognition of standard institutional status drives affective prestige: the perceived level of standardisation and institutional embeddedness of the standard generates corresponding levels of affective prestige relative to vernacular varieties.
18	Yiakoumetti (2022)	1. "a teacher felt challenged by the fact that a parent did not wish for her child to be labelled as a speaker of Chinese-as-a-foreign-language." (p. 8); 2. "a sense of surprise and even guilt and regret was conveyed through teachers’ discourse as they believed that they should have used students’ target language in certain occasions." (p. 7)	A(1). Cognitive framing of heritage language as “foreign” elicits affective pushback: categorization of Chinese as a foreign language and its associated labelling generates resistance and dissatisfaction among parents who seek heritage rather than foreign language identification for their children；B(2). Normative cognitive belief in target-language-only instruction elicits affective guilt: internalized norms that instruction should occur exclusively in the target heritage language produce feelings of surprise, guilt, and regret among teachers when they use students’ dominant language.

Table E. Pathway 2: Affect Driving Behavior

No.	Author (Year)	Original Excerpt – Affect Driving Behavior	Thematic Summary – Affect Driving Behavior
1	Abedini (2025)	1. "At our community's cultural fair, I shared Persian stories about Chaharshanbe Suri with friends, switching between Persian and English so everyone could join in. I taught my classmate Liam to say 'sobh bekheyr' (good morning) for fun, and we laughed as he nailed the accent. Mixing languages feels natural and keeps Persian exciting for me." (p. 11); 2. "Honestly, I cared a lot about traditions in Iran, but since migrating, they've become less important to me. Recently, during Chaharshanbe Suri and Nowruz, I had zero interest... Holidays like Christmas, Halloween, and Easter feel more important because everyone celebrates them." (p. 11–12)	A(1). Affective bonding with heritage drives active language engagement: emotional connection to Persian and associated cultural pride motivate behaviors including code-switching in intergroup social settings and active sharing of heritage linguistic content with peers；B(2). Affective distancing from heritage reduces heritage practice: weakened emotional attachment to Iranian traditions and stronger affective investment in host-cultural practices correspond to reduced participation in heritage cultural events and contracted contexts for Persian language use.
2	Alsahafi (2026)	1. "It is crucial for parents and grandparents who speak Hausa to interact with their children and grandchildren in this language at home to prevent it from disappearing from our society." (p. 3164); 2. "There is a need to establish schools and university programmes to teach Hausa in the Kingdom of Saudi Arabia... I wish special schools would be created to teach Hausa and English together." (p. 3164)	A(1). Affective commitment to heritage continuity motivates domestic transmission behaviors: the sense of responsibility to preserve ancestral linguistic roots drives community members to advocate for active intergenerational Hausa use in household settings；B(2). Positive affective valuation of Hausa motivates formal education advocacy behaviors: recognition of the language's cultural and communicative value generates calls for dedicated school and university programmes for Hausa language instruction.
3	Baranova (2026)	1. "When he became a grandfather, he decided that it was necessary to become a Kalmyk speaker. He began reading the newspaper in Kalmyk, Хал'mәg Ünәn, and his special interest is folklore texts or Buddhist rituals and terminology." (p. 9–10); 2. "I want to organize a Kalmyk-language camp in Europe next year. Because kids want to socialize, and parents also want them to socialize with other Kalmyks" (p. 13)	A(1). Intergenerational responsibility motivates intentional heritage language learning: affective commitment to transmitting cultural and linguistic heritage to grandchildren drives older adults to engage in self-directed Kalmyk study and practice；B(2). Diasporic identity attachment drives community language organizing: affective investment in ethnic continuity and youth socialization motivates diaspora activists to establish dedicated heritage language camps for children.

No.	Author (Year)	Original Excerpt – Affect Driving Behavior	Thematic Summary – Affect Driving Behavior
4	Beaudrie et al. (2019)	1. "I would not codeswitch in front of my teachers because they may think I am less intelligent." (p. 13); 2. "I would be willing to join a club who meets every week to discuss how to maintain Spanish within the family and community." (p. 13)	A(1). Anxiety about negative competence evaluation drives restrictive linguistic behaviors: fear of being judged as less intelligent by authority figures motivates learners to suppress code-switching in formal educational settings；B(2). Affective commitment to heritage continuity drives maintenance-oriented behavioral intentions: positive valuation of Spanish language survival motivates willingness to participate in community-based heritage language maintenance organizations.
5	Callesano & Carter (2019)	1. "participants rated the Colombian voice (5.03) to be more likely than the Cuban (4.44) or Peninsular (4.58) speakers to acquire English in this time. This difference is significant (p=0.000)" (p. 92); 2. "participants rated the Colombian voice (3.96) as the least likely to speak Spanish to his child, below the Cuban voice (4.15) and the Peninsular voice (4.17). This difference is also significant (p=0.03)." (p. 93)	A(1). Affective orientation toward dominant society predicts English-acquisition behavior: the perception that Colombian speakers hold stronger affective alignment with English-speaking society corresponds to higher predicted likelihood of English learning behavior；B(2). Weak affective heritage attachment predicts reduced intergenerational transmission behavior: lower emotional bond to Spanish heritage among Colombian communities is associated with lower expected likelihood of intergenerational Spanish language transmission.
6	Fatima & Nadeem (2025)	1. "A strong positive correlation exists between Language Practices and Ethnic Identity (r=0.628) illustrating higher levels of engaging in heritage language use and practice is often associated directly with stronger ethnic identity and ethnic attachment." (p. 12); 2. "Participants with positive experiences associated to their heritage languages leaned more toward trying to preserve it, while those with negative experiences and emotions associated to their heritage languages were less caring toward the idea of maintaining their language." (p. 10)	A(1). Strong ethnic identity attachment drives increased heritage language practice: affective bonding to ethnic identity correlates significantly with higher frequency of heritage language use and engagement in maintenance behaviors；B(2). Valenced emotional experiences predict heritage maintenance behaviors: positive affective associations with heritage languages motivate active preservation efforts, while negative emotional experiences reduce behavioral investment in language maintenance.
7	Gao & Liu (2025)	1. "According to the survey results, minority Wa individuals speak only minority Wa language, while minority Lahu individuals are open to the minority Wa language, thus forming the phenomenon of language segregation between minority Wa individuals with a strong minority identity and minority Lahu individuals with a sense of openness to the outside world." (p. 11); 2. "the self-minority identity transmission rate has an obvious positive effect on individuals w, both in the ER and the BA networks, especially in the BA network." (p. 10)	A(1). Strength of identity attachment shapes language use and segregation patterns: strong minority identity alignment drives exclusive heritage language use and linguistic separation from out-groups, while weaker identity and greater openness correspond to willingness to adopt other minority languages；B(2). Identity propagation dynamics increase exclusive heritage language practice: higher rates of self-minority identity transmission across social networks expand the share of speakers who use only their own minority language.

No.	Author (Year)	Original Excerpt – Affect Driving Behavior	Thematic Summary – Affect Driving Behavior
8	Gavriilidou & Mitits (2021)	1. "First-generation HSs demonstrate high proficiency in Greek and claim they are Greek because they were born and raised in Greece. They relate to Greek more than to American culture, are active members of the Greek community of Chicago, and go to local Greek-orthodox churches every week... They also actively participate in events organized by local Greek communities and follow all Greek customs and traditions." (p. 89); 2. "they wish to preserve the Greek identity of their children by sending them to community-based schools" (p. 89)	A(1). Strong ethnocultural attachment drives active heritage community participation: affective identification with Greek culture and sense of belonging motivate engagement in Greek community activities, Orthodox religious practice, and adherence to cultural traditions；B(2). Affective commitment to intergenerational identity continuity drives heritage education behaviors: the desire to preserve Greek ethnic identity for the next generation motivates parental decisions to enroll children in community-based Greek language schools.
9	Grgič & Guardiancich (2025)	1. "the first-language ideology erecting a barrier between Slovenians and Italians and the language-pride ideology raising a barricade between the Slovenians in Italy and those in Slovenia." (p. 41); 2. "community's leadership established a minority-language-only environment with a social and economic model focused, among other purposes, on preserving the language and protecting it from assimilation. This strategy narrowed or restricted the network of speakers that became distinctly local, tight and impermeable." (p. 28)	A(1). Identity-protective affective orientations drive linguistic exclusion behaviors: dual ideological commitments (first-language ideology vis-à-vis the majority, language-pride ideology vis-à-vis the homeland) motivate boundary-maintaining behaviors that separate local speakers from both Italian majority groups and Slovenian homeland populations；B(2). Anti-assimilation affective priorities drive restrictive network governance: the desire to protect the minority language from assimilation motivates community leaders to construct a closed, tight-knit social and economic network that enforces exclusive minority language use.
10	Inan & Harris (2025)	1. "Some families view bilingualism as a socioeconomic asset that can enhance career prospects and global mobility, motivating them to seek out both community and digital resources" (p. 2); 2. "societal attitudes and employment concerns lead many parents to deprioritize heritage language use" (p. 2)	A(1). Instrumental affective valuation of bilingualism drives resource-seeking behaviors: the perceived socioeconomic benefit of bilingualism motivates families to pursue community-based and digital heritage language support resources；B(2). Affective concerns about socioeconomic consequences drive reduced heritage language prioritization: worries about employment prospects and perceived societal attitudes motivate parents to lower the priority of heritage language use in household settings.
11	Kovacs Rac & Halupka-Rešetar (2018)	1. "I like it very much, I do not feel embarrassed by it and use it often in public." (p. 131); 2. "I like it but I do not use it often because I think that I might be judged." (p. 132)	A(1). Positive affective regard for dialect motivates public language use: liking for the local variety and absence of embarrassment correspond with frequent public deployment of the dialect；B(2). Fear of negative evaluation motivates restricted dialect use: concern about social judgment suppresses public dialect use even among speakers who hold positive personal attitudes toward the variety.

No.	Author (Year)	Original Excerpt – Affect Driving Behavior	Thematic Summary – Affect Driving Behavior
12	Li (2026)	1. "I still think fangyan is also very important, because after all there are still elderly people. My thinking is a bit traditional: Chinese people are Chinese, after all, and we will certainly return to our roots. So, it's important to learn some of the hometown language as well as Putonghua-both matter." (p. 13); 2. "we don't want to teach him, because it feels like he's already learning quite a few languages and doesn't need to learn any more. Fangyan is not particularly useful for him anyway." (p. 11)	A(1). Affective valuation of intergenerational and roots-based identity drives dialect maintenance: emotional attachment to family continuity and traditional cultural belonging motivates parents to use fangyan in the home and prioritize its intergenerational transmission；B(2). Instrumental affective prioritization of standard language drives dialect shift: perceived practical benefits of Putonghua and concerns about excessive linguistic load motivate families to adopt exclusive standard-language home practices and marginalize dialect use.
13	Makarova et al. (2019)	1. "They show no desire to be 'assimilated,' and want Russian language maintained in the family." (p. 465); 2. "more positive language attitudes are likely to drive parents to create a richer minority language environment" (p. 473)	A(1). Affective rejection of assimilation drives domestic heritage maintenance: resistance to cultural assimilation motivates families to sustain Russian language use within household settings；B(2). Positive affective orientations toward heritage language drive expanded input behaviors: favorable parental attitudes toward Russian motivate active efforts to construct rich home and community linguistic environments for children.
14	Prada (2019)	1. "Growing up in a school with lots of Hispanics I did use Spanglish but I was told by the teachers and other students that couldn't understand Spanish to only use English, so in most of high school I only used English to communicate with my peers." (p. 314); 2. "I shouldn't keep my Spanish my secret language" (p. 317)	A(1). Fear of social judgment drives restrictive language choice: anxiety about negative evaluation from teachers and majority peers motivates speakers to suppress mixed-language use and adopt exclusive English communication in school settings；B(2). Affective acceptance of linguistic repertoire drives open language deployment: increased pride in and identification with one's bilingual practices motivates willingness to publicly use the full range of one's linguistic repertoire.
15	Shabtaev et al. (2026)	1. "Stronger identity and more positive attitudes are likely to encourage speakers to put practical efforts into maintaining their HL." (p. 634); 2. "self-proclaimed practical efforts of potential speakers with high EI to maintain it (as seen in the higher results of the Behavioral component)" (p. 633)	A(1). Strong affective ethnic identity and positive attitudes drive heritage maintenance behaviors: greater ethnic commitment and favorable affective orientations toward heritage languages correspond with increased practical engagement in language preservation efforts；B(2). High ethnic identity intensity predicts greater behavioral investment in heritage maintenance: individuals with stronger ethnic identity exhibit higher levels of behavioral intention and effort to sustain heritage language use.

No.	Author (Year)	Original Excerpt – Affect Driving Behavior	Thematic Summary – Affect Driving Behavior
16	Siebenhütter (2021)	1. "young Kui speakers still have a deep connection to their cultural heritage and linguistic background… the prestige of Thai is not a strong enough reason for young Kui speakers to give up their minority language altogether." (p. 20); "many speakers still planned to return to their respective villages and use Kui on a daily basis" (p. 21); 2. "Young speakers used Thai outside their homes to fit in and be easily understood by others." (p. 19); "shame to be a member of a minority group and the hope to gain a better reputation by using Thai (language prestige)" (p. 21)	A(1). Affective heritage attachment drives language maintenance behaviors: strong emotional connection to Kui cultural and linguistic heritage motivates continued home and community use of the language and intentions to return to origin communities for daily linguistic practice；B(2). Affective concerns about social acceptance drive language shift behaviors: feelings of minority shame and desire for improved social reputation motivate speakers to adopt Thai in public and intergroup contexts to facilitate integration.
17	Vari & Tamburelli (2023)	1. "Language maintenance research generally argues that providing endangered varieties with a standard impacts positively their vitality by e.g. increasing positive attitudes." (p. 448); 2. "standardisation of endangered vernaculars in order to improve attitudes and thus bolster their position in language contact situations" (p. 459)	A(1). Positive affective evaluations enhance heritage language vitality: increased positive attitudes toward endangered vernacular varieties strengthen their overall viability in language contact contexts；B(2). Affective attitude improvement supports maintenance-oriented outcomes: interventions targeting attitudinal enhancement are deployed as a mechanism to bolster the position of endangered minority languages.
18	Yiakoumetti (2022)	1. "In their efforts to build rapport and assist students with personal matters... teachers purposefully employed students' first language. They elaborated that their choices were driven by their beliefs that students associated the second language with teaching and the first language with more familiar, casual and comfortable settings." (p. 6); 2. "The parents are motivated. They want their children to speak Chinese and to learn about Chinese culture." (p. 8)	A(1). Affective comfort and rapport goals drive L1-mediated pedagogical behavior: the sense that the dominant language is tied to familiar, casual settings motivates teachers to use English to build interpersonal rapport and support students' personal needs in the heritage language classroom；B(2). Affective valuation of cultural and linguistic heritage drives enrollment behavior: parental motivation to maintain Chinese language and cultural connection motivates decisions to enroll children in community-based heritage language schools.

Table F. Pathway 3: Behavior Feeding Back to Cognition and Affect

No.	Author (Year)	Original Excerpt – Behavior Feeding Back to Cognition & Affect	Thematic Summary – Behavior Feeding Back to Cognition & Affect
1	Abedini (2025)	1. "these everyday interactions in Persian deepen her sense of belonging and reinforce her intercultural identity, showing how maintaining Persian and negotiating identity continually shape one another." (p. 9); 2. "the use of Persian in cultural practices, such as teaching Persian phrases to friends and sharing Persian in community or peer settings, shaped participants' intercultural identity, reinforcing cultural pride and hybrid belonging." (p. 13)	A(1). Routine heritage language use strengthens affective belonging and identity cognition: regular Persian use in family interactions deepens feelings of cultural closeness and reinforces individuals' cognitive framing of intercultural identity；B(2). Integrated bilingual practices reshape identity cognition and affective investment: active deployment of Persian within mixed cultural contexts enhances cognitive recognition of hybrid cultural belonging and amplifies affective investment in the heritage language.
2	Alsahafi (2026)	1. "A moderate positive correlation was observed between Hausa proficiency and the perceived importance of speaking the language ($r=.32$, $p<0.01$)." (p. 3163); 2. "Individuals who self-report higher proficiency in Hausa are more likely to acknowledge its cultural significance and promote passing it on to future generations." (p. 3166)	A(1). Heritage language proficiency behaviorally reinforces cognitive judgments of importance: higher self-rated Hausa proficiency is positively associated with the cognitive perception that speaking the language carries importance；B(2). Active language engagement strengthens cognitive recognition of cultural value and affective commitment to transmission: greater practical engagement with Hausa increases acknowledgment of its cultural significance and enhances motivation to support intergenerational language transfer.
3	Baranova (2026)	1. "My oldest son, he's in Canada. And he says, like, we're getting together, the Kalmyk diaspora, and they even sing Kalmyk songs in restaurants, play the dombra, he took the dombra over there. And he said, Mom, somehow this Kalmyk inside me, it's just coming out." (p. 12); 2. "These developments contribute to the emergence of a market for private tutoring, commercial courses, and other signs of the 'commodification' of language... This may strengthen the sustainability of revitalization efforts by increasing the visibility of the language and reshaping language attitudes." (p. 14)	A(1). Collective heritage language practice strengthens internal affective identity: active participation in diasporic Kalmyk cultural and linguistic activities intensifies feelings of inherent ethnic-linguistic belonging and reinforces self-categorization as a Kalmyk speaker；B(2). Expanded language learning practices reshape community attitudes toward the language: the growth of formal and commercial language learning opportunities increases the visibility of Kalmyk and positively shifts community perceptions of its value and revitalization potential.

No.	Author (Year)	Original Excerpt – Behavior Feeding Back to Cognition & Affect	Thematic Summary – Behavior Feeding Back to Cognition & Affect
4	Beaudrie et al. (2019)	1. "Paired-sample t-tests performed on the pretest and posttest scores showed significant post-instruction changes for factor 1 [t(18)=-2.38, p<.05], factor 2 [t(18)=-2.18, p<.05], and factor 3 [t(18)=-3.25, p<.05]." (p. 11); 2. "becoming critically language aware entails learners becoming cognizant of the naturalness of language variation and its loading of social, political, and economic power structures, while accepting the intrinsic value of their own bilingual varieties and all others." (p. 15)	A(1). Critical language awareness instruction produces measurable cross-dimensional change: structured CLA intervention generates significant positive shifts across three attitudinal and behavioral domains: linguistic prejudice, code-switching attitudes, and heritage language maintenance intentions；B(2). Sustained critical learning practices reshape cognitive and affective orientations: engagement with CLA curricula increases cognitive understanding of language variation as a natural phenomenon and enhances affective acceptance of learners' own bilingual linguistic repertoires.
5	Callesano & Carter (2019)	/	/
6	Fatima & Nadeem (2025)	1. "A moderate positive correlation is observed between Language Practices and Attitudes and Perceptions (r=0.365), advocating that those who positively engage in heritage language practices tend to have more positive attitudes and perceptions about heritage language maintenance as well." (p. 12); 2. "positive reinforcement and encouraging experiences with heritage language often result in positive attitudes toward heritage language maintenance and transmission." (p. 14)	A(1). Active heritage language practice reinforces positive cognitive evaluations of maintenance: regular engagement with heritage language use correlates with more favorable cognitive judgments of the value and importance of heritage language preservation；B(2). Supportive language interaction behaviors strengthen affective and cognitive orientations toward heritage: encouraging interpersonal experiences with heritage languages produce more positive attitudes toward both the language itself and its intergenerational transmission.
7	Gao & Liu (2025)	1. "self-minority identity transmission can contribute to the preservation of minority languages by increasing individual minority identity." (p. 12); 2. "Improving the average degree of network, whether heterogeneous or homogeneous, can promote the development of minority languages to some extent, and thus promote the diffusion of minority languages." (p. 12)	A(1). Identity transmission behaviors reinforce individual minority identity: active engagement with minority identity propagation strengthens both cognitive recognition of minority status and affective commitment to heritage language preservation；B(2). Expanded network interaction enhances perceived minority language viability: increased social connectivity and language interaction within networks improves cognitive assessments of minority language development potential and supports further linguistic diffusion.

No.	Author (Year)	Original Excerpt – Behavior Feeding Back to Cognition & Affect	Thematic Summary – Behavior Feeding Back to Cognition & Affect
8	Gavriilidou & Mitits (2021)	1. "the more their parents have engaged them in cultural and social activities associated with the Greek community and culture, the higher sense of appreciation for Greek language and culture the children have." (p. 89); 2. "in cases where second-generation parents did not invest in the preservation of Greek culture... third-generation children feel mainly American, distance themselves from Greek customs, traditions, and/or cuisine, and very often demonstrate a low proficiency in Greek." (p. 89–90)	A(1). Sustained cultural engagement behaviors enhance affective valuation of heritage language: parental engagement of children in Greek community and cultural activities increases children's affective sense of appreciation for both the Greek language and culture；B(2). Absence of heritage cultural investment behaviors erodes identity and language outcomes: lack of parental investment in Greek cultural preservation leads third-generation children to develop stronger American affective identification, greater cognitive distance from Greek traditions, and lower heritage language proficiency.
9	Grgič & Guardiancich (2025)	1. "the genesis, development and entrenchment of a local-language ideology, which encourages the exclusive use of the local standard, can be attributed to a greater extent to social dynamics stimulated by the economic model of the so-called družbeno gospodarstvo." (p. 42); 2. "Now that's gone and I'm wondering how much longer we will speak Slovenian." (p. 38)	A(1). Sustained closed-network language practices entrench local-language ideology: long-term operation of a self-contained social and linguistic system reinforces cognitive beliefs in the legitimacy of the local standard and solidifies exclusive linguistic norms；B(2). Erosion of supporting socioeconomic practices reshapes cognitive expectations of language survival: the collapse of the minority social economy produces pessimistic cognitive assessments of future heritage language viability and generates affective anxiety about language loss.
10	Inan & Harris (2025)	1. "Participation in bilingual programs has been shown to foster students' cultural identity and linguistic confidence while creating a more equitable learning environment" (p. 2); 2. "children who participate in community-based heritage language schools often demonstrate higher proficiency and stronger cultural identity" (p. 2)	A(1). Bilingual program engagement strengthens affective identity and cognitive linguistic confidence: participation in dual language education enhances affective sense of cultural belonging and increases cognitive confidence in heritage language ability among students；B(2). Community heritage language practice reinforces cognitive proficiency and affective identity attachment: engagement in community-based heritage language instruction improves cognitive linguistic proficiency and strengthens affective cultural identity among children.
11	Kovacs Rac & Halupka-Rešetar (2018)	1. "the dialect is the language we know best and like most because we are used to it and the language we use most often." (p. 130); 2. "A total of 37.24% of the respondents reported having been stigmatized in some way because of their dialect or use of language. In spite of this, as many as 77.24% considered their dialect attractive." (p. 138)	A(1). Routine dialect use strengthens cognitive familiarity and affective preference: regular daily engagement with the local variety increases perceived competence and generates greater liking for the dialect；B(2). Experiences of dialect stigmatization do not diminish positive cognitive and affective evaluations: despite encountering negative reactions to their speech, most speakers maintain favorable judgments of and attachment to their local dialect.

No.	Author (Year)	Original Excerpt – Behavior Feeding Back to Cognition & Affect	Thematic Summary – Behavior Feeding Back to Cognition & Affect
12	Li (2026)	1. "it's as if they make a very clear distinction between the two languages, and Chinese tends to predominate, because they speak it at home and attend the other school, so there is also institutional recognition […] It's as if Chinese were their language, while Italian is just a means they need to learn to communicate with their classmates." (p. 15); 2. "She likes speaking Chinese, so she often speaks it in class […] Even when we discuss the presence of many different ethnicities in the class and emphasise it as a resource, she becomes enthusiastic and starts saying words in Chinese" (p. 14)	A(1). Context-segregated language practices reinforce functional cognitive framing of languages: consistent separation of Chinese (home/heritage) and Italian (school/communicative) across domains strengthens the cognitive classification of heritage language as identity-based and majority language as instrumental；B(2). Positive public language use experiences enhance affective engagement with heritage language: opportunities to use Chinese in inclusive school settings increase children's emotional enthusiasm for and identification with their heritage language.
13	Makarova et al. (2019)	1. "the more parents speak Russian at home, the fewer calques occur in children's speech." (p. 468); 2. "Children who engage in activities conducted in Russian language-related organizations are more likely to make fewer mistakes and use fewer English loan words when speaking Russian." (p. 471)	A(1). Sustained home language practice strengthens cognitive linguistic accuracy: regular parental Russian use at home reduces calque errors in children's speech, reinforcing cognitive perceptions of heritage language competence；B(2). Community language activity participation enhances cognitive linguistic performance: engagement with organized Russian language activities reduces error rates and loanword use, strengthening both cognitive proficiency judgments and practical linguistic outcomes.
14	Prada (2019)	1. "this class has definitely made me view my languages in a different way, it has given me a sort of pride or acceptance of my Spanglish." (p. 317); 2. "I learned that that there is no set perfect or ideal way to speak Spanish. The different cultures from where we are and our upbringings all vary . . . and these help form the language we speak." (p. 317)	A(1). Classroom translanguaging practice strengthens affective acceptance of stigmatized varieties: sustained engagement with flexible linguistic practices in the instructional setting increases feelings of pride and acceptance of historically marginalized mixed-language forms；B(2). Translanguaging participation reshapes cognitive beliefs about linguistic standard: exposure to translanguaging pedagogy challenges the notion of a single ideal standard Spanish and enhances cognitive recognition of linguistic diversity as normative.
15	Shabtaev et al. (2026)	1. "Ongoing immigration contributes to the increasing number of Russian heritage speakers in the United States, and, along with the absence of a shared ethnolinguistic identity which would encourage abandoning a HL, further solidifies its significance" (p. 633); 2. "in Israel attitudes toward Juhuri and Russian decline across generations for all three attitudinal components" (p. 627)	A(1). Expanded community language practices reinforce cognitive perceptions of heritage language significance: sustained growth of Russian-speaking communities and ongoing immigration strengthen cognitive judgments of the language's sociocultural value and importance；B(2). Diminished intergenerational heritage language use erodes positive cognitive and affective attitudes: reduced heritage language practice across successive generations corresponds with declining evaluative orientations toward both heritage languages in the Israeli context.

No.	Author (Year)	Original Excerpt – Behavior Feeding Back to Cognition & Affect	Thematic Summary – Behavior Feeding Back to Cognition & Affect
16	Siebenhütter (2021)	1. "Kui language usage has decreased. For instance, 100% of the 58–68 age group and only 40% of the 15–25 age group use Kui at home." (p. 18); "the younger the speakers are, the more they speak Thai, also at home. This indicates a generational patterning" (p. 17); 2. "young Kui speakers were flexible in their use and attitude toward the Kui language. In fact, the young speakers were open to maintaining their multilingualism in many situations." (p. 22)	A(1). Diminished intergenerational language use reshapes cognitive expectations of viability: declining rates of Kui use across successive generations reinforces cognitive perceptions that the language is threatened and vulnerable to extinction；B(2). Sustained contextual multilingual practice fosters flexible cognitive and affective orientations: regular deployment of both Kui and Thai across different social contexts produces open attitudes toward multilingualism and flexible linguistic repertoires.
17	Vari & Tamburelli (2023)	1. "speech community was the strongest of all predictor variables, suggesting that how endangered vernaculars are standardised influences attitudes towards them more than any other sociolinguistic factor such as age or gender." (p. 459); 2. "only the introduction of a close standard can lead to the intended protective effect. When a distant standard is introduced, we face a situation where only the standard itself will potentially be maintained, to the detriment of the endangered vernaculars themselves" (p. 459)	A(1). Standardisation practices reshape affective attitudes toward vernaculars: the type of standardisation strategy (endogenous linguistically close vs. exogenous linguistically distant) is the strongest predictor of affective evaluations of endangered vernacular varieties；B(2). Standardization behaviors alter cognitive perceptions of vernacular legitimacy: adoption of a proximate standard reinforces cognitive recognition of the vernacular as a valid variety, while reliance on a distant standard undermines its perceived linguistic value.
18	Yiakoumetti (2022)	1. "Teachers employed students' dominant language as a successful pedagogical strategy. L1 use was natural and served pedagogical, interpersonal and communicative purposes. Harnessing students' L1 is thus viewed as a pedagogical tool that could help maintain and develop diversity." (p. 9); 2. "If teachers are more sociolinguistically informed about the language habits of students who attend complementary schools, they may well be in a better position to make and justify language choices that are suited to their specific environment... teachers lack confidence and felt unprepared for this unique educational context." (p. 8)	A(1). Sustained L1-integrated teaching practice reinforces cognitive recognition of L1 as a valid pedagogical resource: systematic use of students' dominant language in heritage instruction strengthens the cognitive framing of L1 as a functional, valuable tool for supporting heritage language learning and linguistic diversity；B(2). Targeted sociolinguistic training behaviors enhance teacher confidence and cognitive competence: provision of context-specific training improves teachers' affective sense of preparedness and strengthens their cognitive ability to make appropriate, justifiable language choices in the heritage classroom.
