# Supplementary material for: Ethnic minorities' language attitudes toward heritage dialects and official standard languages: a narrative review across cognitive, affective, and behavioral dimensions
Source: Front Psychol. 2026 Sep 28;17:1818757. doi: 10.3389/fpsyg.2026.1818757 (PMC13655717; doi:10.3389/fpsyg.2026.1818757)
Supplement: Supplementary file 3 [file Table_3.pdf]

Supplementary Table 3
RQ2 (Part 2) – Stable Attitudinal Pattern Coding

Purpose.

This table supports the narrative review titled *Ethnic Minorities' Language Attitudes toward Heritage Dialects and Official Standard Languages: A Narrative Review across Cognitive, Affective, and Behavioral Dimensions.* It presents the full pattern-coding output for the second part of Research Question 2 (RQ2): *How do the three dimensions interact to form stable attitudinal patterns such as structured ambivalence?*

Structure.

This document contains one sub-table:

1) **Table G.** Stable Attitudinal Pattern Coding - Full Table with Verbatim Evidence and Assignment Rationale (N = 34 Analytical Units) (pp. 2-16)

How to read.

Each row represents one analytical unit—a distinguishable subgroup, generation, or national context within a single study that exhibits a coherent attitudinal configuration. The six columns are:

1) **No.** – Consecutive numbering with letter suffixes for multiple units from the same study (e.g., 1a, 1b).
2) **Author (Year)** – Full author citation.
3) **Analytical Unit** – Brief description of the subgroup or context.
4) **Pattern Assigned** – One of: Assimilation, Resistance, Structured Ambivalence, Structured Ambivalence (domain-differentiated subtype), Transition, or Partial evidence.
5) **Original Excerpt – Pattern Evidence** – 2–3 verbatim quotations demonstrating the cognitive, affective, and behavioral components, each ending with (p. XX).
6) **Thematic Summary – Pattern Rationale** – Concise explanation of the pattern assignment, covering all four components (cognitive, affective, behavioral, feedback direction).

Coding rules.

A pattern was assigned only when verbatim evidence existed for at least one component in each of the three dimensions plus an identifiable feedback direction. Units with evidence from fewer than three dimensions were marked as "Partial evidence." Units documenting explicit pre/post intervention change were coded as "Transition" processes rather than stable patterns. The complete coding protocol is described in Section 2.4 of the main text.

Table G. Stable Attitudinal Pattern Coding - Full Table with Verbatim Evidence and Assignment Rationale (N = 34 Analytical Units)

No.	Author (Year)	Analytical Unit	Pattern Assigned	Original Excerpt – Pattern Evidence	Thematic Summary – Pattern Rationale
1a	Abedini (2025)	Participants demonstrating emotional connection to Persian (Parmida, Zoha, Sofia, Ali)	Resistance	"Persian is my mother tongue. When I speak Persian at home, I feel like I'm closer to my family... Persian feels more emotional and familiar. It's like a thread that ties us together at home." (p. 9); "Recently when I joined Instagram and would do lives with my Iranian friend once in a while, speaking Farsi made me proud." (p. 9); "these everyday interactions in Persian deepen her sense of belonging and reinforce her intercultural identity, showing how maintaining Persian and negotiating identity continually shape one another." (p. 9)	Cognitively, Persian is framed as the mother tongue and an emotional anchor tied to family and Iranian heritage. Affectively, participants express pride, belonging, and emotional closeness. Behaviorally, they actively use Persian in family interactions, social media live streams, peer teaching, and cultural celebrations. Feedback is positive: routine Persian interactions deepen belonging and reinforce intercultural identity, motivating further use. This matches the Resistance pattern: cognitive revaluation, consistent positive affect, behavioral expansion, and a positive feedback cycle.
1b	Abedini (2025)	Participants demonstrating cultural integration through bilingual practice (Gelareh, Shayan, Behrad)	Structured Ambivalence (domain-differentiated subtype)	"I don't really think of myself as just Iranian – I'm an Iranian American... speaking Persian is an important way I stay connected to my roots." (p. 10); "We still do all the Persian traditions in our household such as Nowruz... We also celebrate Christmas, put up a Christmas tree, and observe Thanksgiving, so we combine both traditions together." (p. 11); "At our community's cultural fair, I shared Persian stories about Chaharshanbe Suri with friends, switching between Persian and English so everyone could join in." (p. 11)	Cognitively, participants simultaneously endorse Persian's heritage value ("connected to my roots") and English/host culture's practical relevance, self-identifying as "Iranian American." Affectively, they exhibit hybrid belonging to both cultures without explicit internal contradiction. Behaviorally, they demonstrate domain-differentiated code choice: Persian in family/community contexts, English in public/school settings, natural code-switching at intercultural events, and blended cultural traditions. Feedback reinforces hybrid belonging rather than resolving toward either pole. This matches the domain-differentiated subtype of Structured Ambivalence.
1c	Abedini (2025)	Participants demonstrating emotional distance from Persian and preference for English (Elena, Sina)	Assimilation	"English makes me feel more English and reflects my new personality here. I don't feel much interest in 'being Iranian,' so English feels better to me." (p. 11); "Honestly, I cared a lot about traditions in Iran, but since migrating, they've become less important to me. Recently, during Chaharshanbe Suri and Nowruz, I had zero interest... Holidays like Christmas, Halloween, and Easter feel more important because everyone celebrates them." (p. 11–12); "assimilation pressures and comfort with English sometimes weakened attachment to Persian." (Abstract)	Cognitively, English is framed as the carrier of a new identity ("reflects my new personality"), while Persian is judged as lacking emotional value and cultural relevance. Affectively, participants show consistent disidentification with Iranian heritage ("zero interest," "don't feel much interest in being Iranian") and strong identification with English/host culture. Behaviorally, they withdraw from Persian cultural traditions (no participation in Nowruz/Chaharshanbe Suri) and adopt host-culture holidays, contracting Persian use domains. Feedback is negative: assimilation pressures and English comfort further weaken attachment to Persian, reducing use contexts. This matches the Assimilation pattern.

No.	Author (Year)	Analytical Unit	Pattern Assigned	Original Excerpt – Pattern Evidence	Thematic Summary – Pattern Rationale
1d	Abedini (2025)	Participant demonstrating simultaneous pride and shame toward Iranian heritage (Leila)	Structured Ambivalence	"Even though I was proud of my culture and its deep traditions, I sometimes felt ashamed to be associated with it, especially because of negative stereotypes and misunderstandings." (p. 12); "Many of us still felt the need to hide our Iranian culture or claim a more accepted background... As a result, our heritage, culture, and language were often kept private." (p. 12); "Negative stereotypes prompted her to conceal aspects of her heritage and avoid openly expressing it, which likely limited opportunities for using or sharing Persian in public settings." (p. 12)	Cognitively, the participant simultaneously recognizes the deep value of Iranian culture (pride) and perceives negative social stereotypes attached to it (shame). Affectively, there is explicit internal contradiction: pride and shame coexist ("even though I was proud... I sometimes felt ashamed"). Behaviorally, this contradiction produces an attitude-behavior gap: private endorsement of heritage language and culture coexists with public concealment ("kept private," "hide our Iranian culture," "claim a more accepted background"). Feedback maintains the ambivalent state: negative stereotypes limit public opportunities for Persian use, preventing resolution of the contradiction. This is the classic form of Structured Ambivalence.
2a	Alsahafi (2026)	Second-generation (G2) Hausa Saudi adults (maintenance-oriented majority)	Resistance	"The Hausa language is important and widely spoken worldwide. Learning it is essential for the Hajj and Umrah because it connects us to our people and original culture." (p. 3164); "Multilingualism is a richness of knowledge, and multiculturalism is a national richness" (p. 3164); "It is urgent and necessary to strengthen our connection to it and to teach it to our children." (p. 3163); "I wish special schools would be created to teach Hausa and English together." (p. 3164); "A moderate positive correlation was observed between Hausa proficiency and the perceived importance of speaking the language ($r=.32$, $p<0.01$)." (p. 3163)	Cognitively, G2 participants frame Hausa as important, internationally useful, and culturally valuable—especially for Hajj/Umrah communication—while recognizing multilingualism as a societal resource. Affectively, they express urgency and commitment to maintenance ("urgent and necessary"). Behaviorally, they actively advocate intergenerational transmission at home, call for dedicated schools and university programs, and prioritize teaching Hausa to children (AS6 $M=3.55$), expanding into institutional domains. Feedback is positive: higher Hausa proficiency correlates with greater perceived importance ($r=.32$), reinforcing maintenance motivation. Actual home use remains moderate due to parental-generation transmission gaps, but this is framed as a problem to be solved. This matches the Resistance pattern.
2b	Alsahafi (2026)	Third-generation (G3) Hausa Saudi adults (ambivalent/alienated majority)	Structured Ambivalence	"There is a weakening connection with our Hausa language. The current generation seems largely indecisive about speaking the language, and I fear that the next generation may witness its complete disappearance." (p. 3163); "Most [Hausa] people in their homes do not use it [Hausa]. I prefer to teach it to my children from a young age to keep them in touch with the language of their ancestors." (p. 3164); "It is crucial for parents and grandparents who speak Hausa to interact with their children and grandchildren in this language at home to prevent it from disappearing from our society." (p. 3164); G3 attitudinal measures: AS1 importance $M=3.08$, AS4 "not used at home" $M=2.50$, AS6 "should teach children" $M=3.32$ (p. 3162); "moderate negative correlations were identified concerning the belief that Hausa is not used at home ($r=-0.45$, $p<0.01$)" (p. 3163)	Cognitively, G3 participants hold contradictory evaluations: they endorse the value of intergenerational transmission (AS6 $M=3.32$) while rating Hausa's importance, unifying role, and international utility as neutral-to-low (AS1 $M=3.08$, AS2 $M=2.97$, AS3 $M=2.92$). Affectively, they experience simultaneous fear of language disappearance ("I fear that the next generation may witness its complete disappearance") and weakened connection ("weakening connection," "largely indecisive"). Behaviorally, they demonstrate domain-differentiated practice: advocating teaching children and establishing schools while actual home and community use is contracted (AS4 $M=2.50$). Feedback is mixed: the strong negative correlation between non-use at home and proficiency ($r=-.45$) indicates a negative cycle, while advocacy reflects residual maintenance motivation. This matches the Structured Ambivalence pattern.

No.	Author (Year)	Analytical Unit	Pattern Assigned	Original Excerpt – Pattern Evidence	Thematic Summary – Pattern Rationale
2c	Alsahafi (2026)	Participants endorsing language shift as natural/inevitable (Hassan, G2; Musa, G3)	Assimilation	"Many Hausa people experience deep pain... I find this frustration and pain to be unnecessary. It is a natural consequence of their circumstances... the Hausa language struggles to compete with the significance of English... There is little advantage for their children in learning their mother tongue if the father does not intend to return to his home country." (p. 3164–3165); "The Hausa language holds limited significance in Saudi Arabia. Learning it might be seen as a waste of time for those uninterested in their African heritage or who find it irrelevant to their professional goals." (p. 3165); G3: Arabic first language 70%, Hausa first language 29%; Hausa writing proficiency G3 M=1.80; generational gaps in listening (1.03) and speaking (1.18) (p. 3161)	Cognitively, these participants judge Hausa as low-status, non-competitive with English/Arabic, professionally irrelevant, and of "limited significance"; Hassan explicitly frames language shift as "a natural consequence" rather than a problem. Affectively, they show consistent disidentification: Hassan dismisses the pain of language loss as "unnecessary," and Musa describes learning Hausa as "a waste of time." Behaviorally, they endorse prioritizing dominant languages (English, Arabic) that "enable [children] to keep up with modern times," contracting Hausa use domains. Feedback is negative: broader quantitative data shows G3 has 70% Arabic first language, very low Hausa literacy (writing M=1.80), and significant generational decline, consistent with the assimilation trajectory. This matches the Assimilation pattern.
3a	Baranova (2026)	Older new speakers (elderly re-learners, e.g., Pavel, free-course pensioners, Ajsa/Bajerta historical cases)	Resistance	"When he became a grandfather, he decided that it was necessary to become a Kalmyk speaker." (p. 9–10); "these learners often have more free time and feel a sense of responsibility for preserving family traditions, including the ethnic language." (p. 9); "He began reading the newspaper in Kalmyk, Xal'məg Ünən, and his special interest is folklore texts or Buddhist rituals and terminology." (p. 9–10); "his wife... likes his efforts and is happy that she can speak Kalmyk with her husband (while previously they only spoke with each other in Russian)." (p. 9); "Students in the older age group told me that people around them evaluate their learning of Kalmyk positively and support their efforts." (p. 10)	Cognitively, older new speakers frame Kalmyk as essential to family traditions, intergenerational transmission, and ethnic identity. Affectively, they express a sense of responsibility and commitment to preservation ("it was necessary to become a Kalmyk speaker"). Behaviorally, they actively expand use domains: self-directed learning (reading newspapers, practicing pronunciation), enrolling in free language courses, university study, and increasing intra-family Kalmyk use—Pavel and his wife shifted from Russian-only to Kalmyk communication. Feedback is positive: family members support their efforts, and the community evaluates older learners' attempts positively, reinforcing continued use. Initial classroom embarrassment is a transient learning-phase affect, not a dominant contradictory orientation. This matches the Resistance pattern.

No.	Author (Year)	Analytical Unit	Pattern Assigned	Original Excerpt – Pattern Evidence	Thematic Summary – Pattern Rationale
3b	Baranova (2026)	Young Kalmyks in the Republic of Kalmykia (e.g., Batyr, Mergen, Sanal)	Structured Ambivalence	"the younger generation grows up with an attitude that attaches a high value to knowing Kalmyk" (p. 10); "Now I regret it... It's a shame I don't know my own language." (p. 11); "in our school, it was like, oh, Kalmyk, I'd rather learn English." (p. 11); "they participate in free language courses, as well as pay for private tutoring, listen to 'Oirat radio,' and try to organize some activities in Kalmyk." (p. 11); "I mainly communicate with two eeǯə [grandmothers], exchanging messages and sometimes managing to talk on the phone." (p. 11); "I decided to study on my own" after criticism at the speech club (p. 12); "young new speakers often face mixed or even negative attitudes" (p. 11); "people who know it well... criticize those who don't... look down on those who don't know it" (p. 11)	Cognitively, young Kalmyks hold contradictory evaluations: they attach high value to knowing Kalmyk as their native language, yet during schooling they prioritized English and globalization ("I'd rather learn English"). Affectively, they experience simultaneous regret and shame ("It's a shame I don't know my own language") alongside motivation to learn. Behaviorally, they demonstrate domain-differentiated practice: actively seeking learning opportunities (courses, private tutoring, Oirat radio, organizing activities) and using Kalmyk within supportive family contexts (with grandmothers), but avoiding public use after facing criticism—Sanal stopped speaking publicly after accent mockery and chose to study alone. Feedback is mixed: positive within the family but negative outside (fluent speakers criticize less proficient speakers), maintaining the ambivalent state. This matches the Structured Ambivalence pattern.
3c	Baranova (2026)	Kalmyk diaspora communities (Canada, US, Germany, Mongolia)	Resistance	"Emigration... brings new challenges and thoughts on identity, so young people tend to reconsider their sense of belonging. It often means that the will to speak one's native language becomes very strong." (p. 12); "somehow this Kalmyk inside me, it's just coming out." (p. 12); "we're getting together, the Kalmyk diaspora, and they even sing Kalmyk songs in restaurants, play the dombra" (p. 12); "Kalmyks in Mongolia said that they organized informal language clubs–meetings conducted in Kalmyk." (p. 13); "I want to organize a Kalmyk-language camp in Europe next year. Because kids want to socialize, and parents also want them to socialize with other Kalmyks." (p. 13); "in some cases, the diaspora proves more effective in preserving the language and finding innovative ways to transmit it, especially when family communication is limited or parental language competence is relatively low." (p. 13)	Cognitively, diaspora Kalmyks frame Kalmyk as the core of diasporic identity and the primary bond to ancestral heritage, while recognizing the challenge of a limited language environment abroad. Affectively, emigration intensifies the will to speak the native language ("the will to speak one's native language becomes very strong") and generates a visceral sense of identity belonging ("this Kalmyk inside me, it's just coming out"). Behaviorally, they actively expand use domains and create new institutional structures: community gatherings with Kalmyk singing and dombra music in restaurants, informal language clubs in Mongolia, and planned Kalmyk-language summer camps in Europe. Feedback is positive: diaspora communities provide external expectations and support that reinforce identity and language use, and in some cases the diaspora proves more effective at preservation than the homeland. This matches the Resistance pattern.

No.	Author (Year)	Analytical Unit	Pattern Assigned	Original Excerpt – Pattern Evidence	Thematic Summary – Pattern Rationale
4a	Beaudrie et al. (2019)	Spanish heritage language (SHL) students enrolled in a 14-week critical language awareness (CLA) course (n=19, pretest/posttest design)	Transition: from Structured Ambivalence toward Resistance via critical language awareness (CLA) instruction	**Pre-intervention:** "this group of learners showed a high level of CLA (4.6 out of 6) in the pretest" (p. 12); pretest Factor 3 (language maintenance) M=4.19, the lowest of the three factors (p. 15); "I would not codeswitch in front of my teachers because they may think I am less intelligent" (item 17, larger-sample M=3.88) (p. 15).<br>**Post-intervention:** "Paired-sample t-tests performed on the pretest and posttest scores showed significant post-instruction changes for factor 1 [t(18)=−2.38, p<.05], factor 2 [t(18)=−2.18, p<.05], and factor 3 [t(18)=−3.25, p<.05]" (p. 12); Factor 3 showed the largest increase (pretest M=4.19 → posttest M=4.61, d=.61) (p. 15).<br>**Theoretical framing:** "becoming critically language aware entails learners becoming cognizant of the naturalness of language variation... while accepting the intrinsic value of their own bilingual varieties... resisting dominant language ideologies by making their own decisions about language use and bilingualism... becoming critically aware involves positive behaviors toward maintenance and advocacy toward HLs" (p. 15–16).	**Pre-intervention state (Structured Ambivalence):** SHL students exhibited a "high cognition, moderate affect, low behavior" configuration. Cognitively, they showed high awareness of language variation and English hegemony (Factor 1 pretest M=4.91). Affectively, they held moderately positive attitudes toward bilingualism (Factor 2 M=4.67), though residual ambivalence remained (reluctance to codeswitch in front of teachers). Behaviorally, maintenance intentions were weakest (Factor 3 M=4.19), indicating an attitude-behavior gap.<br>**Post-intervention state (toward Resistance):** SHL students exhibited a "high cognition, moderate affect, low behavior" configuration. Cognitively, they showed high awareness of language variation and English hegemony (Factor 1 pretest M=4.91). Affectively, they held moderately positive attitudes toward bilingualism (Factor 2 M=4.67), though residual ambivalence remained (reluctance to codeswitch in front of teachers). Behaviorally, maintenance intentions were weakest (Factor 3 M=4.19), indicating an attitude-behavior gap.
5a	Callesano & Carter (2019)	Miami Latinx college students as perceivers of Spanish dialect variation (n=212)	Partial evidence – cannot assign full pattern	/	**Missing: participants' own behavioral dimension and feedback direction.** This is a perceptual dialectology study: participants rated recorded speakers' putative traits and behaviors, not their own. Cognitive and affective dimensions are present: participants hierarchically judge Peninsular Spanish as more correct/competent, yielding differential evaluative outcomes. However, all behavioral data measure perceptions of others (e.g., Colombian speakers perceived as English-dominant with low transmission), not participants' own language use. No feedback loop is documented. A full pattern therefore cannot be assigned. The study supports the Cognition Shaping Affect pathway but not a complete cognitive-affective-behavioral configuration.

No.	Author (Year)	Analytical Unit	Pattern Assigned	Original Excerpt – Pattern Evidence	Thematic Summary – Pattern Rationale
6a	Fatima & Nadeem (2025)	Older generations (grandparents/elders) in multigenerational Pakistani households	Resistance	"Older generations, often the [grandparents], prioritize their ethnic and heritage languages over other culturally dominant languages." (p. 12); "Older generations play a key role in transmitting and maintaining heritage languages through storytelling, ritual [and cultural practices]." (p. 13); "their direct influence fosters intergenerational language maintenance and learning—whether through [cultural transmission]." (p. 12); "A strong positive correlation exists between Language Practices (M=2.75, SD=1.259) and Ethnic Identity (r=0.628), illustrating higher levels of engaging in [heritage language use correspond to] stronger ethnic identity and ethnic attachment. The p-value (p<0.01) confirms this correlation is statistically significant." (p. 10–11); "individuals find themselves closely [connected to] language and ethnic identity as demonstrated by existing [ethnolinguistic identity theory]." (p. 12)	Cognitively, older generations frame heritage languages (Punjabi, Siraiki, Pashto, Hindko, etc.) as the primary marker of ethnic identity and cultural continuity, prioritizing them over culturally dominant languages (Urdu, English). Affectively, they exhibit strong, consistent positive affective investment: quantitative data confirm a strong positive correlation between language practices and ethnic identity (r=0.628, p<0.01), and qualitative data show elders view heritage language transmission as a familial and cultural responsibility. Behaviorally, they actively expand heritage language use into family and cultural domains: prioritizing heritage language in intergenerational communication, transmitting language through storytelling and religious/cultural rituals, and directly fostering younger generations' learning. Feedback is positive: greater heritage language practice correlates with stronger ethnic identity attachment (r=0.628), motivating continued use and transmission. This matches the Resistance pattern.
6b	Fatima & Nadeem (2025)	Younger generations (children/grandchildren) in Pakistani multigenerational households	Structured Ambivalence	"younger generations prefer dominant languages such as English—[for the professional and societal advantages of heritage languages]." (p. 12); "Code-switching emerges as a significant practice amongst the younger generations [as they navigate intergenerational communication, contextual use of languages]." (p. 1); "the younger generation does not have the same [level of attachment to their] ethnic heritage as the older generation." (p. 12); "the younger generation often preferred the culturally dominant language." (p. 8); P5 (Siraiki speaker): "Whenever there was violence, it was always Siraiki... I feel more connected whenever I [speak Siraiki with family]." (p. 8–9); "A moderate positive correlation is also observed between Ethnic Identity and Attitudes and Perceptions (r=0.481), suggesting that ethnic identity influences attitudes toward heritage [language maintenance]." (p. 11)	Cognitively, younger generations hold contradictory evaluations: they recognize heritage languages as markers of ethnic identity (ethnic identity–attitudes correlation r=0.481), yet simultaneously prioritize dominant languages—especially English—for perceived professional advantages. Affectively, their orientation is mixed: they retain some attachment to heritage languages, but this is demonstrably weaker than that of the older generation; P5 (Siraiki speaker) illustrates extreme affective contradiction, associating Siraiki simultaneously with violence and with connection. Behaviorally, they exhibit domain-differentiated code choice: code-switching is "a significant practice" as they navigate between heritage language use with elders and dominant language use in professional/educational contexts; heritage language proficiency and use are contracted. Feedback is mixed: heritage language practice still correlates positively with ethnic identity (r=0.628 overall), but reduced use leads to lower proficiency and weaker identity attachment, while residual identity attachment prevents full assimilation. This matches the Structured Ambivalence pattern.

No.	Author (Year)	Analytical Unit	Pattern Assigned	Original Excerpt – Pattern Evidence	Thematic Summary – Pattern Rationale
7a	Gao & Liu (2025)	Wa (佤族) individuals with strong minority identity in Ximeng County, Yunnan	Resistance	"Wa individuals' minority identity is 130/140" (p. 10–11); "minority identity represents a desire to maintain one's heritage language as a marker of cultural or group identity" (p. 3); "minority Wa individuals speak only minority Wa language" (p. 10–11); "when minority identity is strong, individuals tend to choose and persist in using [the minority language]" (p. 3); "self-minority identity transmission significantly fosters minority language diffusion in both heterogeneous and homogeneous networks, especially in heterogeneous networks" (p. 1)	Cognitively, the Wa's exclusive use of Wa language implies endorsement of its legitimacy and identity value; the model frames strong minority identity as paired with recognition of the heritage language as a group marker. Affectively, Wa individuals exhibit exceptionally strong minority identity (130/140 above threshold), reflecting a desire to maintain their heritage language. Behaviorally, they engage in exclusive use of the Wa language, resisting adoption of other languages. Feedback is positive: self-minority identity transmission through social networks significantly fosters Wa language diffusion, and higher network connectivity further promotes it. This matches the Resistance pattern: consistent positive identity, exclusive behavioral maintenance, and a positive identity–practice feedback cycle.
7b	Gao & Liu (2025)	Lahu (拉祜族) individuals with weak minority identity in Ximeng County, Yunnan	Partial evidence – cannot assign full pattern	"minority Lahu with minority identity of 6 or more accounted for 7/37" (p. 10–11); "minority Lahu individuals are open to the minority Wa language" (p. 10–11); "when identity is weak, individuals are more likely to choose other languages" (p. 3)	**Missing: direct cognitive evidence of heritage language devaluation and explicit behavioral evidence of contraction or shift.** Affective evidence is present: Lahu individuals show weak minority identity (only 7/37 above threshold) and are described as "open to the minority Wa language." The model predicts that weak identity leads individuals to "choose other languages," but this is a theoretical prediction rather than empirically verified Lahu behavior. No direct evidence documents the Lahu's own cognitive evaluation of heritage language legitimacy or their actual language use contraction. A full pattern therefore cannot be assigned.
8a	Gavriilidou & Mitits (2021)	First-generation Greek heritage language speakers in Chicago	Resistance	"the first generation prefer and maintain Greek as the language they still feel more proficient in" (p. 88); "First-generation HSs demonstrate high proficiency in Greek and claim they are Greek because they were born and raised in Greece" (p. 90); "are active members of the Greek community of Chicago, and go to local Greek-orthodox churches every week" (p. 90); "have a sense of belonging to and love of Greece" (p. 90); "the more their parents have engaged them in cultural and social activities with the Greek community and culture, the higher sense of appreciation for Greek language and culture the children have" (p. 90–91)	Cognitively, first-generation speakers treat Greek as the defining marker of identity, claiming Greekness through birth and upbringing in Greece. Affectively, they exhibit strong belonging to and love of Greece, with high community engagement. Behaviorally, they prefer and maintain Greek as their more proficient language, attend Greek Orthodox churches weekly, and actively transmit Greek to children via community schools. Feedback is positive: parental cultural engagement predicts children's higher appreciation of Greek language and culture, reinforcing the maintenance cycle. This matches the Resistance pattern: consistent positive identity, active behavioral maintenance, and a positive practice–identity feedback loop.

No.	Author (Year)	Analytical Unit	Pattern Assigned	Original Excerpt – Pattern Evidence	Thematic Summary – Pattern Rationale
8b	Gavriilidou & Mitits (2021)	Third-generation Greek heritage language speakers whose parents did not invest in cultural maintenance, in Chicago	Assimilation	"in cases where second-generation parents did not invest in the preservation of Greek culture, sometimes due to their workload or because one of the two parents is not Greek, third-generation children feel mainly American, distance themselves from Greek customs, traditions, and/or cuisine, and very often demonstrate a low proficiency in Greek" (p. 91); "they don't go to Greek school" (p. 91); "since language proficiency in the HL is low for third-generation HSs, the Greek orthodox religion seems to be the defining element of Greek identity for this generation" (p. 91); "a decline in the use of Greek as HSs age and (b) an extended use of [English]" (p. 85)	Cognitively, third-generation speakers identify primarily as American, with Greek Orthodox religion replacing language as the identity marker. Affectively, they distance themselves from Greek customs, traditions, and cuisine, indicating weakening heritage attachment. Behaviorally, they demonstrate low Greek proficiency, do not attend Greek schools, and show declining Greek use with age, reflecting contracted heritage language domains. Feedback is negative: reduced parental investment → lower proficiency → American identification → further distance from Greek culture. This matches the Assimilation pattern: consistent affective disidentification with heritage language, behavioral contraction, and a negative attrition cycle.
9a	Grgič & Guardiancich (2025)	Slovenian minority in Italy during the družbeno gospodarstvo (social economy) period, 1945–1991	Resistance	"the community's leadership established a minority-language-only environment with a social and economic model focused, among other purposes, on preserving the language and protecting it from assimilation" (p. 27); "local varieties, from narrower dialectal to broader regional ones, are considered entirely and exclusively acceptable in all settings" (p. 30); "supra-regional standard and non-standard varieties as something foreign, remote, even undesirable and non-adequate" (p. 30); "speakers' attitude towards their own language is dominated by a sense of pride" (p. 39); "the entrenchment of a local-language ideology, which still inhibits the use of different varieties, encouraging the exclusive use of the local standard" (p. 36)	Cognitively, the community revalued local Slovenian varieties as the exclusively acceptable code in all settings, while dismissing supra-regional standards as foreign and undesirable. Affectively, speakers' attitudes were dominated by a sense of pride, reinforced by a language-pride ideology that distinguished Italian Slovenians from Slovenians in Slovenia. Behaviorally, the leadership established a minority-language-only environment through a closed social and economic network (družbeno gospodarstvo), enforcing exclusive use of the local standard across schooling, economy, and social life. Feedback was positive: the tight, impermeable network reinforced the local-language ideology, which further encouraged exclusive use and pride. This matches the Resistance pattern: consistent positive identity, active institutionalized maintenance, and a positive practice–ideology feedback loop.
9b	Grgič & Guardiancich (2025)	Slovenian minority in Italy after the collapse of družbeno gospodarstvo, post-1991	Partial evidence – cannot assign full pattern	"the družbeno gospodarstvo effectively collapsed during the 1990s, after the dissolution of Yugoslavia, its main external sponsor" (p. 34); "there is a general decline in the communicative competence of Slovenian-language speakers in both local and supra-regional standard and non-standard varieties" (p. 29); "Despite being educated in Slovenian from pre-school to pre-academic level, the working [place use declined]" (p. 29); "the local economy... have continued to decline since 1991, making the local network even more impermeable" (p. 38); "speakers' attitude towards their own language is dominated by a sense of pride" (p. 39)	**Missing: consistent cognitive or affective evidence of disidentification with the heritage language.** Cognitive and affective dimensions remain positive: the local-language ideology still inhibits use of other varieties, and speakers' attitudes are still dominated by pride. However, the behavioral dimension shows contraction: general decline in communicative competence across all varieties, and reduced use in the workplace despite Slovenian-medium education. The three dimensions are inconsistent—positive cognition/affect coexist with behavioral attrition—so no single stable pattern can be assigned. This may represent a transitional state between Resistance and Assimilation, but explicit evidence of cognitive or affective shift is absent.

No.	Author (Year)	Analytical Unit	Pattern Assigned	Original Excerpt – Pattern Evidence	Thematic Summary – Pattern Rationale
10a	Inan & Harris (2025)	Immigrant families who view bilingualism as a socioeconomic asset (asset-oriented families)	Resistance	"Many immigrant parents reveal a strong desire to preserve their heritage languages, as they perceive language as an emotional and symbolic bridge to their cultural identity" (p. 1); "Some families view bilingualism as a socioeconomic asset that can enhance career prospects and global competitiveness" (p. 2–3); "actively seek community and digital resources" (p. 3); "Participation in bilingual programs has been shown to foster students' cultural identity and linguistic confidence" (p. 3)	Cognitively, these families revalue bilingualism as a socioeconomic asset enhancing career prospects and global competitiveness, while also recognizing heritage language as an emotional and symbolic bridge to cultural identity. Affectively, parents reveal a strong desire to preserve their heritage languages, reflecting positive attachment. Behaviorally, they actively seek community and digital resources and enroll children in bilingual programs, expanding heritage language use beyond the home. Feedback is positive: participation in bilingual programs fosters cultural identity and linguistic confidence, reinforcing maintenance motivation. This matches the Resistance pattern: consistent positive identity, active resource-seeking and program participation, and a positive practice–identity feedback loop.
10b	Inan & Harris (2025)	Immigrant families facing economic pressures who deprioritize heritage language due to employment concerns (concern-oriented families)	Structured Ambivalence	"Many immigrant parents reveal a strong desire to preserve their heritage languages, as they perceive language as an emotional and symbolic bridge to their cultural identity" (p. 1); "others—particularly those facing economic pressures—may prioritize dominant language acquisition for perceived [benefits]" (p. 3); "where societal attitudes and employment concerns lead many parents to deprioritize heritage [language use]" (p. 3); "FLP alone cannot account for the various sociopolitical and economic pressures that influence families' language practices and beliefs" (p. 1)	Cognitively, these families simultaneously endorse the heritage language as an emotional and symbolic bridge to cultural identity and the dominant language as essential for employment and economic mobility, holding contradictory judgments about both. Affectively, they experience a strong desire to preserve the heritage language alongside anxiety arising from employment concerns and societal attitudes, indicating internal affective contradiction. Behaviorally, they deprioritize heritage language use in family language policy and prioritize dominant language acquisition, reflecting a domain shift toward the majority code while not fully abandoning heritage maintenance. Feedback maintains the ambivalent state: economic pressures sustain the priority on dominant language, while identity attachment prevents complete shift, so the contradiction is managed rather than resolved. This matches the Structured Ambivalence pattern: contradictory cognitive evaluations, coexisting attachment and anxiety, and behaviorally differentiated priority setting.

No.	Author (Year)	Analytical Unit	Pattern Assigned	Original Excerpt – Pattern Evidence	Thematic Summary – Pattern Rationale
11a	Kovacs Rac & Halupka -Rešetar (2018)	Hungarian minority 5th and 8th grade pupils in Vojvodina, Serbia (total sample)	Structured Ambivalence	"their dialect marks their local identity, i.e. their sense of belonging to the community" (p. 125); "A dialect is, thus, a symbol of a community's sense of belonging and of the sense of local identity" (p. 126); "I like it but I do not use it often because I think that I might be judged" (p. 131); "Only 10.33% of the pupils (also) stated that they liked their dialect but used it rarely due to fear of being judged. Such an attitude conveys the covert prestige of the dialect" (p. 132); "A total of 37.24% of the respondents reported having been stigmatized in some way because of their dialect or use of language. In spite of this, as many as 77.24% considered their dialect attractive" (p. 137); "they find it attractive and even though some of them have encountered other people's negative attitudes toward their dialects, they nevertheless hold a positive opinion of them" (p. 138)	Cognitively, pupils recognize the dialect as a marker of local identity and community belonging, while standard Hungarian remains the legitimate code in formal and public domains, indicating simultaneous endorsement of both. Affectively, 77.24% consider the dialect attractive and hold positive opinions, yet 37.24% have experienced stigmatization, generating anxiety and fear of judgment. Behaviorally, the sample exhibits a clear attitude-behavior gap: 10.33% explicitly state they like the dialect but avoid public use due to fear of judgment, reflecting domain-differentiated code choice (dialect in private/community settings, standard in public). Feedback maintains the ambivalent state: stigmatization sustains public-use avoidance, while private use and covert prestige preserve positive attachment, so the contradiction is managed rather than resolved. This matches the Structured Ambivalence pattern: contradictory affective orientations (attachment + anxiety), an explicit attitude-behavior gap, and domain-differentiated behavioral performance.
12a	Li (2026)	Majority of Chinese migrant families in Italy (4/6 cases: Yang, Aurora, An, Shu) adopting exclusive Putonghua use and marginalising fangyan	Assimilation	"At home, we basically speak Putonghua and not fangyan […] We can speak fangyan, but our child can't, and we don't want to teach him, because it feels like he's already learning enough. Fangyan is not particularly useful for him anyway. Putonghua is already enough in China […] Between Putonghua and fangyan, I would choose Putonghua, because it can be spoken all over China, whereas fangyan is only used in one place and can't be used elsewhere" (p. 8–9); "At home we only speak Putonghua, because she no longer understands Wenzhouhua. Besides, children in China nowadays don't speak Wenzhouhua anymore […] If no one is learning fangyan anymore, why would you learn it in Italy?" (p. 9); "I don't think fangyan is that important. Chinese is important, because we are Chinese—one must be able to speak Chinese […] As for fangyan, it is only spoken in Qingtian, and fewer and fewer people are using it now. It is also important that her pronunciation may become less standard" (p. 9–10)	Cognitively, these families judge Putonghua as the standard, universally useful, and legitimate heritage code, while dismissing fangyan as geographically limited, useless outside its region, declining in relevance even within China, and potentially interfering with standard pronunciation. Affectively, they express dismissiveness toward fangyan ("not particularly useful," "I don't think fangyan is that important") while investing identity in Putonghua as the carrier of Chineseness ("we are Chinese—one must be able to speak Chinese"). Behaviorally, they adopt exclusive Putonghua at home, actively refuse to teach fangyan, and relegate it to passive comprehension or complete abandonment, contracting fangyan's domains of use. Feedback is negative: reduced fangyan use → children's passive or zero competence → parents' judgment that fangyan is unnecessary → further marginalisation, interrupting intergenerational transmission. This matches the Assimilation pattern: consistent cognitive devaluation and affective distancing from fangyan, coupled with behavioral contraction and interrupted transmission.

No.	Author (Year)	Analytical Unit	Pattern Assigned	Original Excerpt – Pattern Evidence	Thematic Summary – Pattern Rationale
12b	Li (2026)	Zhen family (one case) maintaining fangyan as the primary home language while simultaneously valuing Putonghua	Structured Ambivalence (domain-differentiated subtype)	"At home we generally speak our own fangyan with him […] He understands everything, but he can't speak it. He speaks Putonghua with me […] Learning fangyan is mainly for parents; learning Chinese is for the future […] I insist on speaking my hometown language with him: it's rarely used outside, so it's hard to learn. Putonghua can easily be heard outside, can be watched on the phone, so there's no worry about not learning it […] But I still think fangyan is also very important, because after all there are still elderly people. My thinking is a bit traditional […] I think he should learn some of the hometown language as well as Putonghua—both matter" (p. 10–11)	Cognitively, this family simultaneously endorses fangyan as valuable for intergenerational continuity and family ties ("learning fangyan is mainly for parents," "there are still elderly people") and Putonghua as essential for future opportunities ("learning Chinese is for the future"), holding both as legitimate without explicit conflict. Affectively, the mother expresses attachment to fangyan through active insistence ("I insist on speaking my hometown language") and traditional family values, while instrumentally acknowledging Putonghua's practical importance—both orientations coexist without apparent internal contradiction. Behaviorally, fangyan is used by the mother in the home domain (child has passive competence), while Putonghua serves as the child's active code and the language of future-oriented learning, reflecting stable domain-differentiated code choice. Feedback maintains the differentiated configuration: home exposure preserves fangyan as a family/identity marker, while external exposure sustains Putonghua as a tool, so the functional separation is reinforced rather than resolved. This matches the Structured Ambivalence domain-differentiated subtype: coexisting positive evaluations of both varieties, stable contextual allocation, and no explicit attitudinal conflict.
13a	Makarova et al. (2019)	Russian-speaking migrant families in Saskatchewan, Canada (total sample, N=30)	Resistance	"Overall, the parents demonstrated strong positive attitudes toward Russian language and culture. They show no desire to be 'assimilated,' and want Russian language maintained in the family" (p. 466); "As a person with Russian-speaking ancestry, it is important to be able to speak Russian" (25/30 strongly agree, 5/30 agree) (p. 466); "There is no use in my child's learning Russian in Canada" (18/30 strongly disagree, 8/30 disagree) (p. 467); "We came to Canada to become Canadian, so I do not want my child to maintain Russian" (20/30 strongly disagree, 7/30 disagree) (p. 467); "with two exceptions, Russian-speaking parents report the use of 'only Russian' or 'mostly Russian' at home with their children" (38% only Russian, 55% mostly Russian) (p. 468); "Russian-speaking parents not only 'vote' for bilingualism on paper, but also create sufficient language input for their children" (p. 476); "with the right determination and language attitudes, parents can pass a heritage language on to children even in a highly unfavorable environment" (p. 476)	Cognitively, parents revalue Russian as core to identity (100% agree that "as a person with Russian-speaking ancestry, it is important to be able to speak Russian") and reject the judgment that Russian is useless in Canada (90% disagree with "there is no use in my child's learning Russian in Canada"). Affectively, they demonstrate strong positive attitudes toward Russian language and culture, explicitly "show no desire to be 'assimilated,'" and overwhelmingly reject assimilationist framing (90% disagree with "we came to Canada to become Canadian, so I do not want my child to maintain Russian"). Behaviorally, they actively maintain Russian through intensive home use (93% report "only Russian" or "mostly Russian" at home) and institutional support via the Saskatoon Russian Language Saturday School, expanding heritage language use beyond passive exposure. Feedback is positive: strong anti-assimilationist attitudes → rich Russian language input at home → children's proficiency comparable to monolingual peers → reinforcement of parental maintenance commitment. This matches the Resistance pattern: consistent positive identity investment, active behavioral maintenance through home and institutional channels, and a positive attitude–practice feedback cycle.

No.	Author (Year)	Analytical Unit	Pattern Assigned	Original Excerpt – Pattern Evidence	Thematic Summary – Pattern Rationale
14a	Prada (2019)	Latinx heritage Spanish students in a university SHL classroom (N=17, 8 interviewed)	Transition: from Structured Ambivalence toward Resistance via translanguaging pedagogy	Pre-intervention: "Growing up in a school with lots of Hispanics I did use Spanglish but I was told by the teachers and other students that couldn't understand Spanish to only use English, so in most of high school I only used English" (p. 314); "I was very embarrassed speaking to others because my Spanish was not perfect but was expected since I am Hispanic" (p. 314); Post-intervention: "this class has given me a sort of pride or acceptance of my Spanglish. Before, I would feel ashamed of not knowing the language as well as I should but now it's shifted into feeling like 'well, I know how to speak two languages fairly well instead of just knowing how to speak only one'" (p. 317); "I have overcome my embarrassment of Spanglish . . . even, like . . . my fear of saying something stupid" (p. 319); "Spanglish belongs in the classroom because people think in different ways" (p. 318); "the shifts from considering the use of Spanglish and flexible bilingual practices as faulty and inadequate to rethinking them as valuable and purposeful" (p. 320)	This unit documents a deliberate attitudinal transformation triggered by translanguaging pedagogy. Pre-intervention, students exhibited Structured Ambivalence: cognitively, they accepted monoglossic ideologies framing Spanglish as "faulty and inadequate" while simultaneously using it in informal settings; affectively, they experienced shame and embarrassment about their Spanish ("I was very embarrassed," "I would feel ashamed"); behaviorally, they suppressed Spanglish in school and adopted exclusive English ("in most of high school I only used English"), reflecting a private-use/public-concealment split. Post-intervention, the configuration shifted toward Resistance: cognitively, students revalued Spanglish as a legitimate communicative resource ("Spanglish belongs in the classroom") and challenged the assumption of a single ideal standard; affectively, shame transformed into pride and acceptance ("a sort of pride or acceptance of my Spanglish," "I have overcome my embarrassment"); behaviorally, they began deploying flexible bilingual practices in academic settings. The transition is mediated by translanguaging pedagogy, which creates a sociolinguistic frame normalising mixed practices. This is coded as Transition rather than a stable pattern because the article explicitly documents pre/post change linked to a deliberate intervention.
15a	Shabtaev et al. (2026)	Mountain Jews in Israel (three generations, both Juhuri and Russian)	Assimilation	"for the Israeli respondents, there is a decrease from G1 to G2 for all three Attitude components for both Juhuri and Russian" (p. 627–628); "in Israel attitudes toward Juhuri and Russian decline across generations for the three attitudinal components" (p. 631); "Strong correlations between ethnic identity and language attitudes were found for both heritage languages in Israel" (p. 620); "In Israel, all correlations between EI and language attitudes were significant, ranging from 0.20–0.47 for both languages" (p. 629)	Cognitively, Hebrew's dominant status in Israel overshadows both Juhuri and Russian, reducing their perceived legitimacy and utility across generations. Affectively, all three attitudinal components (affective, behavioral, cognitive) for both heritage languages show a consistent decline from G1 to G2, indicating progressive affective distancing. Behaviorally, the declining behavioral component reflects contracting domains of heritage language use and weakening intergenerational transmission. Feedback is negative: reduced use across generations → lower perceived importance → greater affective distance → further attrition, a pattern consistent with generational assimilation. Although ethnic identity remains strong and correlates with attitudes, the absolute attitudinal decline across all components indicates that identity investment alone cannot counteract the assimilatory pressure of Hebrew dominance. This matches the Assimilation pattern: consistent cross-generational decline across all three dimensions for both heritage languages.

No.	Author (Year)	Analytical Unit	Pattern Assigned	Original Excerpt – Pattern Evidence	Thematic Summary – Pattern Rationale
15b	Shabtaev et al. (2026)	Mountain Jews in the United States (Juhuri, three generations)	Resistance	"For the United States respondents, in contrast, there is no decline across the three generations" (p. 628); "Attitudes to Juhuri rises across generations for the Affective component" (p. 631); "In the United States, EI measures strongly correlate with Juhuri attitudes but not with Russian attitudes" (p. 631); "In the United States, correlations between EI and language attitudes were significant only for Juhuri, ranging from 0.31–0.44" (p. 629); "the revival of ethnic identity among second-generation immigrants" (p. 620)	Cognitively, Juhuri is revalued as the sole carrier of Mountain Jewish heritage in the US context, where it is not overshadowed by a dominant co-ethnic language (unlike Hebrew in Israel). Affectively, attitudes toward Juhuri remain stable across generations and the affective component actually rises among G2, reflecting strengthened attachment and pride; the revival of ethnic identity among second-generation immigrants fuels this positive orientation. Behaviorally, the stable behavioral component indicates maintained heritage language practices and intergenerational transmission, supported by strong ethnic identity commitment. Feedback is positive: strong ethnic identity → positive affective investment → maintained behavioral practice → reinforcement of Juhuri's identity value. This matches the Resistance pattern: consistent positive affect, identity-driven maintenance, and a positive identity–attitude–practice feedback cycle, with Juhuri functioning as the irreplaceable core of Mountain Jewish identity in the diaspora.
15c	Shabtaev et al. (2026)	Mountain Jews in the United States (Russian, three generations)	Assimilation	"but in the United States the connection between Russian and Mountain Jewish identity was lost across generations" (p. 620); "In the United States, correlations between EI and language attitudes were significant only for Juhuri, ranging from 0.31–0.44, but not for Russian" (p. 629); "For Russian in the United States, the significance of the correlations declines across generations, to the point of no significant correlations between EI and attitudes among G2 participants" (p. 629); "Attitudes to Russian are most positive among G1 in comparison to the other two generations, although not significantly so" (p. 631)	Cognitively, Russian becomes decoupled from Mountain Jewish identity in the US context, as Juhuri assumes the role of the primary heritage identity marker; Russian is increasingly perceived as a general immigrant language rather than an identity core. Affectively, attitudes toward Russian are most positive among G1 but show a generational weakening, with no significant differences across generations reflecting a gradual rather than abrupt decline; the loss of identity correlation by G2 indicates affective disidentification. Behaviorally, the declining behavioral component across generations reflects contracting use domains and weakening transmission, as Russian is deprioritised relative to both English and Juhuri. Feedback is negative: identity decoupling → reduced affective investment → behavioral contraction → further identity distancing. This matches the Assimilation pattern: progressive disidentification from Russian across generations, with the critical marker being the complete loss of correlation between ethnic identity and Russian attitudes among G2.

No.	Author (Year)	Analytical Unit	Pattern Assigned	Original Excerpt – Pattern Evidence	Thematic Summary – Pattern Rationale
16a	Siebenhütter (2021)	Young Kui speakers in northeastern Thailand (19–25 age group, overall sample)	Structured Ambivalence (domain-differentiated subtype)	"less than 50% of the 19–25 age group use Kui at home, even though more than 70% of females and more than 50% of males stated that their mother tongue is Kui" (p. 17); "Young speakers speak Thai under certain situations, but maintain the Kui language to acknowledge the unique heritage and value of their minority language" (p. 18); "Many speakers still use Kui at home and outside of school (e.g. at the market or within their respective communities) and only shift to Thai or other languages when they need to communicate with people from different backgrounds" (p. 18); "Such generational patterns do not demonstrate a shift from Kui to Thai" (p. 18); "a Kui speaker did not mention his Kui background at his workplace in Bangkok, fearing negative consequences" (p. 19); "Only a few speakers in the 19–25 age group (6%) believe that it would be good if Kui completely disappeared, while the majority of the speakers in all of the age groups do not like the idea of the language disappearing" (p. 15–16)	Cognitively, young Kui speakers simultaneously endorse Thai as the legitimate medium of instruction and practical public code while valuing Kui as the carrier of "unique heritage and value of their minority language," holding both as legitimate without explicit internal conflict. Affectively, the majority express positive attachment to Kui (only 6% welcome its disappearance) while acknowledging its lower social status and the risk of stigmatisation, as evidenced by one speaker concealing his Kui background in Bangkok "fearing negative consequences." Behaviorally, they exhibit stable domain-differentiated code choice: Kui is maintained at home, in the market, and within the community, while Thai is used at school and with people from different backgrounds; the author explicitly states this "does not demonstrate a shift from Kui to Thai." Feedback maintains the differentiated configuration: community use preserves Kui as an identity marker, while school exposure sustains Thai as a tool, so the functional separation is reinforced rather than resolved. This matches the Structured Ambivalence domain-differentiated subtype: coexisting positive evaluations of both languages, stable contextual allocation, and no explicit attitudinal conflict, with the concealment case representing a secondary attitude-behavior gap manifestation.
17a	Vari & Tamburelli (2023)	Moselle Franconian vernacular speakers in Éislek, Luxembourg (endogenous, linguistically close standard: Standard Luxembourgish)	Partial evidence – cannot assign full pattern (cognitive + affective evidence present; behavioral dimension missing; configuration points toward Resistance)	"creating a 'new' endogenous standard (Luxembourg)" (p. 459); "Luxembourgish average D-scores were positive (mean = + 0.28), showing a preference for the vernacular over the standard" (p. 458); "A close standard would be perceived to resemble the vernacular variety and therefore share its positive evaluation" (p. 459); "speech community was the strongest of all predictor variables, suggesting that how endangered vernaculars are standardised influences attitudes towards them more than any other sociolinguistic factor such as age or gender" (p. 459)	Cognitively, the creation of an endogenous, linguistically close standard (Standard Luxembourgish) revalues the vernacular by sharing the standard's positive evaluation with it, enhancing its perceived legitimacy. Affectively, the positive implicit D-score (+0.28) demonstrates that speakers associate the vernacular more strongly with positive evaluations than the standard, reflecting consistent positive affective orientation. Behaviorally, this study measures implicit attitudes via IAT and self-assessed competence but does not provide direct verbatim evidence of behavioral maintenance practices (e.g., domain expansion, intergenerational transmission, institutional advocacy), so the behavioral dimension cannot be fully verified. Feedback is inferred to be positive: close endogenous standard → positive implicit attitudes → likely maintenance, but this remains an inference rather than directly evidenced. The available cognitive and affective evidence points toward the Resistance pattern, but the missing behavioral dimension prevents a full pattern assignment.

No.	Author (Year)	Analytical Unit	Pattern Assigned	Original Excerpt – Pattern Evidence	Thematic Summary – Pattern Rationale
17b	Vari & Tamburelli (2023)	Moselle Franconian vernacular speakers in Belgische Eifel, Belgium (exogenous, linguistically distant standard: Standard German)	Partial evidence – cannot assign full pattern (cognitive + affective evidence present; behavioral dimension missing; configuration points toward Assimilation)	"associating the vernacular varieties with an exogenous standard (Belgium)" (p. 459); "Belgian participant D-scores were negative (mean = −0.31), indicating a preference for the standard over the vernacular" (p. 458); "a distant standard would be perceived to not resemble the vernacular, not sharing its positive attitudes" (p. 459); "speech community was the strongest of all predictor variables, suggesting that how endangered vernaculars are standardised influences attitudes towards them more than any other sociolinguistic factor such as age or gender" (p. 459)	Cognitively, reliance on an exogenous, linguistically distant standard (Standard German) fails to transfer positive evaluation to the vernacular, as the distant standard is perceived not to resemble the vernacular, thereby undermining its perceived legitimacy. Affectively, the negative implicit D-score (−0.31) demonstrates that speakers associate the standard more strongly with positive evaluations than the vernacular, reflecting consistent negative affective orientation toward the vernacular relative to the standard. Behaviorally, this study measures implicit attitudes via IAT and self-assessed competence but does not provide direct verbatim evidence of behavioral contraction (e.g., domain shrinkage, interrupted transmission, shift to standard), so the behavioral dimension cannot be fully verified. Feedback is inferred to be negative: distant exogenous standard → negative implicit attitudes → likely contraction, but this remains an inference rather than directly evidenced. The available cognitive and affective evidence points toward the Assimilation pattern, but the missing behavioral dimension prevents a full pattern assignment.
18a	Yiakoumetti (2022)	Teachers in UK Chinese community schools (heritage Mandarin classrooms, British-born Chinese students)	Structured Ambivalence (domain-differentiated subtype)	"All teachers systematically reverted to students' dominant language, English, to introduce and explain new vocabulary and grammatical structures" (p. 5); "Teachers' language choice was based on their belief that first-language provision facilitated the acquisition of new linguistic knowledge" (p. 5); "In their efforts to build rapport and assist students with personal matters (i.e., support beyond academic matters), teachers purposefully employed students' first language" (p. 8); "students associated the second language with teaching and the first and English language with more familiar, casual and comfortable settings" (p. 8–9); "Sometimes I use more English than I would like" (teacher comment, p. 9); "If we use more Chinese, the children will not understand" (teacher comment, p. 9); "systematic L1 integration into heritage instruction reinforced the cognition that L1 is a valid pedagogical resource" (p. 10–11)	Cognitively, teachers simultaneously endorse L2 (Mandarin) as the legitimate teaching target and L1 (English) as a valid and necessary pedagogical resource, holding both as functional rather than contradictory. Affectively, teachers express mild ambivalence—they acknowledge using more English than ideal ("Sometimes I use more English than I would like") but rationalise it as necessary given students' low proficiency ("If we use more Chinese, the children will not understand"), without explicit internal conflict or shame. Behaviorally, they exhibit stable domain-differentiated code choice: L2 is associated with formal teaching and instructional goals, while L1 is used for explanation of new vocabulary/grammar, rapport-building, personal matters, and classroom management—functional allocation rather than behavioral contraction or expansion. Feedback maintains the differentiated configuration: systematic L1 use → reinforced cognition that L1 is a valid pedagogical resource → continued functional separation, so the domain allocation is stabilised rather than resolved. This matches the Structured Ambivalence domain-differentiated subtype: coexisting functional evaluations of both languages, stable contextual allocation, and no explicit attitudinal conflict, with the mild teacher guilt representing a secondary affective nuance rather than core ambivalence.
